# Supplementary figures and images for: Magnitude and clinical characteristics of cerebral palsy among children in Africa: A systematic review and meta-analysis
Source: PLOS Glob Public Health. 2024 Jun 21;4(6):e0003003. doi: 10.1371/journal.pgph.0003003 (PMC11192420; doi:10.1371/journal.pgph.0003003)

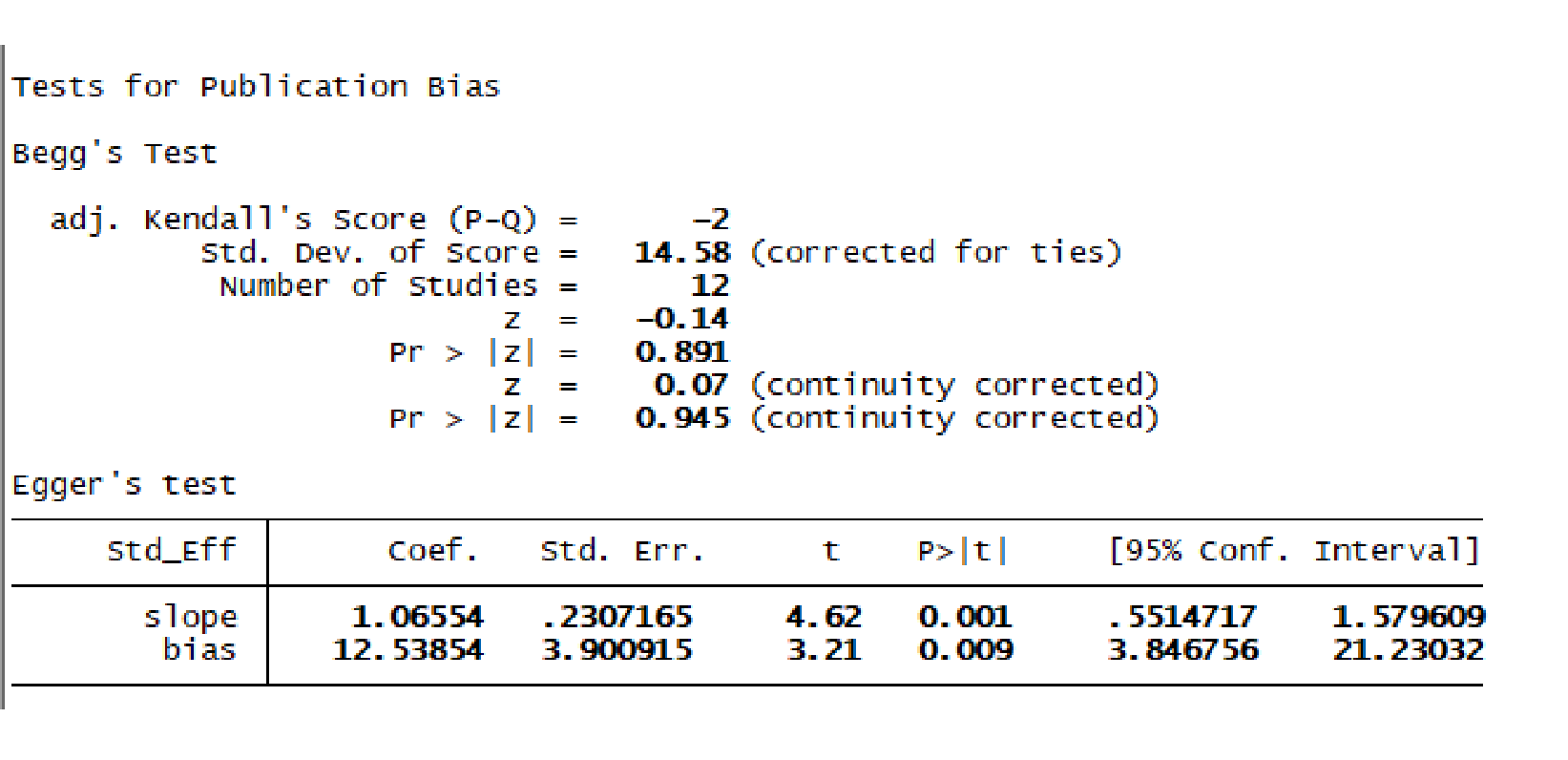

Supplement: S1 Fig — (TIF) [file pgph.0003003.s003.tif]

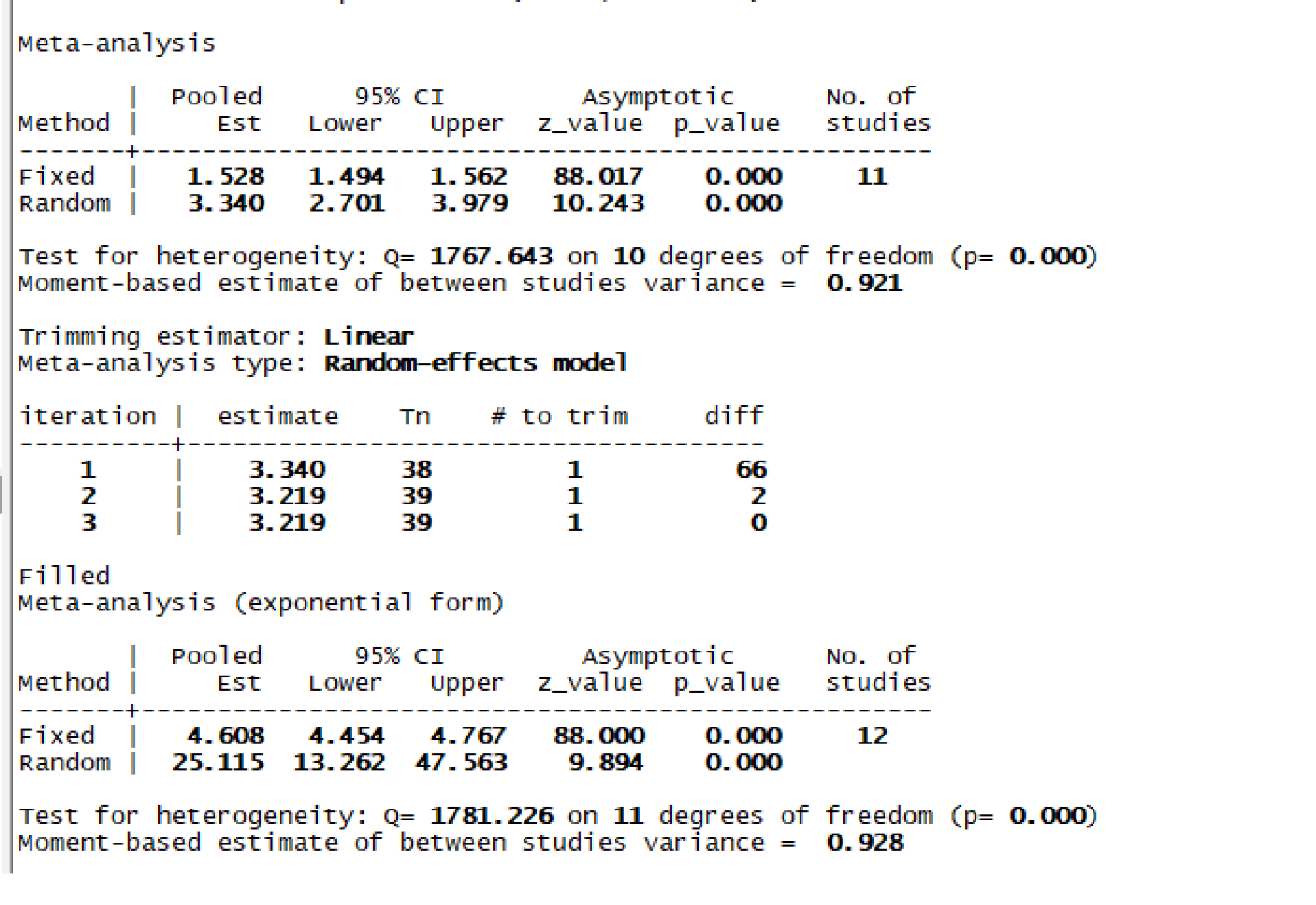

Supplement: S2 Fig — (TIF) [file pgph.0003003.s004.tif]

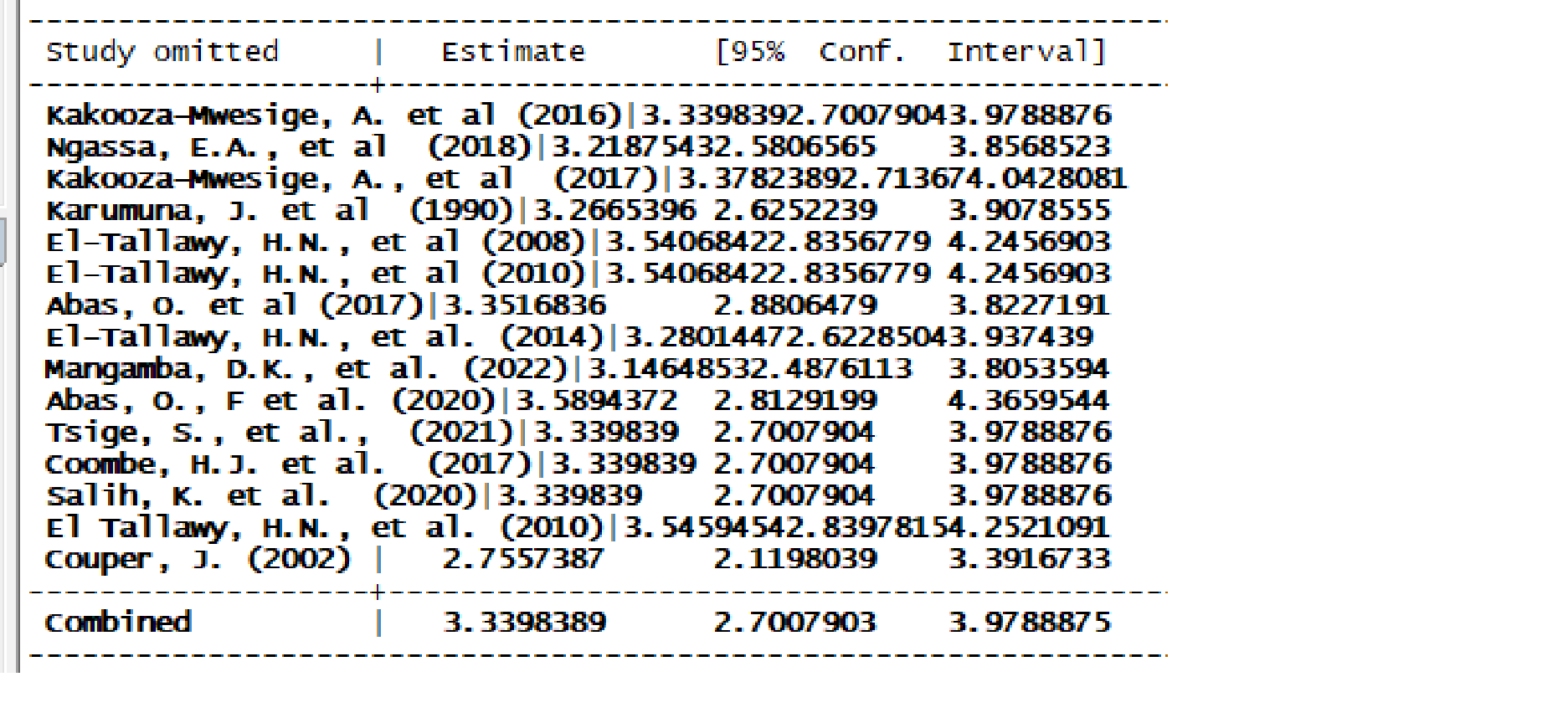

Supplement: S3 Fig — (TIF) [file pgph.0003003.s005.tif]

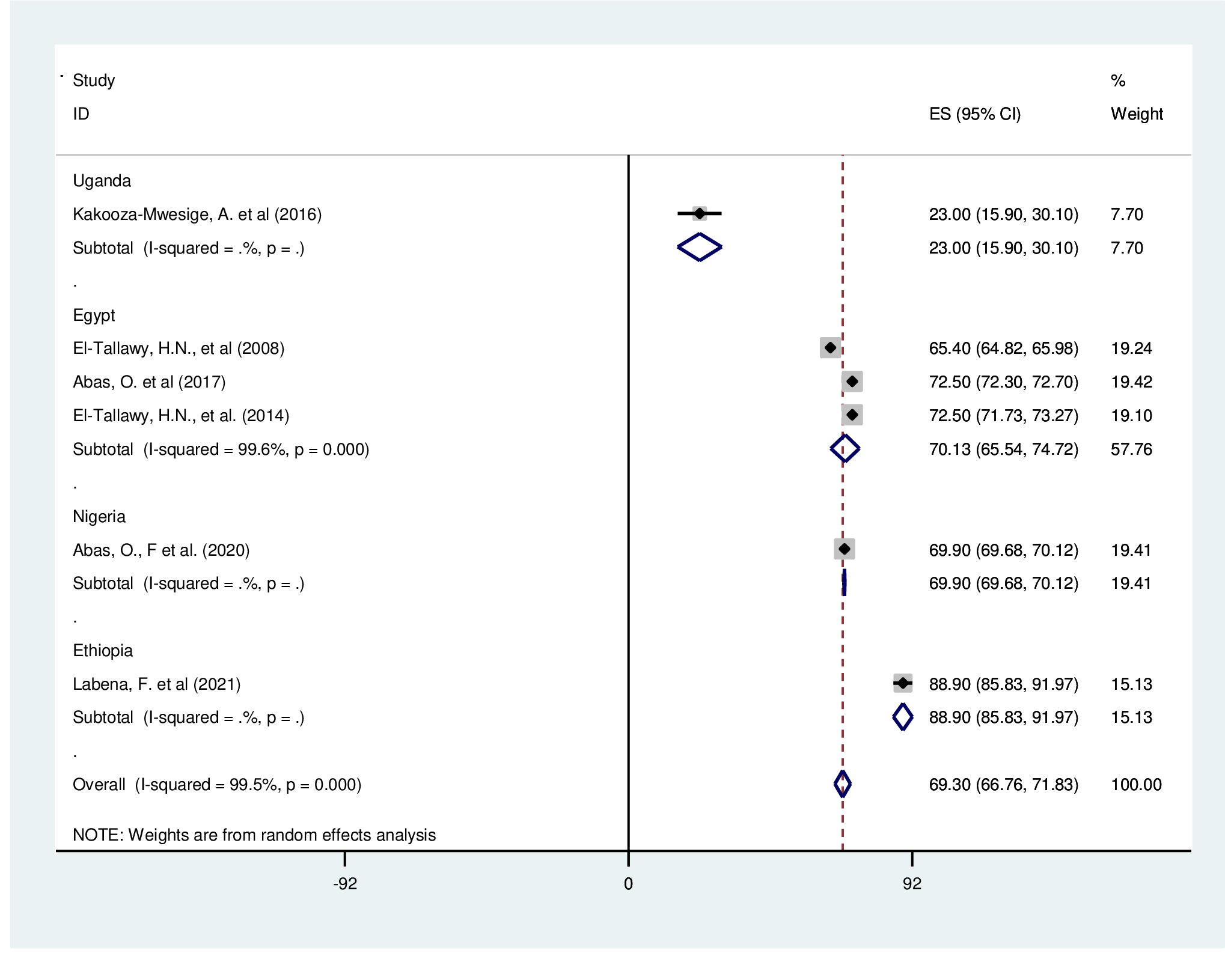

Supplement: S4 Fig — (TIF) [file pgph.0003003.s006.tif]

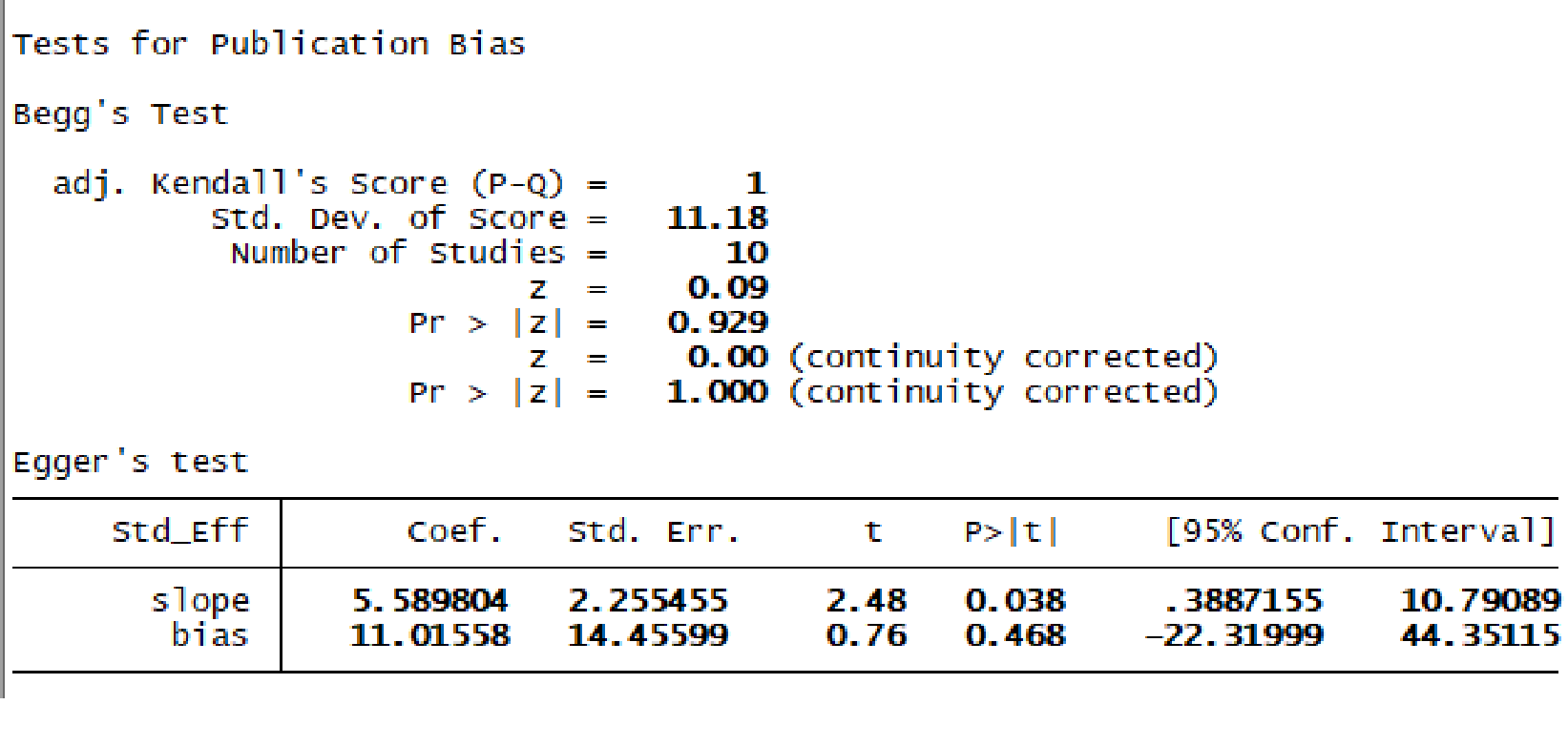

Supplement: S5 Fig — (TIF) [file pgph.0003003.s007.tif]

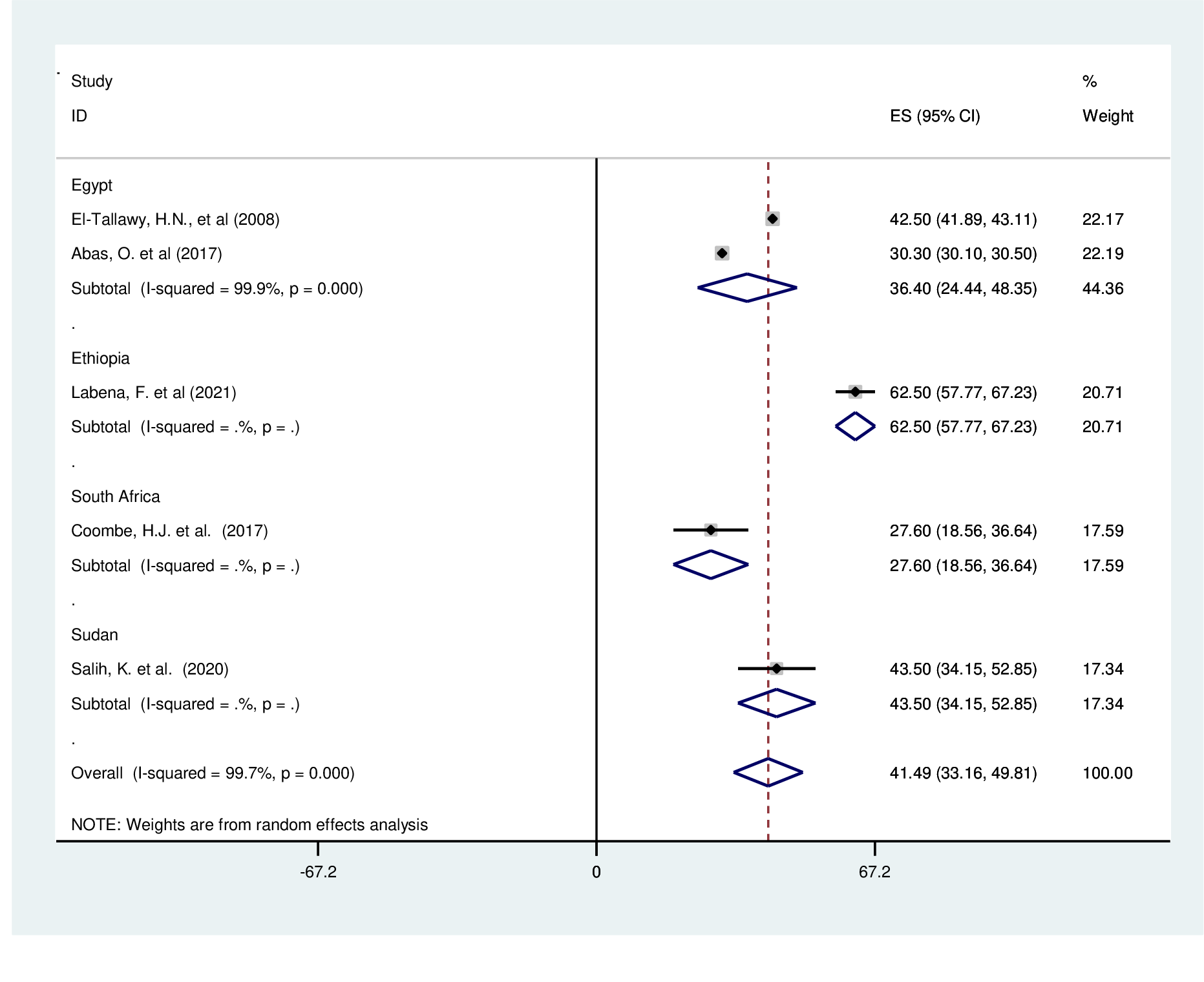

Supplement: S6 Fig — (TIF) [file pgph.0003003.s008.tif]

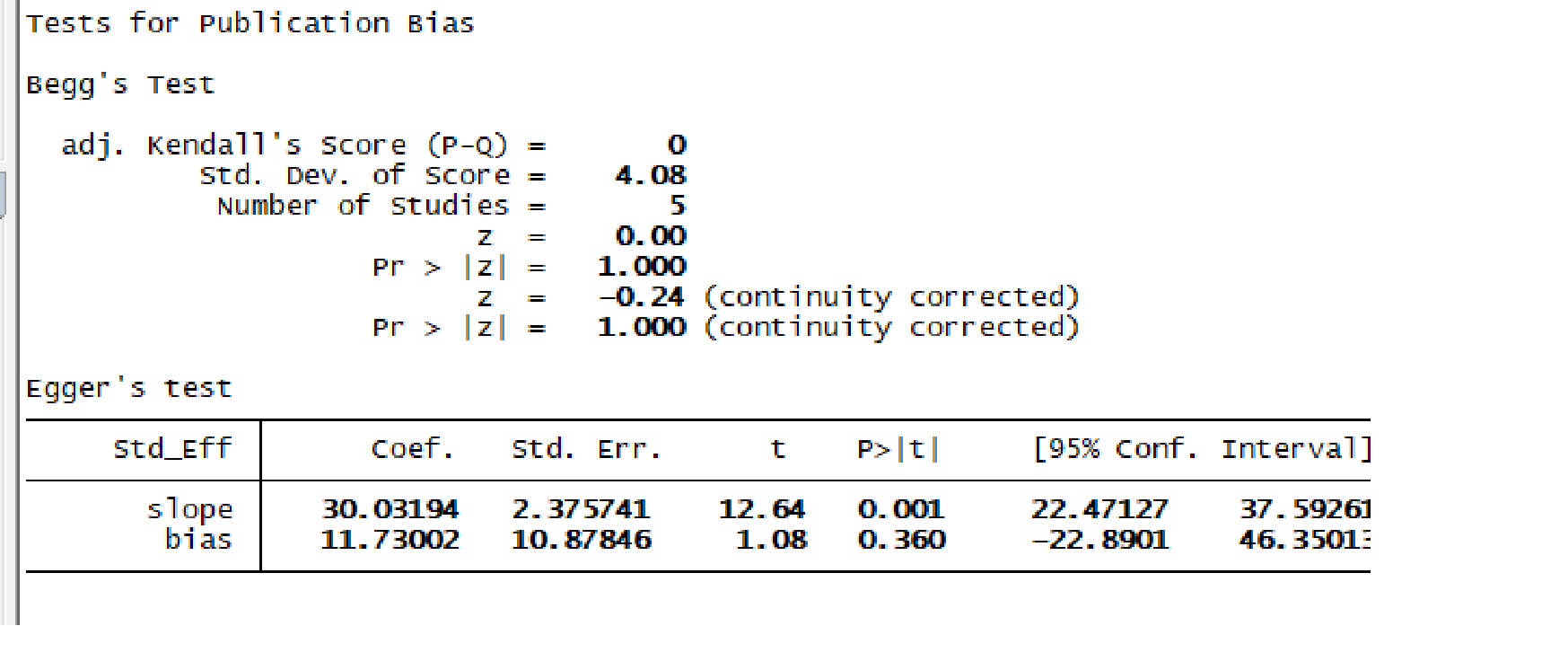

Supplement: S7 Fig — (TIF) [file pgph.0003003.s009.tif]

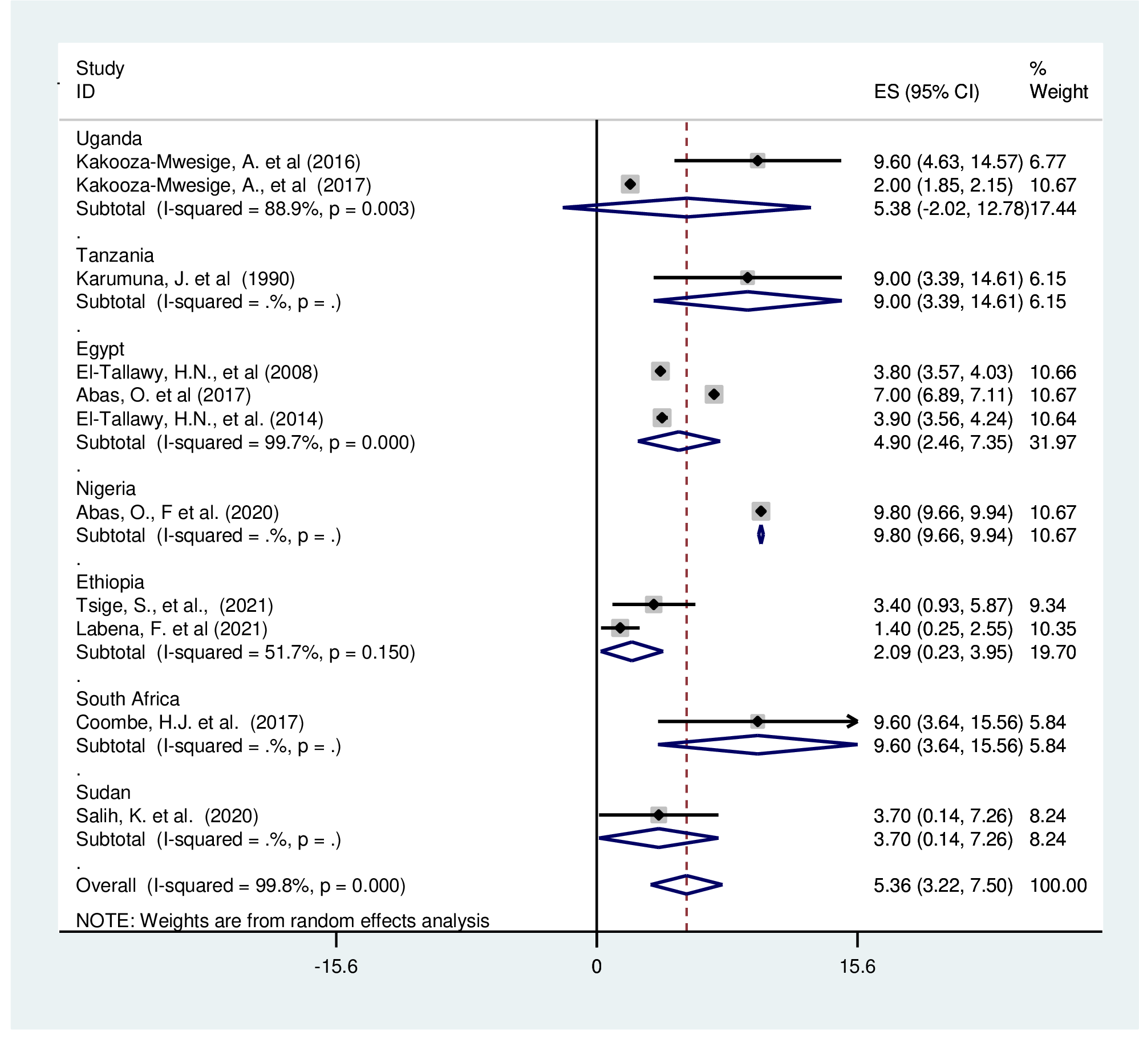

Supplement: S8 Fig — (TIF) [file pgph.0003003.s010.tif]

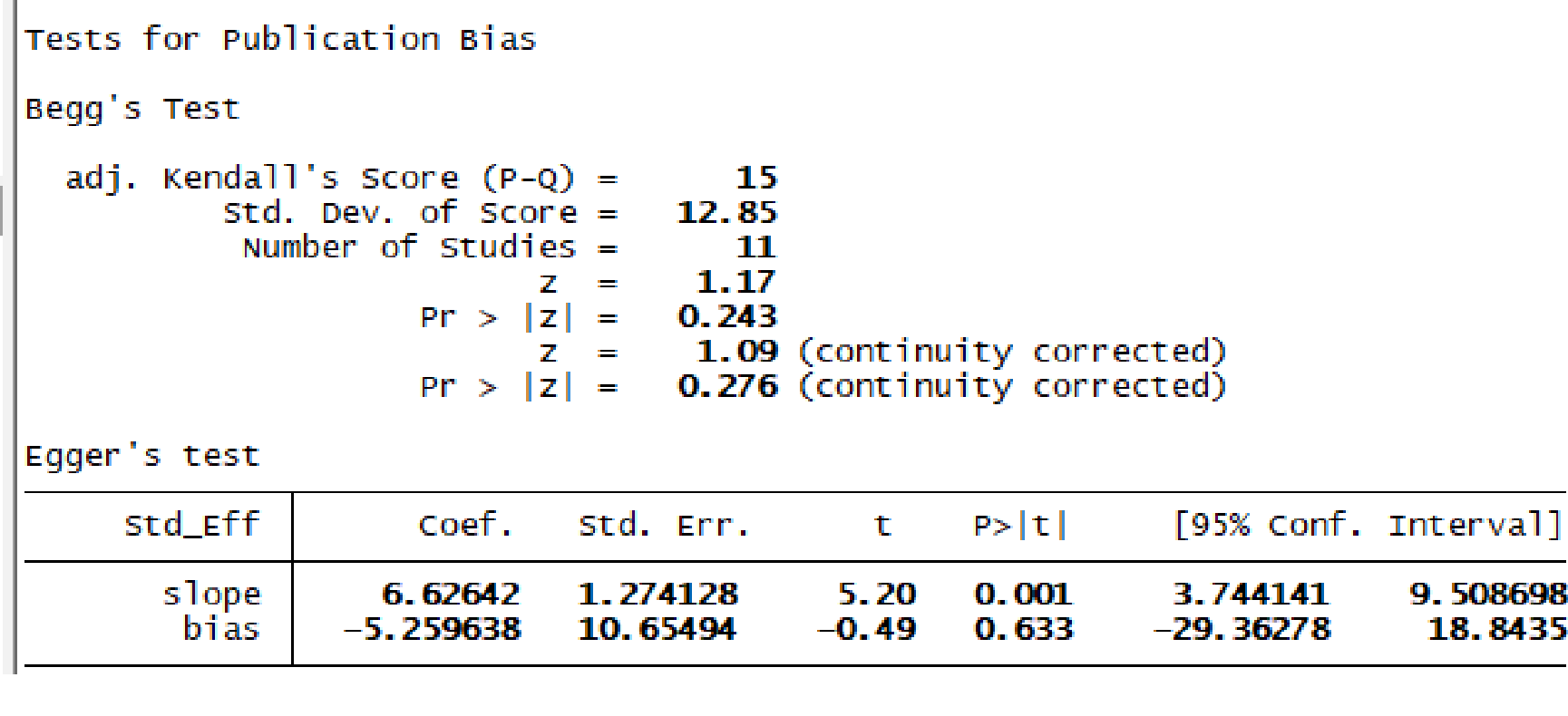

Supplement: S9 Fig — (TIF) [file pgph.0003003.s011.tif]

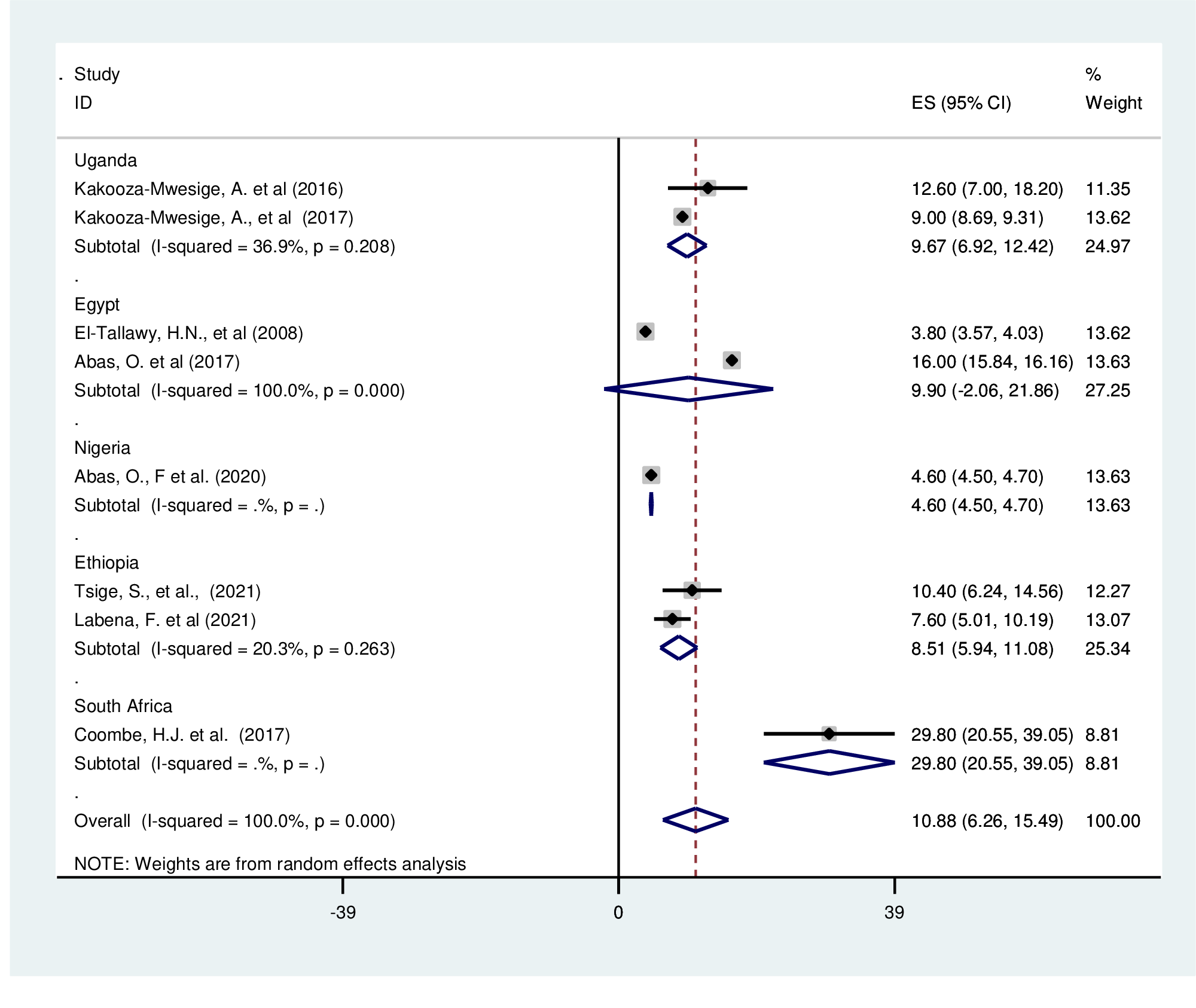

Supplement: S10 Fig — (TIF) [file pgph.0003003.s012.tif]

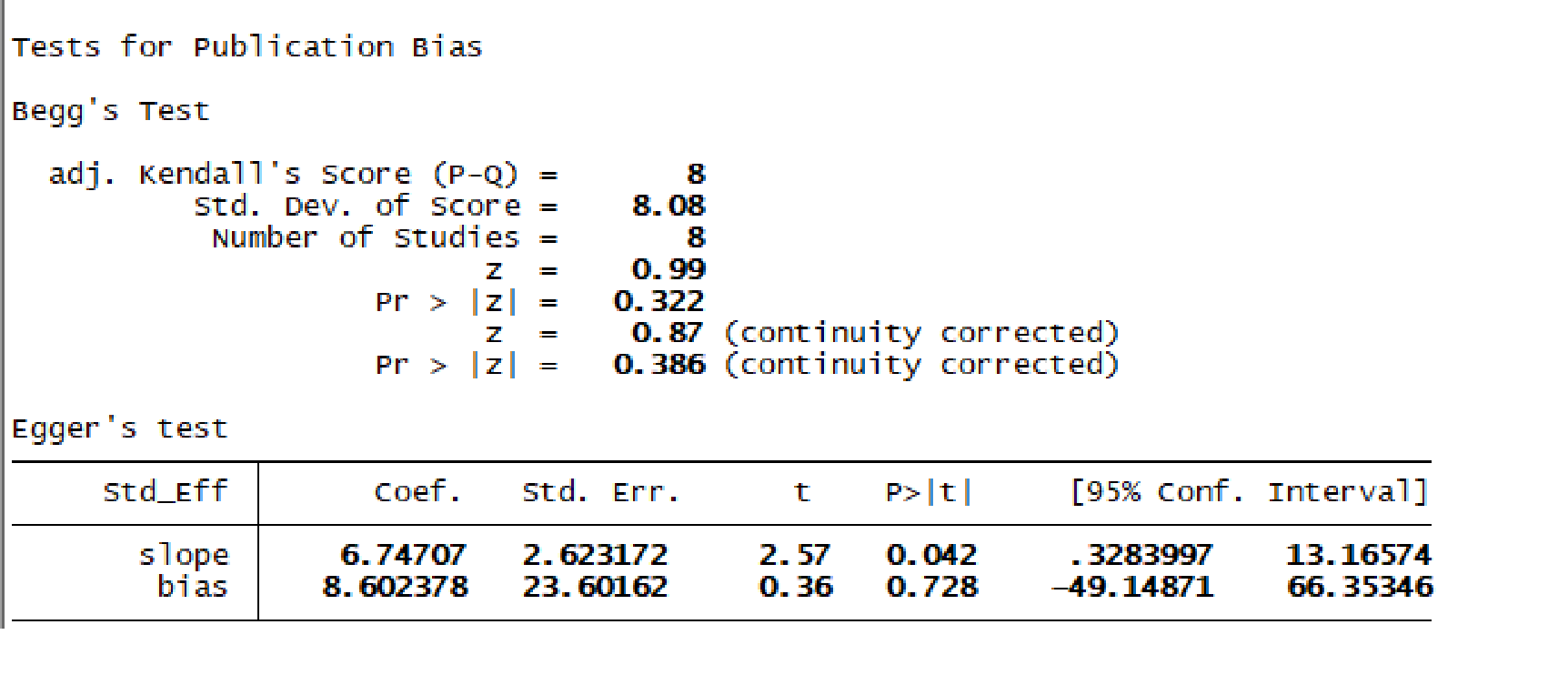

Supplement: S11 Fig — (TIF) [file pgph.0003003.s013.tif]

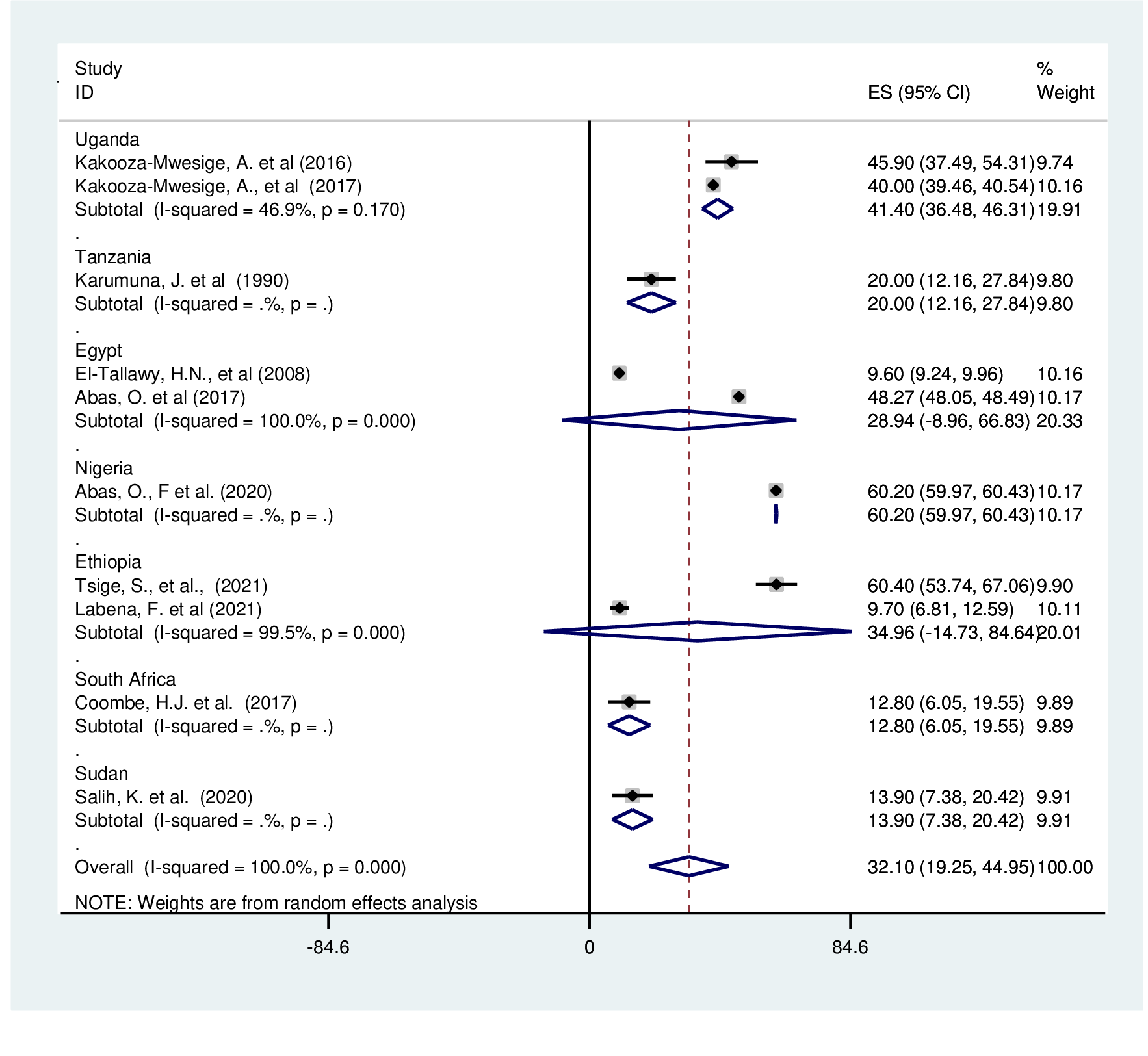

Supplement: S12 Fig — (TIF) [file pgph.0003003.s014.tif]

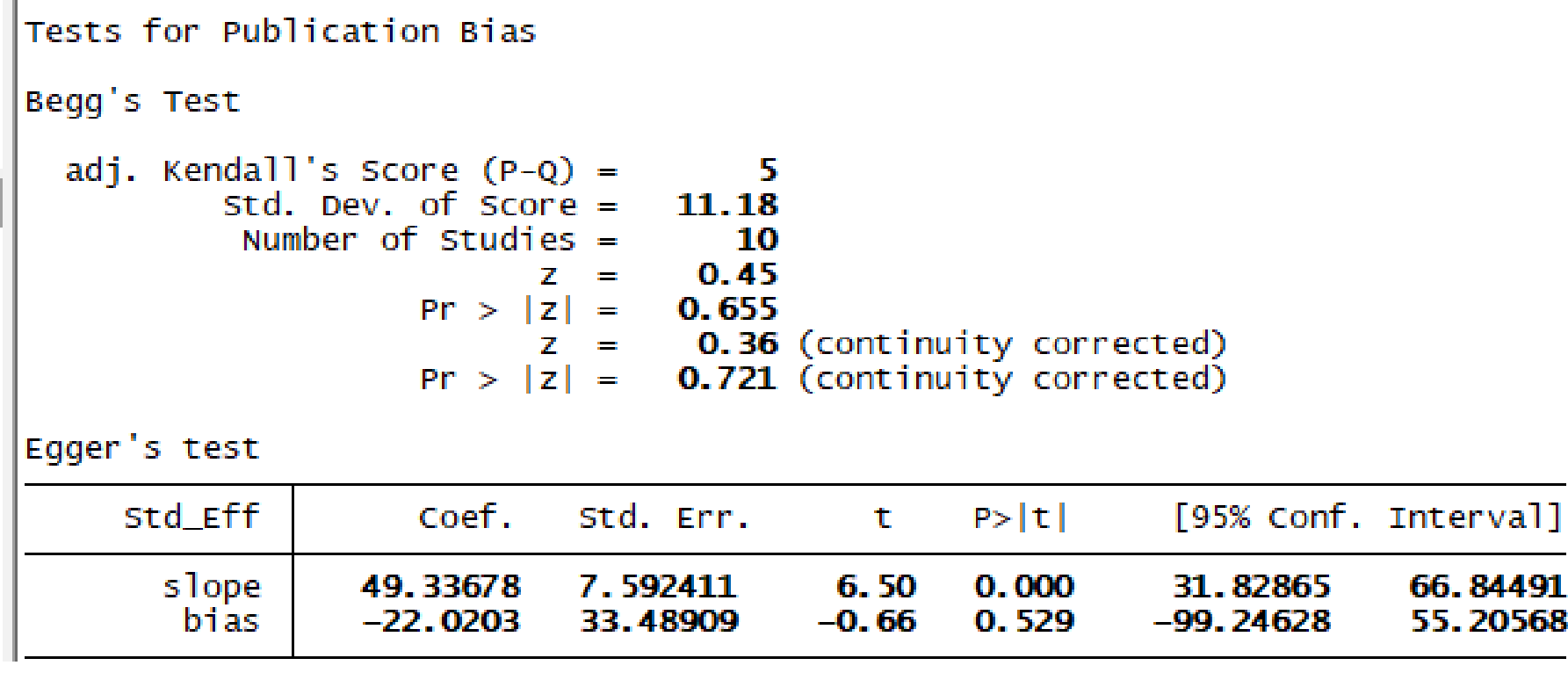

Supplement: S13 Fig — (TIF) [file pgph.0003003.s015.tif]

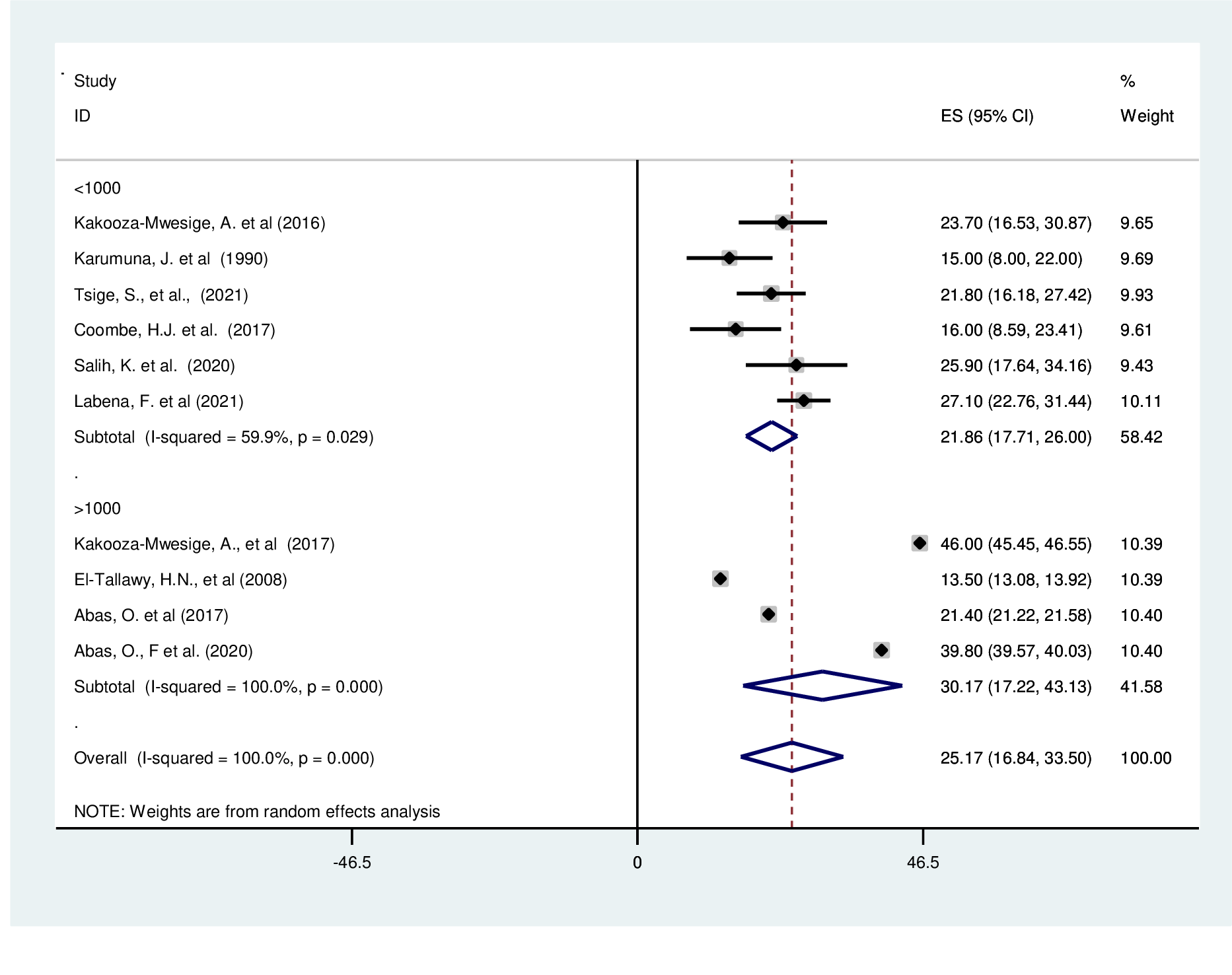

Supplement: S14 Fig — (TIF) [file pgph.0003003.s016.tif]

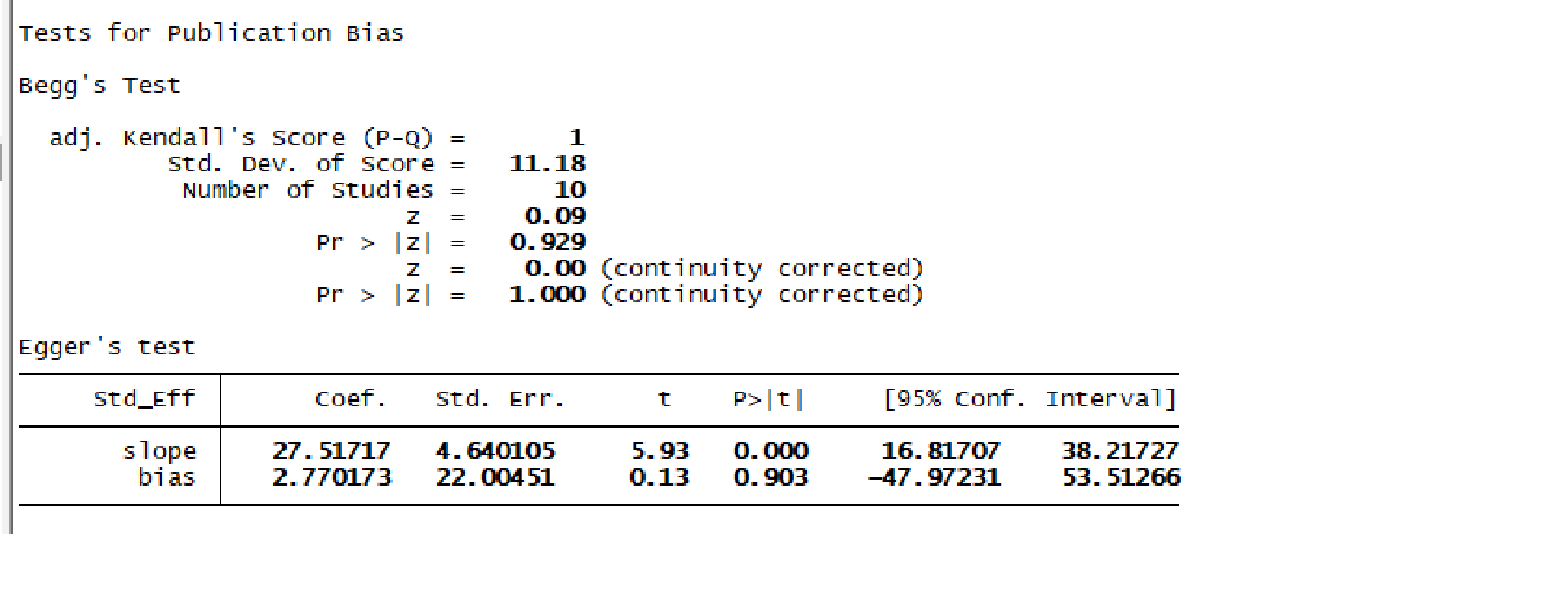

Supplement: S15 Fig — (TIF) [file pgph.0003003.s017.tif]

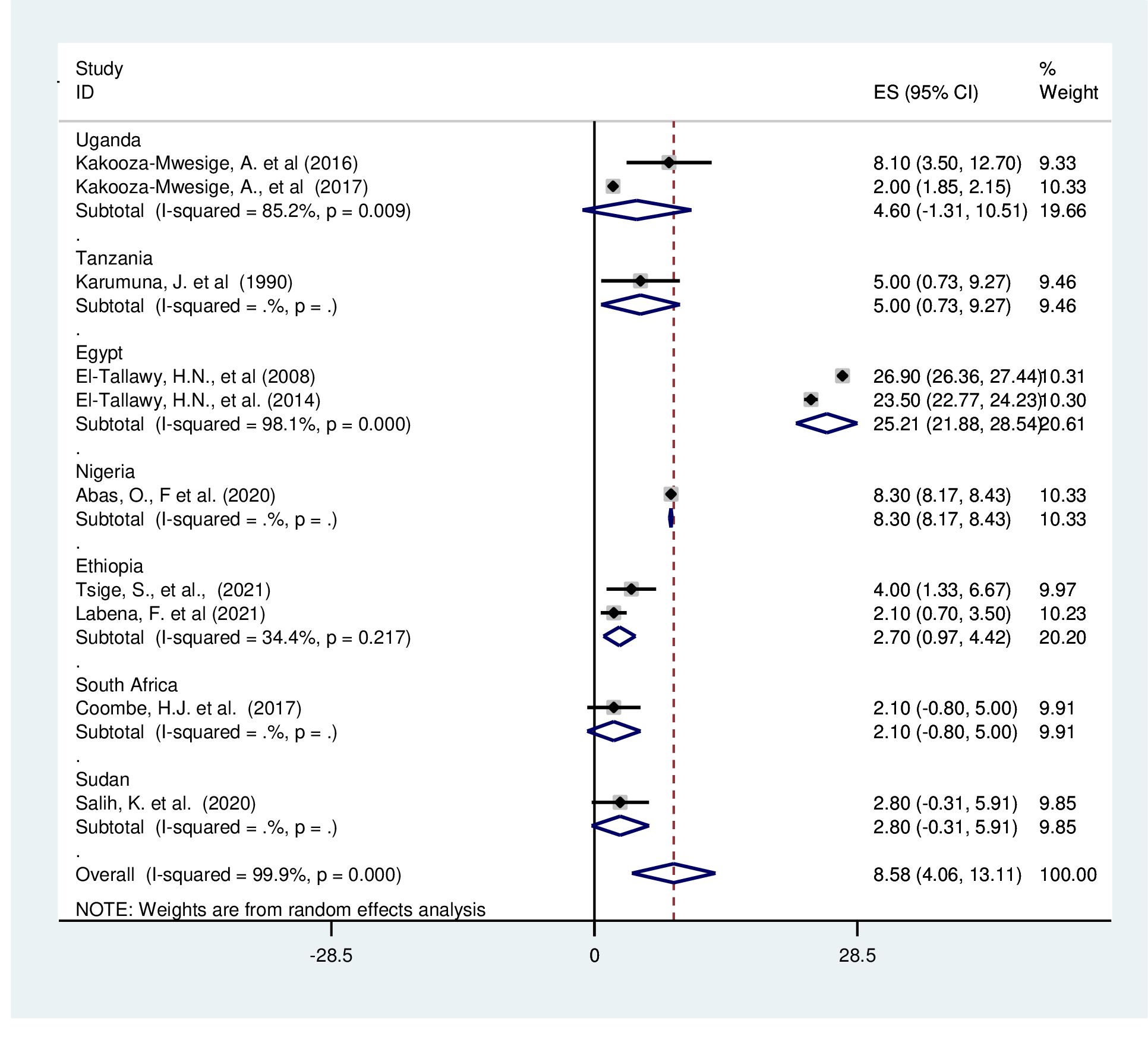

Supplement: S16 Fig — (TIF) [file pgph.0003003.s018.tif]

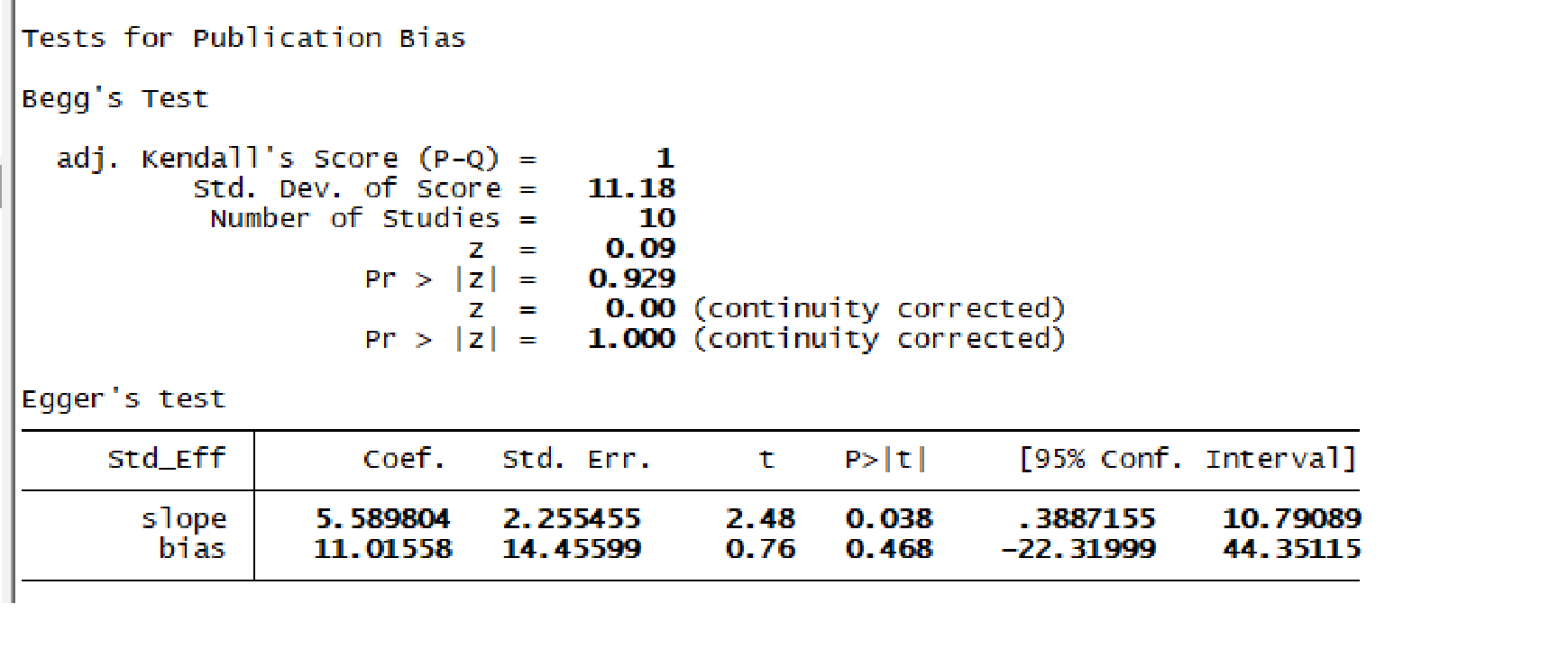

Supplement: S17 Fig — (TIF) [file pgph.0003003.s019.tif]

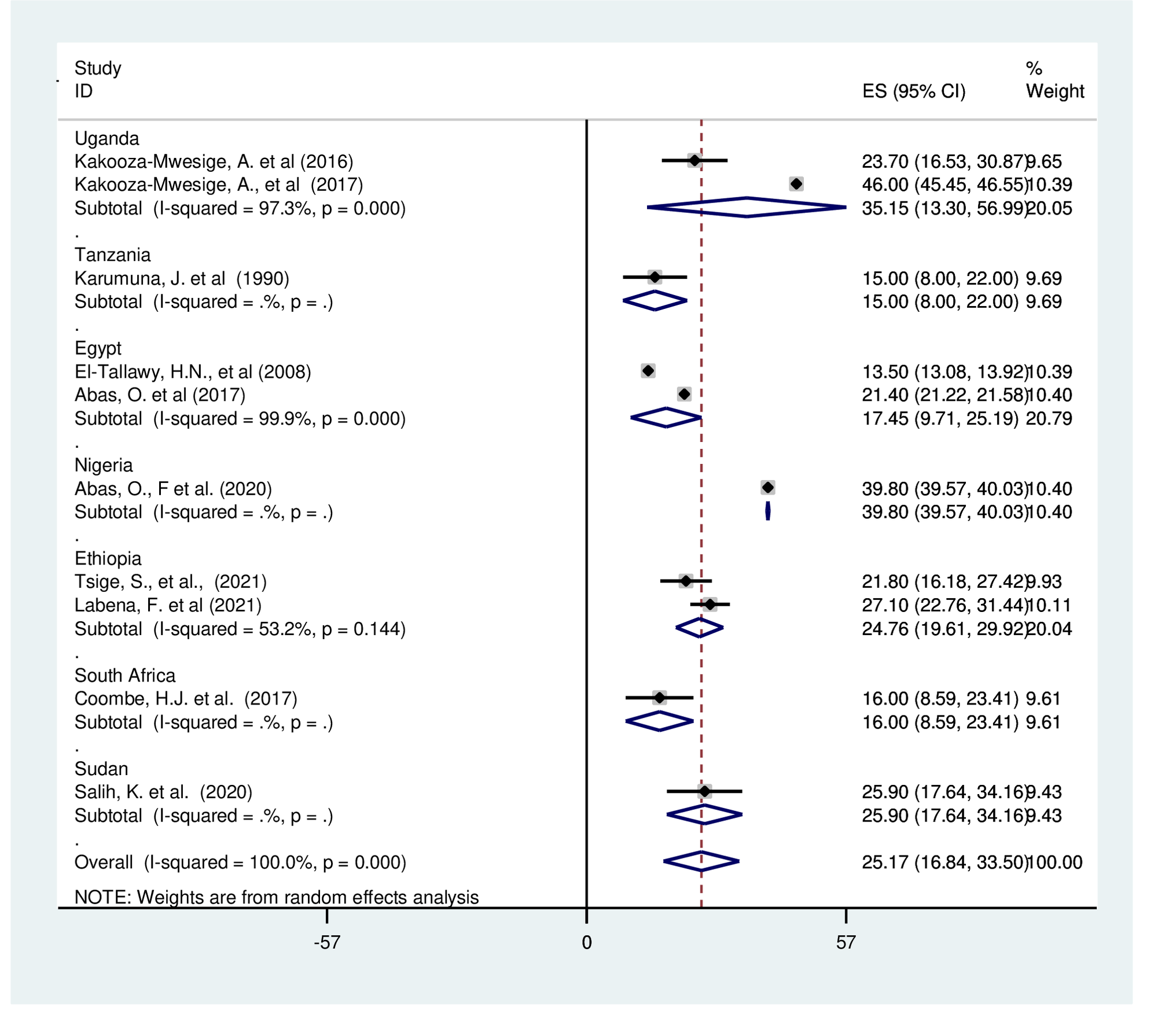

Supplement: S18 Fig — (TIF) [file pgph.0003003.s020.tif]
