# Supplementary material for: Magnitude and clinical characteristics of cerebral palsy among children in Africa: A systematic review and meta-analysis
Source: PLOS Glob Public Health. 2024 Jun 21;4(6):e0003003. doi: 10.1371/journal.pgph.0003003 (PMC11192420; doi:10.1371/journal.pgph.0003003)
Supplement: Attachment — Submitted filename: PGPH-D-23-02244.pdf [file pgph.0003003.s021.pdf]

PLOS Global Public Health

Magnitude and clinical characteristics of cerebral palsy among children in Africa: A systematic review and Meta-analysis

--Manuscript Draft--

**Manuscript Number:**	PGPH-D-23-02244
**Article Type:**	Research Article
**Full Title:**	Magnitude and clinical characteristics of cerebral palsy among children in Africa: A systematic review and Meta-analysis
**Short Title:**	Magnitude and clinical characteristics of cerebral palsy among children in Africa
**Corresponding Author:**	Biruk Beletew Abate<br>Woldia University<br>Woldia, ETHIOPIA
**Order of Authors:**	Biruk Beletew Abate
	Kindie Mekuria Tegegne
	Molla Azmeraw Azmeraw
	Alemu Birara Zemariam
	Addis Wondimagegn
	Gebremeskel Kibret Abebe
**Keywords:**	Keywords: Cerebral palsy, Spastic, Ataxia, Dyskinesia, Africa
**Abstract:**	Background: Cerebral palsy (CP) is the most common motor disability in childhood which causes child's behavioral, feeding, and sleep difficulties.<br>Objectives: The main aim of this study is to assess the pooled prevalence of Cerebral Palsy (CP) and its clinical characteristics in Africa.<br>Design: Systematic review and meta-analysis<br>Methods: Using Preferred Reporting Items for Systematic Review and Meta-Analysis (PRISMA) guidelines to search articles from electronic databases (Cochrane library, Ovid platform (Medline, Embase, and Emcare), Google Scholar, CINAHL, PubMed, Maternity and Infant Care Database (MIDIRS), and institutional repositories in Africa countries. The last search date was on 12/05/ 2023 G. C. The authors extracted the year of publication, country, study design, the prevalence, and types of cerebral palsy. A weighted inverse variance random-effects model was used to estimate the pooled estimates of cerebral palsy and its types. The subgroup analysis was done by country, and sample size. Publication bias and sensitivity analysis were also done.<br>Participants: Studies on prevalence and clinical characteristics of cerebral palsy were included.<br>Primary and secondary outcome measures: The primary and secondary outcome were prevalence and clinical characteristics of cerebral palsy respectively.<br>Results: A total of 15 articles with (n = 498406 patients) were included for the final analysis. The pooled prevalence of cerebral palsy in Africa was found to be 3·34 (95% CI: 2·70, 3·98: I2 = 99·4%; p < 0·001). The most common type is spastic cerebral palsy accounting 69·30% (95% CI: 66·76, 71·83) of all cases. The second one is quadriplegic cerebral palsy which was found to be 41·49% (95% CI: 33·16, 49·81). Ataxic cerebral palsy accounted 5·36% (95% CI: 3·22, 7·50). On the other hand, dyskinetic cerebral palsy was found to be 10.88% (95% CI: 6·26, 15·49). About 32·10% (95% CI: 19·25, 44.95) of cases were bilateral while 25·17% (95% CI: 16·84, 33·50) were unilateral.<br>Conclusions: The overall prevalence of cerebral palsy in Africa found to be high compared to findings from developed countries. spastic, and quadriplegic cerebral palsy were found to be the most common type. Governmental and non-governmental organizations should target their effort on the prevention, early detection and management of cerebral palsy in Africa.
**Additional Information:**	
**Question**	**Response**

Powered by Editorial Manager® and ProduXion Manager® from Aries Systems Corporation

**Financial Disclosure**<br><br>Enter a financial disclosure statement that describes the sources of funding for the work included in this submission and the role the funder(s) played. This includes grants and any commercial funding of the work or authors.<br><br>This statement will be typeset if the manuscript is accepted for publication.<br><br>*Please review the submission guidelines and the instructions link below for detailed requirements and guidance.*	The author(s) received no specific funding for this work.
**Competing Interests**<br><br>On behalf of all authors, disclose any competing interests that could be perceived to bias this work.<br><br>This statement will be typeset if the manuscript is accepted for publication.<br><br>*Please review the instructions link below and PLOS Global Public Health's competing interests policy to determine what information must be disclosed at submission.*	The authors have declared that no competing interests exist.
**Data Availability**<br><br>Before publication, Authors are required to make fully available and without restriction all data underlying their findings. Please see our PLOS Data Policy page for detailed information on this policy.<br><br>A **Data Availability Statement**, detailing where the data can be accessed, is required at first submission. Insert your	All data are available in the manuscript and supporting files.

Data Availability Statement in the box below.<br><br>Please see the data reporting section of our submission guidelines for instructions on what you need to include in your Data Availability Statement.<br><br>This statement will be typeset if the manuscript is accepted for publication.<br><br>*PLOS allows rare exemptions to address legal and ethical concerns. If you have legal or ethical restrictions, please use the box below to detail these in full sentences for the Journal team to consider.*	

Powered by Editorial Manager® and ProduXion Manager® from Aries Systems Corporation

Manuscript

Click here to access/download;Manuscript;Celebral palsy systematic review and meta-analysis.docx

Magnitude and clinical characteristics of cerebral palsy among children in Africa: A systematic review and Meta-analysis

Biruk Beletew Abate[1*], Kindie Mekuria Tegegne[2], Molla Azmeraw Bizuayehu [3], Alemu Birara Zemariam[4], Addis Wondimagegn[5], Gebremeskel Kibret Abebe[6]

Authors Affiliation

[1*]Assistant Professor in Pediatrics and Child Health Nursing, College of Health Science, Woldia University, Ethiopia

[2]MSc in Psychiatry, College of Health Scienc e, Woldia University, Ethiopia

[3]MSc in Pediatrics and Child Health Nursing, College of Health Science, Woldia University, Ethiopia

[4]MSc in Pediatrics and Child Health Nursing, College of Health Science, Woldia University, Ethiopia

[5]MSc in Emergency Medicine and Critical Care Nursing, College of Health Science, Woldia University, Ethiopia

[6]MSc in Emergency Medicine and Critical Care Nursing, College of Health Science, Woldia University, Ethiopia

***Corresponding Author: Biruk Beletew Abate**: Assistant Professor, Woldia University Ethiopia, Email : birukkelemb@gmail.com, Telephone: +251922898070

Abstract

Background: Cerebral palsy (CP) is the most common motor disability in childhood which causes child's behavioral, feeding, and sleep difficulties.

Objectives: ~~The main aim of this study is to~~ assess the pooled prevalence of Cerebral Palsy (CP) and its clinical characteristics in Africa.

Design: Systematic review and meta-analysis

Methods: Using Preferred Reporting Items for Systematic Review and Meta-Analysis (PRISMA) guidelines to search articles from electronic databases (Cochrane library, Ovid platform (Medline, Embase, and Emcare), Google Scholar, CINAHL, PubMed, Maternity and Infant Care Database (MIDIRS), and institutional repositories in Africa countries. The last search date was on 12/05/ 2023 G. C. The authors extracted the year of publication, country, study design, the prevalence, and types of cerebral palsy. A weighted inverse variance random-effects model was used to estimate the pooled estimates of cerebral palsy and its types. The subgroup analysis was done by country, and sample size. Publication bias and sensitivity analysis were also done.

Participants: Studies on prevalence and clinical characteristics of cerebral palsy were included.

Primary and secondary outcome measures: The primary and secondary outcome were prevalence and clinical characteristics of cerebral palsy respectively.

Results: A total of 15 articles with (n = 498406 patients) were included for the final analysis. The pooled prevalence of cerebral palsy in Africa was found to be 3·34 (95% CI: 2·70, 3·98: I^2 = 99·4%; p < 0·001). The most common type is spastic cerebral palsy accounting 69·30% (95% CI: 66·76, 71·83) of all cases. The second one is quadriplegic cerebral palsy which was found to be 41·49% (95% CI: 33·16, 49·81). Ataxic cerebral palsy accounted 5·36% (95% CI: 3·22, 7·50). On the other hand, dyskinetic cerebral palsy was found to be 10.88% (95% CI: 6·26, 15·49). About 32·10% (95% CI: 19·25, 44.95) of cases were bilateral while 25·17% (95% CI: 16·84, 33·50) were unilateral.

Conclusions: ~~The overall prevalence of cerebral palsy in Africa found to be high compared to findings from developed countries. spastic, and quadriplegic cerebral palsy were found to be the most common type. Governmental and non-governmental organizations should target their effort on the prevention, early detection and management of cerebral palsy in Africa.~~

Keywords: *Cerebral palsy*, *Spastic*, *Ataxia*, *Dyskinesia*, *Africa*

Strengths and limitations of this study

► The study has used a prespecified protocol for search strategy and data abstraction

► We used internationally accepted tools for a critical appraisal system for quality assessment of individual studies.

► It is difficult to determine if the results from various regions are representative of the entire country, as no data were found for all regions of the country.

Background

Cerebral palsy (CP) is a group of permanent disorders of development that affect different body parts and result in activity limitations with the manner of walking, muscle tone, posture, and coordination of movement. This is attributed to a non-progressive disturbance that occurs in the development of the fetal or infant brain.(1) Cerebral palsy contains different types that are characterized by the location of the brain part damaged. This happens when messages from the brain do not properly reach the muscle. Even though, many studies reveal that CP arises from many different causes including premature birth, medical problems and illness or injuries, the pathophysiology of this problem is not clear.(2).Cerebral palsy movement abnormalities are frequently accompanied with sensory, perception, cognition, communication, and behavior issues, seizures, and subsequent musculoskeletal problems.(3) Children with CP are present with certain characteristics which include spasticity, hypotonia, diplegia, hemiplegia, and dystonia.(4) Usually, motor disorders are more common and accompanied by perception cognition, sensation, communication and behavior due to epilepsy and secondary musculoskeletal problems.(1) CP greatly affects the childhood characteristics such as behavioral difficulties, feeding difficulties, social skills, and sleep.(5)

Cerebral palsy (CP) is the most common neurologic problem causing motor disability in children.(6) According to the World Health Organization report globally, there were 10% of children (approximately 200 million) suffer from physical disability, mental deficiencies or developmental delay, and impaired learning abilities.(7) Furthermore, more than three fourth of the world's disabled population live in low-income countries, many of these in Africa.(8) Recent population-based studies from around the world reported that the prevalence of CP was ranging from 1 to nearly 4 per 1,000 live births.(4) The prevalence of CP is variably reported across different countries which

are 1.89 per 1,000 live births in Norway,[9] and 2.2 per 1,000 live births in Denmark.[10] Furthermore, a population-based study in Bangladesh reported that 3.4 per 1,000 children.[11]

In Africa, the prevalence of CP is also ~~high~~ compared to developed nations. Evidently, studies on hospital clinical samples suggest prevalence ranging from 2 to 10 cases per 1000 live births from Egypt, Uganda, South Africa, and South Egypt.[12] Moreover, a population-based study in Uganda revealed that the prevalence of CP was 2.9 per 1,000 children in 2017.[13] In line with the global trend, CP places a heavy burden of disease on children, families, and society in both developed and developing countries.[4,10] The previous studies done in different countries showed that several factors were associated with CP. Among these factors, asphyxia at birth, low birth weight, intrauterine infections and multiple gestations were the most important determinants for CP.[14] Cases of CP varies in presentation, etiology, evolution, severity, medical and rehabilitation needs, comorbidities, and outcomes.[15] Moreover, children with cerebral palsy (CP) were significantly less common at older ages than at younger ones, and the prevalence also decreased in line with this trend. These findings suggested that there was a high mortality rate among children with cerebral palsy, particularly among those who were most severely affected. The dearth of knowledge regarding the epidemiology of CP in LMICs emphasizes the abuse and lack of treatment and support for children with CP in these nations which in turn reduces their consecutive birth date survival.[13] It might be appreciated at early or later age. Cerebral palsy was diagnosed in 43.4% of children after the first year of life, 32.4% after the second half of life, and 24.1% before the age of six months.[16]This figure shows there is time delay in the diagnosis of CP.

Globally, several strategies and interventions have been tried to reduce the prevalence and adverse sequels of CP among children.[8] These include electronic fetal monitoring, cesarean section, generally enhancing obstetric and neonatal care as it was expected that improvement in these areas would result in lower rates of cerebral palsy since prenatal events are thought to be responsible for approximately 75% of all cases of cerebral palsy.[4,7,17] Beyond these efforts, the problem has still a public concern, particularly in developing nations,[18] because their obstetric and neonatal advanced medical care is limited.[19] Few rigorous population-based studies have recently been published from Uganda,[13] and Bangladesh[11] revealing large discrepancy in prevalence between them and studies from high income countries. Studies published from African countries revealed large discrepancy in prevalence of CP and from studies in high income countries. These studies clearly indicated that the data from these scattered evidence or from studies in high income countries and recommended the need of comprehensive review on CP from low and middle income countries.[20] There is a lack of structured and consistent screening policy for developmental disabilities amongst children in Africa.[21] This situation coupled with delayed presentation has left many children with disabilities unrecognized and hence without appropriate intervention. This in turn underestimate the magnitude and burden of CP in Africa. Children with disabilities and their families in African countries are frequently excluded from society because of stigmatization. Most of these children consequently confront many challenges socially, economically, and politically because they are often denied the basics of health care, education, socialization, and recognition.[22] To date, a rigorous systematic review and meta-analysis of the overall prevalence of CP are lacking in resource-poor settings despite the fact that large burden of the disease. Hence, this study is intended to assess the pooled prevalence of CP and its clinical characteristics among children in Africa.

Methods

Search strategy

This systematic review and meta-analysis review assessed studies that provide data on the magnitude and clinical characteristics of cerebral palsy in the Africa context. We searched these articles from the following databases: Cochrane library, Ovid platform (Medline, Embase, and Emcare), Google Scholar, CINAHL, PubMed, Maternity and Infant Care Database (MIDIRS), and institutional repositories in Africa countries on 12/05/ 2023 G. C. The search in all database included keywords that are the combinations of population, condition/outcome, and context. A snowball searching for the references of relevant papers for linked articles was also performed.

The following search map was applied: (prevalence OR magnitude) AND (Children [MeSH Terms] OR infant OR child OR childhood) AND (cerebral palsy [MeSH Terms] OR "developmental disabilities", OR "neurological impairment," OR "childhood disability") AND (ataxia OR dyskinesia OR spastic) AND ("clinical characteristics [MeSH Terms]" OR type) AND (Africa OR "developing countries") on PubMed database (Table S1). Thus, the PubMed search combines #1 AND #2 AND #3 AND #4 AND #5 (Table S1). These search terms were further paired with the names of African countries. Thus, the key searching terms were considering Africa countries that compose of Ethiopia, Djibouti, Somalia, Egypt, Eritrea, Sudan, Tanzania, Kenya, Nigeria, Uganda etc. Using those key terms, we used the Boolean operator "OR" (to connect key terms/phrases within the same concept), "AND" (to connect key terms /phrases between two concepts), and "NOR" to filter out. In addition, we used truncation (*), adjacency searching (**ADJn)**, and wildcard symbols to find

variations in spelling and variant word endings on the Ovid databases. Moreover, we applied relevant limits (filters) such as a limit to human studies only. The sample search strategy for Medline is attached in the appendix section of this manuscript.

Study selection and screening

The retrieved studies were exported to Endnote version 8 reference managers to remove duplicate studies. Two investigators (BB and KM) independently screened the selected studies using article's title and abstracts before retrieval of full-text papers. We used pre-specified inclusion criteria to further screen the full-text articles. Disagreements were discussed during a consensus meeting with other reviewers (AB and AW) for the fin selection of studies to be included in the systematic review and meta-analysis. The retrieved studies were imported in covidence platform to remove duplicate studies and to do the whole screening process.

Inclusion and exclusion criteria

Studies that assess the magnitude and clinical characteristics of cerebral palsy among children under the age of 18 were considered. Citations without abstract and/or full text, anonymous reports, editorials, and qualitative studies were excluded from the analysis. The Prevalence of cerebral palsy was considered as the proportion of children with cerebral palsy among 1000 risk population.

Quality assessment

The authors appraised the quality of the studies by using the Joanna Briggs Institute (JBI) quality appraisal checklist.[(23)] There was a team of four reviewers and the papers were split amongst the team. Each paper was then assessed by two reviewers and any disagreements were discussed with the third and the fourth reviewers. Studies were considered as low risk or good quality when it scored

4 and above for all designs (cross-sectional, and cohort) [19], whereas the studies scored 3 and below were considered as high risk or poor quality and excluded (Table S2). Furthermore, we thoroughly extract adjusted confounders and main findings from all included studies (Table S3).

Data extraction

The authors developed a data extraction form on the excel sheet and the following data were extracted for eligible studies: year of publication, country, study design, the definition of cerebral palsy, and clinical characteristics/ type of cerebral palsy. The data extraction sheet was piloted using 4 papers randomly, and it was adjusted after piloted the template. Two of the authors (BB and KM) extracted the data using the extraction form in collaboration. The third and fourth (MA and AB) authors checked the correctness of the data independently. Any disagreements between reviewers were resolved through discussions with third and fourth reviewers when required. The mistyping of data was resolved through crosschecking with the included papers.

Synthesis of results

The authors transformed the data to STATA 17 for analysis after it was extracted in an excel sheet considering prevalence, and type/characteristics reported. We pooled the overall prevalence estimates of cerebral palsy by a random effect meta-analysis model. We examined the heterogeneity of effect size using the Q statistic and the I^2 statistics. In this study, the I^2 statistic value of zero indicates true homogeneity, whereas the value 25%, 50%, and 75% represented low, moderate and high heterogeneity, respectively. Subgroup

analysis was done by the study country, study design, and year of publication. Sensitivity analysis was employed to examine the effect of a single study on the overall estimation. Publication bias was checked by the funnel plot and more objectively through Egger's regression test.

Results

A total of 5394 studies were identified; 5380 from different databases and 14 from other sources. After duplication removed, a total of 2,431 articles remained (2963 removed by duplication). Finally, 206 studies were screened for full-text review, and 15 articles with (n = 498406 patients) were included for the final analysis (Fig. 1, and Table S2).

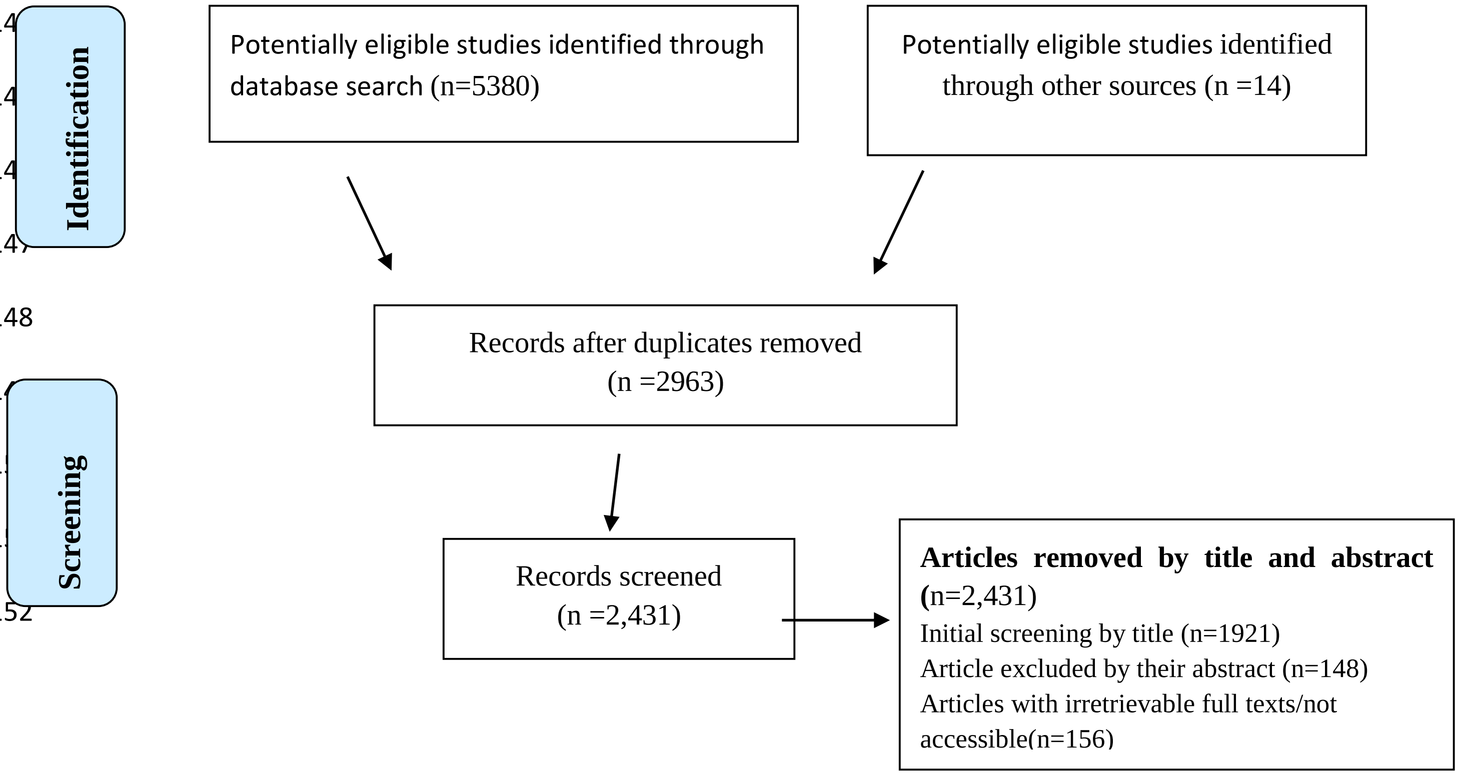

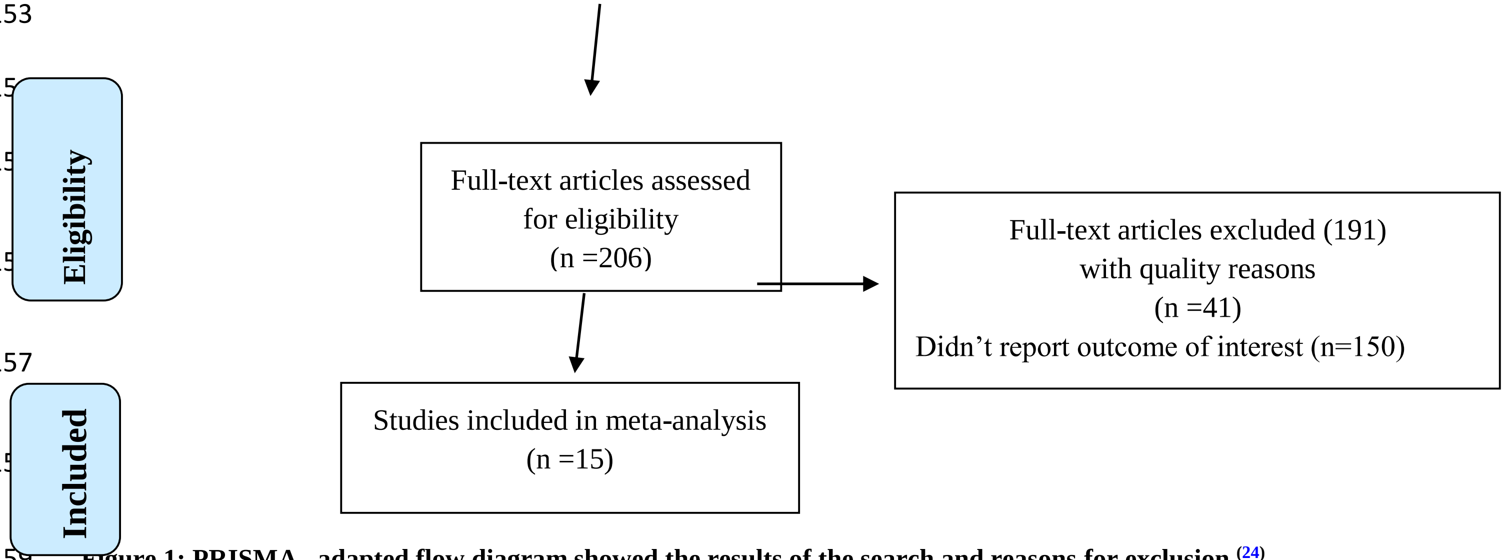

Figure 1: PRISMA –adapted flow diagram showed the results of the search and reasons for exclusion.[24]

Characteristics of included studies

Table 1: summarizes the characteristics of the 15 included studies in this systematic review and meta-analysis [12b,12d,13,25]. Two studies were from Ethiopia,[25a,25f] two from Uganda,[13,25b] two from Tanzania,[25c,25d] three from Egypt,[12d,25e,25i] one from Cameroon,[25j] one from Nigeria,[25k] two from South Africa,[12b,25g] and one from Sudan.[25h] (Table 1)

Table 1: Distribution of included studies on the prevalence and characteristics of cerebral palsy in Africa, 2023

Sr No	Authors	Year	Country	Study design	Sample size	Prevalence (%)	Characteristics/types of cerebral palsy						
							Spastic	Quadriplegic	Ataxic	Dyskinestic	Bilateral	Unilateral	Mixed

**1.**	Labena, F. et al[25a]	2021	Ethiopia	Cross-sectional	402	..	88.9	62.5	1.4	7.6	9.7	27.1	2.1
**2.**	Kakooza-Mwesige, A. et al[25b]	2016	Uganda	Cross-sectional	135	..	23		9.6	12.6	45.9	23.7	8.1
**3.**	Ngassa, E.A., et al [25c]	2018	Tanzania	Cross-sectional	120	17.77	..	..	..	..	..	..	..
**4.**	Kakooza-Mwesige, A., et al [13]	2017	Uganda	Cross-sectional	31756	2.9	..	..	2	9	40	46	2
**5.**	Karumuna, J. et al[25d]	1990	Tanzania	Cohort	100	10	..	..	9	..	20	15	5
**6.**	El-Tallawy, HN., et al [25e]	2008	Egypt	Cross-sectional	25540	2.03	65.4	42.5	3.8	3.8	9.6	13.5	26.9
**7.**	El-Tallawy, H.N., et al [25i]	2010	Egypt	Cross-sectional	25540	2.03	..	..	..	..	..	..	..
**8.**	Abas, O. et al[12d]	2017	Egypt	Cross-sectional	198776	1	72.5	30.3	7	16	48.27	21.4	
**9.**	El-Tallawy, H.N., et al[25e]	2014	Egypt	Cross-sectional	12788	3.6	72.5		3.9	..	..	..	23.5
**10.**	Mangamba, D.K., et al [25j]	2022	Cameroon	Cross-sectional	4064	4.86	..	..	..	..	..	..	..
**11.**	R Duke, et al.[25k]	2020	Nigeria	Cross-sectional	171200	2.3	69.9	..	9.8	4.6	60.2	39.8	8.3
**12.**	Tsige, S., et al.[25f]	2021	Ethiopia	Cross-sectional	207	..	..	..	3.4	10.4	60.4	21.8	4
**13.**	Coombe, H.J. et al. [25g]	2017	South Africa	Cross-sectional	94	..	..	27.6	9.6	29.8	12.8	16	2.1

**14.**	Salih, K. et al. [25h]	2020	Sudan	Cohort	108	..	..	43.5	3.7		13.9	25.9	2.8
**15.**	Couper, J.[12b]	2002	South Africa	Cross-sectional	2 036	10	..	..	..	..	..	..	..

Prevalence of cerebral palsy in Africa

Most of the included studies (n = 11) have reported the prevalence of cerebral palsy per 1000 live births.[12b,12d,13,25d,25e,25i-k] The prevalence of cerebral palsy was ranged from 1(95% CI: 0·96, 1·04) to 17·77 (95% CI: 10·93, 24·61). The random-effects model analysis from those studies revealed that, the pooled prevalence of cerebral palsy in Africa was found to be 3·34 (95% CI: 2·70, 3·98) (95% CI: $I^2 = 99{\cdot}4\%$; $p < 0{\cdot}001$) (Fig. 2).

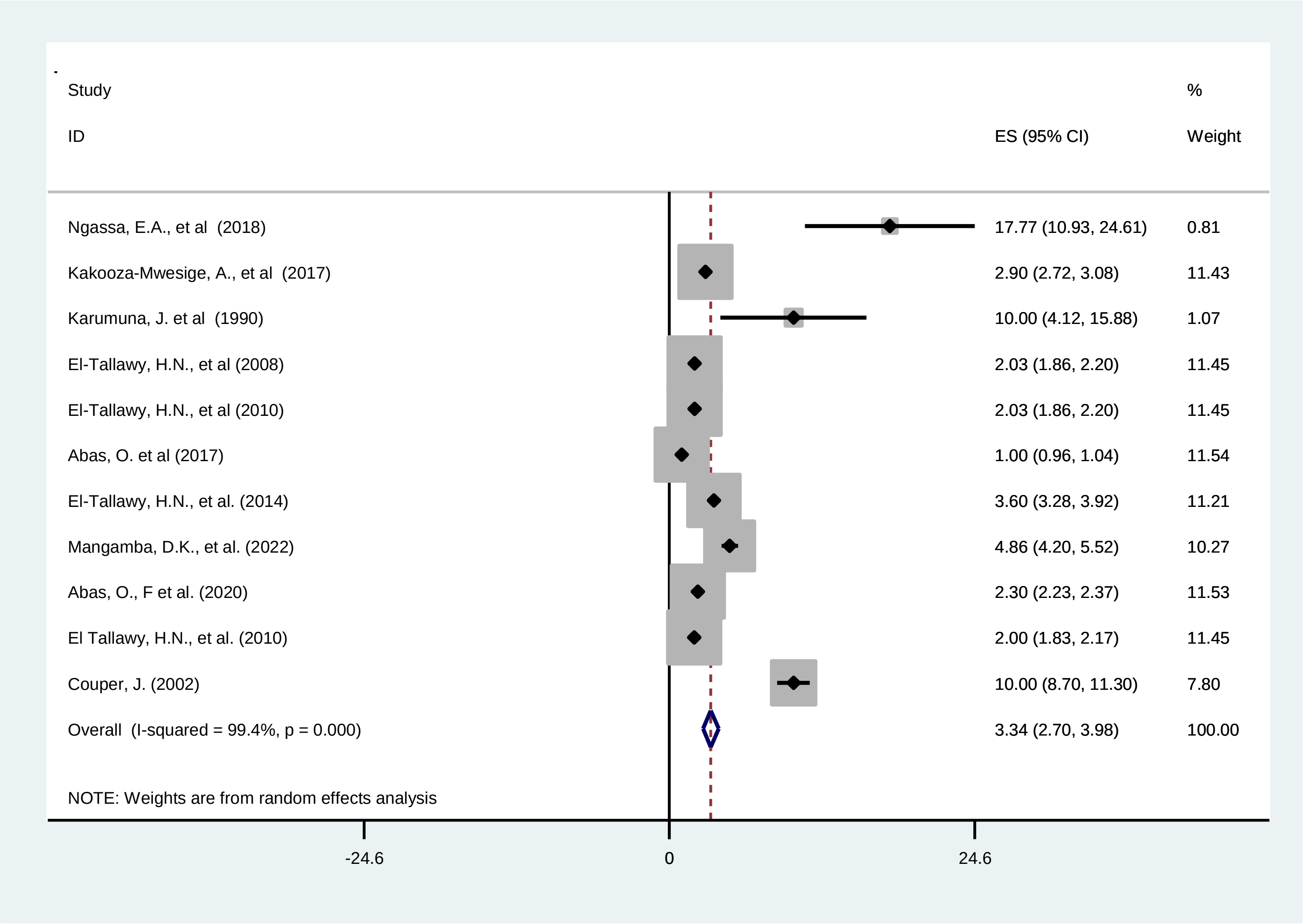

Figure 2: Forest plot showing the pooled prevalence of cerebral palsy among children in Africa

Subgroup analysis: Subgroup analysis was done through stratified by country, and sample size. Based on this, the prevalence of cerebral palsy was found to be 2·90(2·72-3·08) in Uganda, 13·68(6·08-21·28) in Tanzania, 2·12 (1·37, 2·88) in Egypt, 4·86 (4·20, 5·52), 2·30 (2·23, 2·37) in Nigeria and 10·00 (8·70,11·30) in South Africa (Fig. 3). In terms of sample size, the prevalence of cerebral palsy was found to be 3·12 in studies with sample size more 1000 and 13·68 in those with sample size less than 10000 (Supplementary Fig. 1).

Publication bias: A funnel plot showed asymmetrical distribution. The Egger's regression test-value was 0·009, which indicated that, the presence of publication bias. Due to the presence of publication bias (Supplementary Fig 2), we employed a trim and fill analysis and one study was added and the prevalence of cerebral palsy becomes 3·193 (Supplementary Fig 3).

Sensitivity analysis: We also employed a leave-one-out sensitivity analysis to identify the potential source of heterogeneity in the analysis of the prevalence of cerebral palsy in Africa. The results of this sensitivity analysis showed that the findings were not dependent on a single study. Our pooled estimated prevalence of cerebral palsy varied from 2·76 (2·12–3·39) to 3·55 (2·84–4·25) after the deletion of a single study (Supplementary Fig 4).

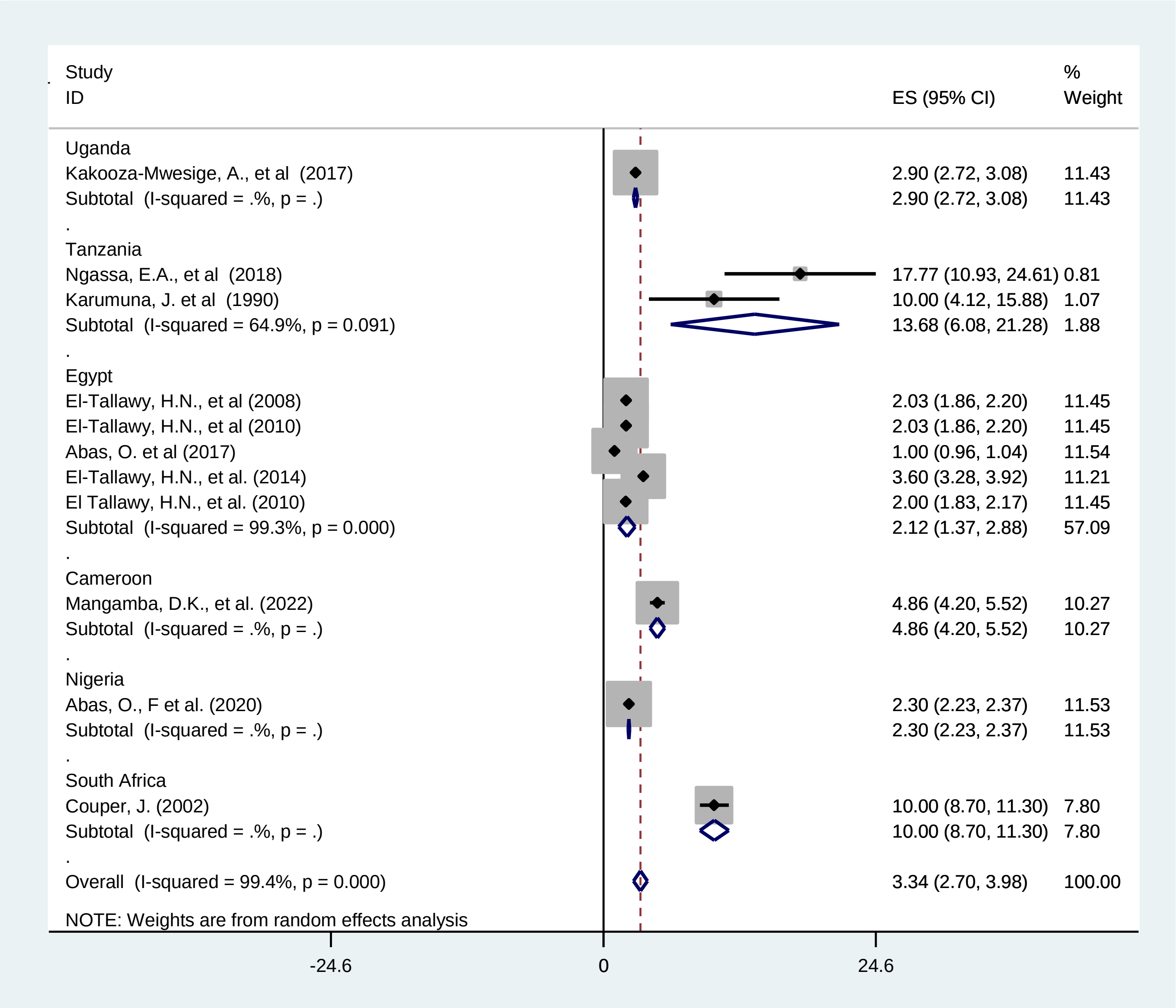
Study
ID
ES (95% CI)
%
Weight
Uganda
Kakooza-Mwesige, A., et al (2017) 2.90 (2.72, 3.08) 11.43
Subtotal (I-squared = .%, p = .) 2.90 (2.72, 3.08) 11.43
Tanzania
Ngassa, E.A., et al (2018) 17.77 (10.93, 24.61) 0.81
Karumuna, J. et al (1990) 10.00 (4.12, 15.88) 1.07
Subtotal (I-squared = 64.9%, p = 0.091) 13.68 (6.08, 21.28) 1.88
Egypt
El-Tallawy, H.N., et al (2008) 2.03 (1.86, 2.20) 11.45
El-Tallawy, H.N., et al (2010) 2.03 (1.86, 2.20) 11.45
Abas, O. et al (2017) 1.00 (0.96, 1.04) 11.54
El-Tallawy, H.N., et al. (2014) 3.60 (3.28, 3.92) 11.21
El Tallawy, H.N., et al. (2010) 2.00 (1.83, 2.17) 11.45
Subtotal (I-squared = 99.3%, p = 0.000) 2.12 (1.37, 2.88) 57.09
Cameroon
Mangamba, D.K., et al. (2022) 4.86 (4.20, 5.52) 10.27
Subtotal (I-squared = .%, p = .) 4.86 (4.20, 5.52) 10.27
Nigeria
Abas, O., F et al. (2020) 2.30 (2.23, 2.37) 11.53
Subtotal (I-squared = .%, p = .) 2.30 (2.23, 2.37) 11.53
South Africa
Couper, J. (2002) 10.00 (8.70, 11.30) 7.80
Subtotal (I-squared = .%, p = .) 10.00 (8.70, 11.30) 7.80
Overall (I-squared = 99.4%, p = 0.000) 3.34 (2.70, 3.98) 100.00
NOTE: Weights are from random effects analysis
-24.6
0
24.6

Figure 3: Forest plot showing subgroup analysis for prevalence of cerebral palsy among children in by countries in Africa

Clinical characteristics of cerebral palsy in Africa

Spastic cerebral palsy

Pooled prevalence: Spastic cerebral palsy is the most common type of cerebral palsy in Africa. It is characterized by jerky movements, muscle tightness and joint stiffness. Six of the included studies have reported the magnitude of spastic cerebral palsy in percentage.[(12d,13,25a,25b,25e,25k)] The prevalence of spastic cerebral palsy was ranged from 23·00%(95% CI: 15·90, 30·10) to 88·90 (95% CI: 85·83, 91·97). The random-effects model analysis from those studies revealed that, the pooled prevalence of spastic cerebral palsy in Africa was found to be 69·30% (95% CI: 66·76, 71·83) (95% CI: I^2 = 99·5%; p < 0·001) (Fig. 4).

Subgroup analysis: The subgroup analysis was done through stratified by country, and sample size. Based on this, the prevalence of spastic cerebral palsy was found to be 23·00%(95% CI: 15·90, 30·10) in Uganda, 70·13% (65·54,74·72) in Egypt, 69·90%(69·68, 70·12) in Nigeria, and 88·9%(85·83, 91·97) in Ethiopia (Supplementary Fig. 5).

Publication bias: A funnel plot showed symmetrical distribution. The Egger's regression test-value was 0·468, which indicated that, the absence of publication bias. As a result, we didn't conduct trim and fill analysis (Supplementary Fig. 6).

Sensitivity analysis: We also employed a leave-one-out sensitivity analysis to identify the potential source of heterogeneity in the analysis of the prevalence of spastic cerebral palsy in Africa. The results of this sensitivity analysis showed that the findings were not

dependent on a single study. Our pooled estimated prevalence of spastic cerebral palsy varied from 66·11% (63·53–68·70) to 73·05% (70·64–75·45) after the deletion of a single study (Supplementary Fig. 7).

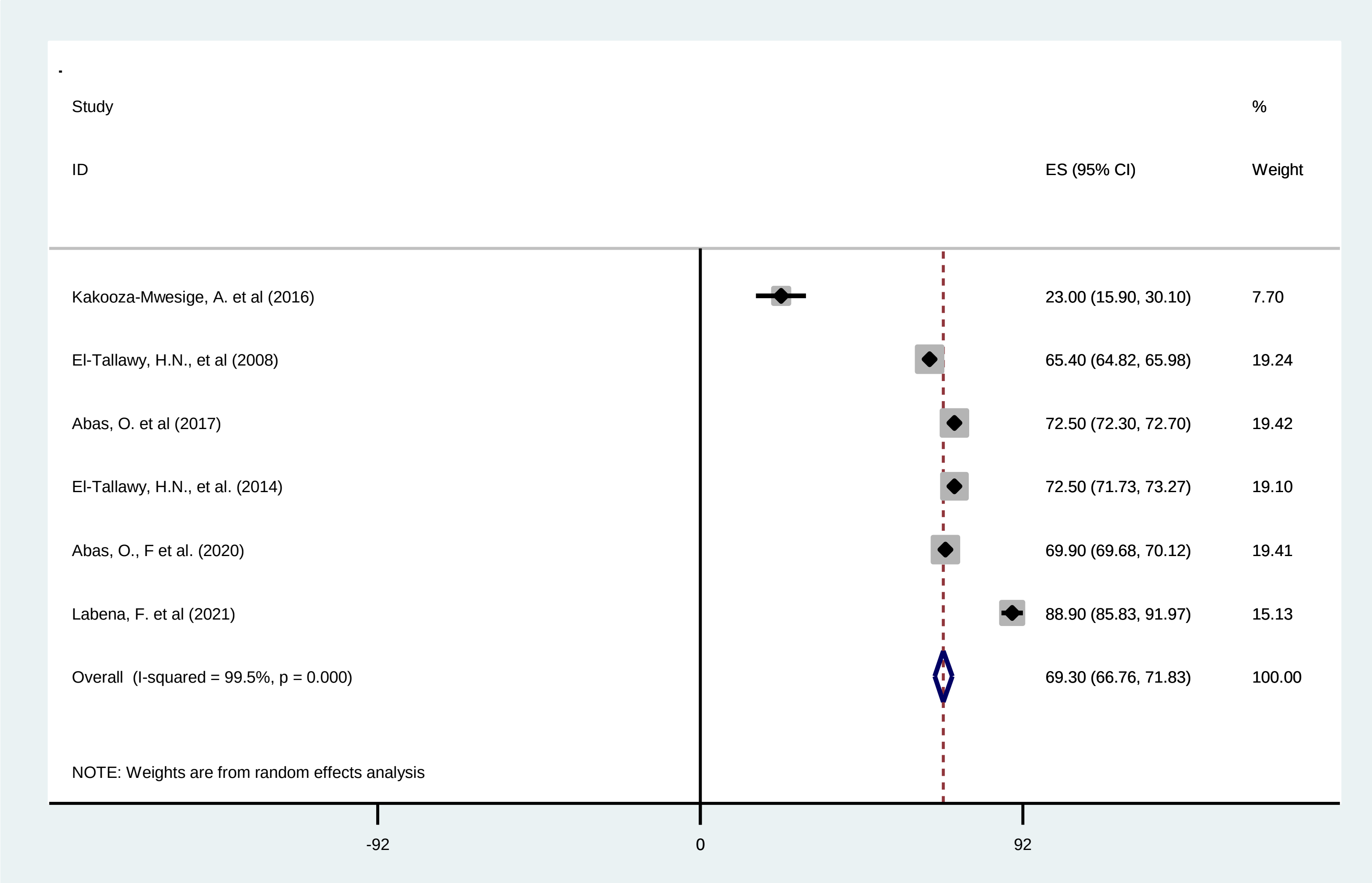

Figure 4: Forest plot showing the pooled prevalence of spastic cerebral palsy among children in Africa

Quadriplegic cerebral palsy in Africa

Pooled prevalence: Dyskinetic CP is characterized by involuntary, uncontrolled, and recurring movements with fluctuating muscle tone.[3,20] Most of the included studies (n = 5) have reported the magnitude of quadriplegic cerebral palsy in percentage from total cerebral palsy cases.[12d,13,25a,25e,25g,25h] The prevalence of unilateral cerebral palsy was ranged from 27.60%(95% CI: 18.56, 36.64) to 62·50 (95% CI: 57·77, 67·23). The random-effects model analysis from those studies revealed that, the pooled prevalence of quadriplegic cerebral palsy in Africa was found to be 41·49% (95% CI: 33·16, 49·81) (95% CI: I^2 = 99·7%; p < 0·001) (Fig. 5).

Subgroup analysis: The subgroup analysis was done through stratified by country. Based on this, the prevalence of quadriplegic unilateral cerebral palsy was found to be 36·40%(24·44-48·35) in Egypt, 62·5% (57·77,67·23) in Ethiopia, 27·60% (18·56, 36·64) in South Africa, and 43·50% (34·15, 52·85) in Sudan (Supplementary Fig. 8).

Publication bias: A funnel plot showed symmetrical distribution. The Egger's regression test-value was 0·360, which indicated that, the absence of publication bias. As a result, we didn't conduct trim and fill analysis (Supplementary Fig. 9).

Sensitivity analysis: We also employed a leave-one-out sensitivity analysis to identify the potential source of heterogeneity in the analysis of the prevalence of quadriplegic cerebral palsy in Africa. The results of this sensitivity analysis showed that the findings were

not dependent on a single study. Our pooled estimated prevalence of cerebral palsy varied from 41·04% (22·44–59·65) to 44·45% (35·26–53·64) after the deletion of a single study (Supplementary Fig. 10).

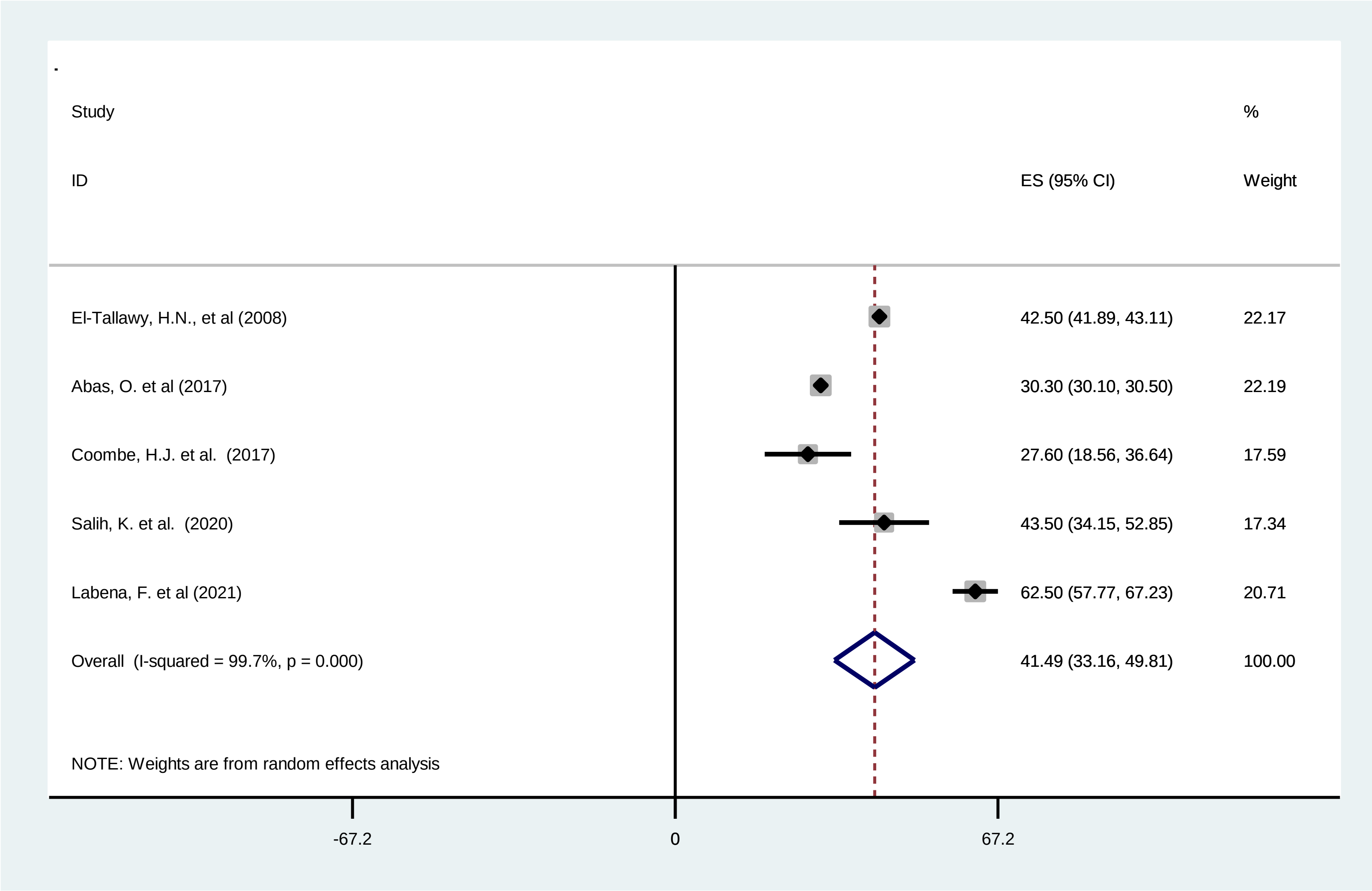

Figure 5: Forest plot showing the pooled prevalence of quadriplegic cerebral palsy among children in Africa

Ataxic cerebral palsy in Africa

Pooled prevalence: Ataxic cerebral palsy characterized by trouble with balance and coordination.(26) Most of the included studies (n = 11) have reported the prevalence of ataxic cerebral palsy per 1000.(12d,13,25a,25b,25d-h,25k) The prevalence of cerebral palsy was ranged from 1·4(95% CI: 0·25, 2·55) to 9·80 (95% CI: 9·66, 9·94). The random-effects model analysis from those studies revealed that, the pooled prevalence of cerebral palsy in Africa was found to be 5·36 (95% CI: 3·22, 7·50) (95% CI: I^2 = 99·8%; p < 0·001) (Fig. 6).

Subgroup analysis: The subgroup analysis was done through stratified by country, and sample size. Based on this, the prevalence of ataxic cerebral palsy was found to be 5·38(2·02-12·78) in Uganda, 9·00(3·39-14·61) in Tanzania, 4·90 (2·46, 7·35) in Egypt, 2·09 (0·23, 3·96) in Ethiopia, 9·60 (3·64, 15·56) in South Africa and 10·00 (8·70,11·30) in Sudan (Supplementary Fig. 11).

Publication bias: A funnel plot showed symmetrical distribution. The Egger's regression test-value was 0·633, which indicated that, the absence of publication bias. Due to the absence of publication bias, we did not employ a trim and fill analysis (Supplementary Fig. 12).

Sensitivity analysis: We also employed a leave-one-out sensitivity analysis to identify the potential source of heterogeneity in the analysis of the prevalence of ataxic cerebral palsy in Africa. The results of this sensitivity analysis showed that the findings were not

dependent on a single study. Our pooled estimated prevalence of cerebral palsy varied from 4·71 (2·74–6·67) to 5·81 (3·56–8·07) after the deletion of a single study (Supplementary Fig. 12).

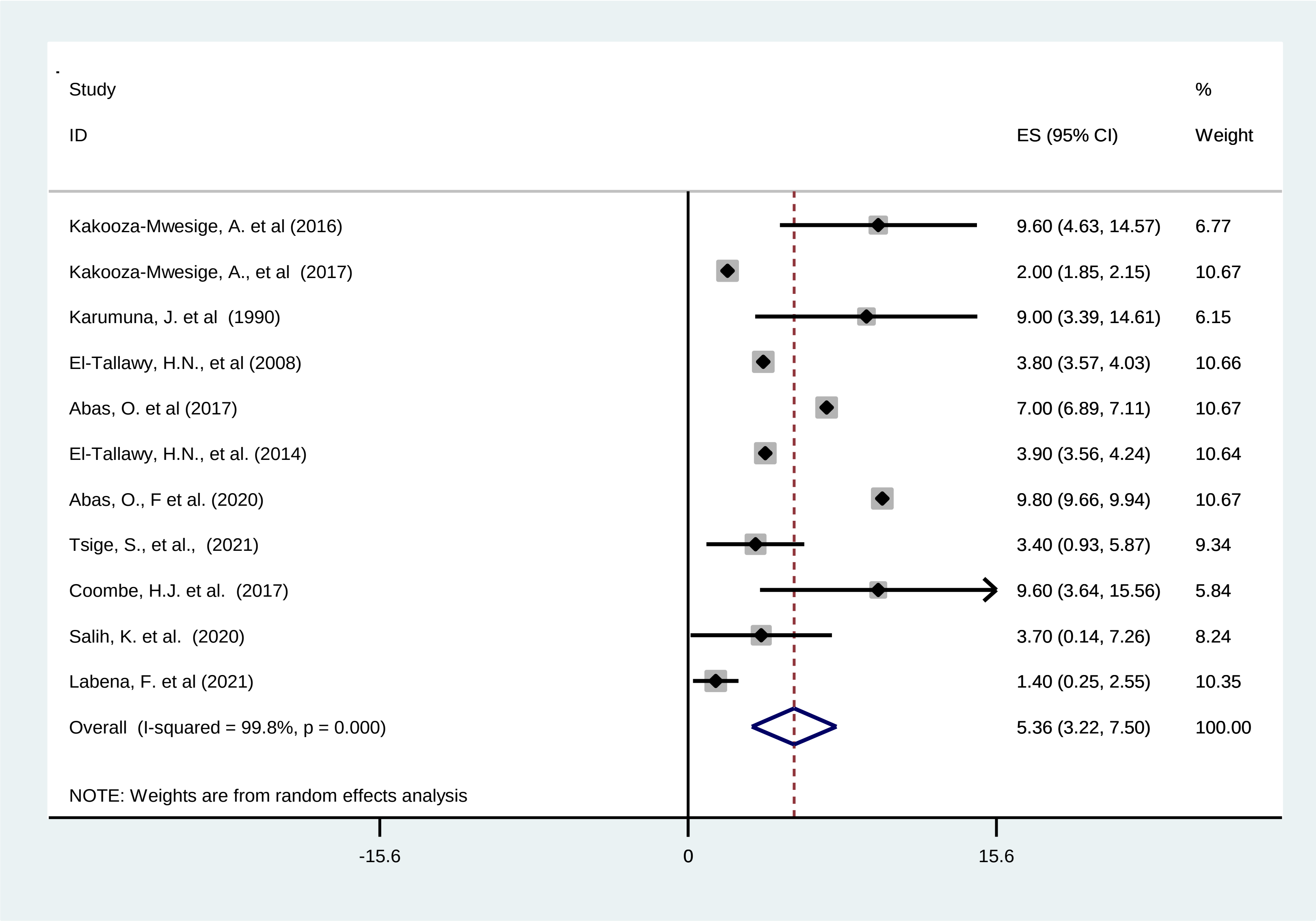
Study
ID
ES (95% CI)
%
Weight
Kakooza-Mwesige, A. et al (2016) 9.60 (4.63, 14.57) 6.77
Kakooza-Mwesige, A., et al (2017) 2.00 (1.85, 2.15) 10.67
Karumuna, J. et al (1990) 9.00 (3.39, 14.61) 6.15
El-Tallawy, H.N., et al (2008) 3.80 (3.57, 4.03) 10.66
Abas, O. et al (2017) 7.00 (6.89, 7.11) 10.67
El-Tallawy, H.N., et al. (2014) 3.90 (3.56, 4.24) 10.64
Abas, O., F et al. (2020) 9.80 (9.66, 9.94) 10.67
Tsige, S., et al., (2021) 3.40 (0.93, 5.87) 9.34
Coombe, H.J. et al. (2017) 9.60 (3.64, 15.56) 5.84
Salih, K. et al. (2020) 3.70 (0.14, 7.26) 8.24
Labena, F. et al (2021) 1.40 (0.25, 2.55) 10.35
Overall (I-squared = 99.8%, p = 0.000) 5.36 (3.22, 7.50) 100.00
NOTE: Weights are from random effects analysis
-15.6
0
15.6

Figure 6: Forest plot showing the pooled prevalence of ataxic cerebral palsy among children in Africa

Dyskinestic cerebral palsy in Africa

Pooled prevalence: Dyskinetic cerebral palsy is characterized by dystonia, athetosis, and chorea.[27] Most of the included studies (n = 8) have reported the magnitude of ataxic cerebral palsy.[12d,13,25a,25b,25e-g,25k] The prevalence of ataxic cerebral palsy was ranged from 3·80%(95% CI: 3·57, 4·03) to 29·80 (95% CI: 20·55, 39·05). The random-effects model analysis from those studies revealed that, the pooled prevalence of unilateral cerebral palsy in Africa was found to be 10·88% (95% CI: 6·26, 15·49) (95% CI: $I^2 = 100\%$; $p < 0.001$) (Fig. 7).

Subgroup analysis: The subgroup analysis was done through stratified by country, and sample size. Based on this, the prevalence of unilateral cerebral palsy was found to be 9·67%(6·92-12·42) in Uganda, 9·90% (2·06, 21·86) in Egypt, 4·60%(4·50, 4·70) in Nigeria, 8·51%(5·94, 11·06) in Ethiopia, and 29·80% (20·55, 39·05) in South Africa (Supplementary Fig. 14).

Publication bias: A funnel plot showed symmetrical distribution. The Egger's regression test-value was 0·728, which indicated that, the absence of publication bias. As a result, we didn't conduct trim and fill analysis (Supplementary Fig. 15).

Sensitivity analysis: We also employed a leave-one-out sensitivity analysis to identify the potential source of heterogeneity in the analysis of the prevalence of dyskinetic cerebral palsy in Africa. The results of this sensitivity analysis showed that the findings were

not dependent on a single study. Our pooled estimated prevalence of cerebral palsy varied from 8·33% (6·37–10·29) to 11·61% (6·89–16·33) after the deletion of a single study (Supplementary Fig. 16).

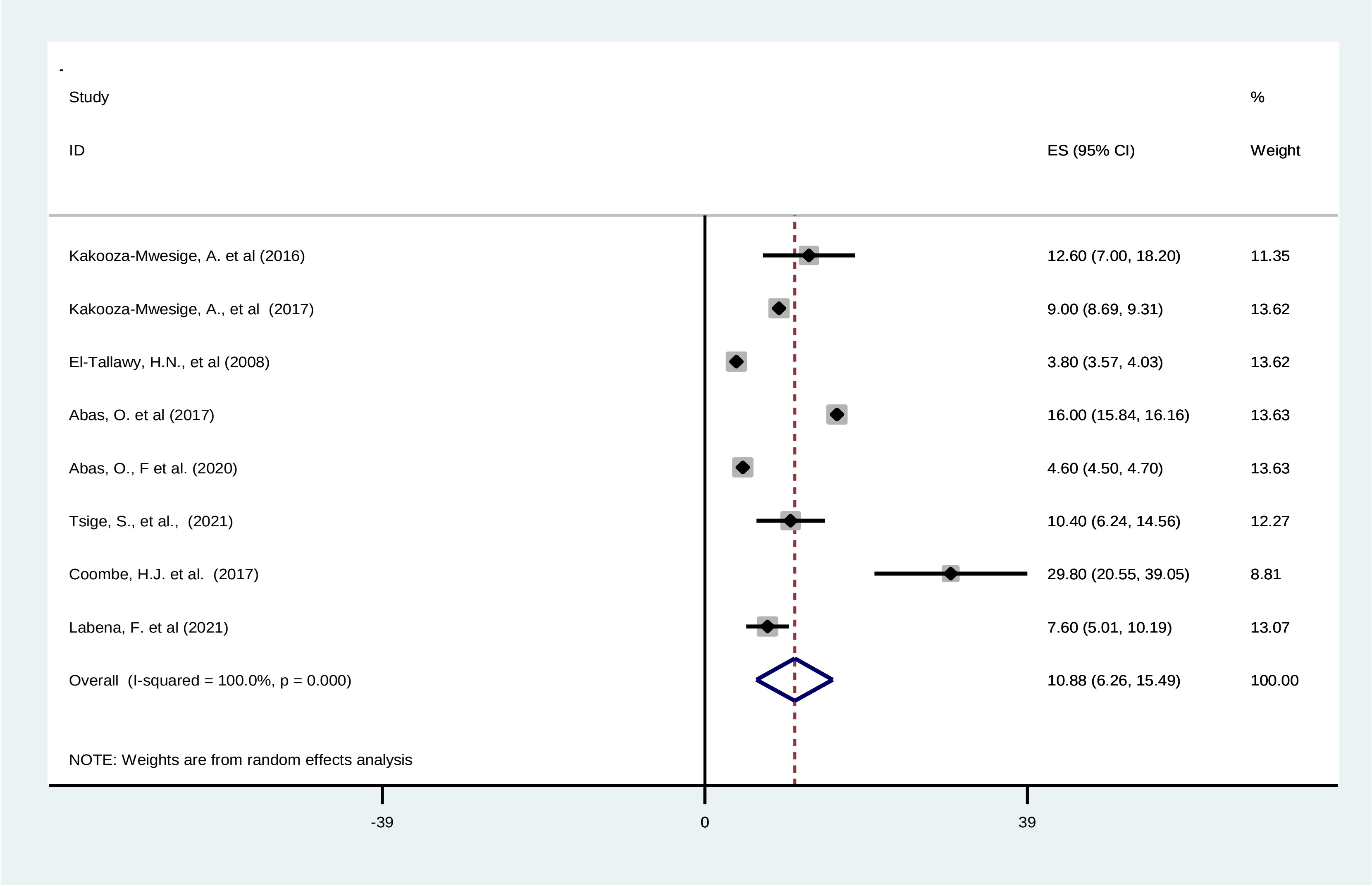

Figure 7: Forest plot showing the pooled prevalence of dyskinestic cerebral palsy among children in Africa

Bilateral cerebral palsy in Africa

Pooled prevalence: Bilateral cerebral palsy is a problem with movement, co-ordination and development which affects both sides of the body.(28) Most of the included studies (n = 10) have reported the magnitude of bilateral cerebral palsy in percentage from total cerebral palsy cases.(12d,13,25a,25b,25e-g,25k) The prevalence of unilateral cerebral palsy was ranged from 9·60%(95% CI: 9·24, 9·96) to 60·40 (95% CI: 53·74, 67·06). The random-effects model analysis from those studies revealed that, the pooled prevalence of bilateral cerebral palsy in Africa was found to be 32·10% (95% CI: 19·25, 44·95) (95% CI: I^2 = 100%; p < 0·001) (Fig. 8).

Subgroup analysis: The subgroup analysis was done through stratified by country, and sample size. Based on this, the prevalence of unilateral cerebral palsy was found to be 41·40%(36·48-46·31) in Uganda, 20·00% (12·16-27·84) in Tanzania, 28·94% (8·96, 66·83) in Egypt, 60·20%(59·97, 60·43) in Nigeria, 34·96 (14·73, 84·4) in Ethiopia, 16·00% (8·59, 23·41) in South Africa, and 13·9% (7·38, 20·42) in Sudan (Supplementary Fig. 17).

Publication bias: A funnel plot showed symmetrical distribution. The Egger's regression test-value was 0·529, which indicated that, the absence of publication bias. As a result, we didn't conduct trim and fill analysis (Supplementary Fig. 18).

Sensitivity analysis: We also employed a leave-one-out sensitivity analysis to identify the potential source of heterogeneity in the analysis of the prevalence of bilateral cerebral palsy in Africa. The results of this sensitivity analysis showed that the findings were not

dependent on a single study. Our pooled estimated prevalence of bilateral cerebral palsy varied from 34·88% (28·31–41·47) to 34·62% (21·11–48·13) after the deletion of a single study (Supplementary Fig. 18).

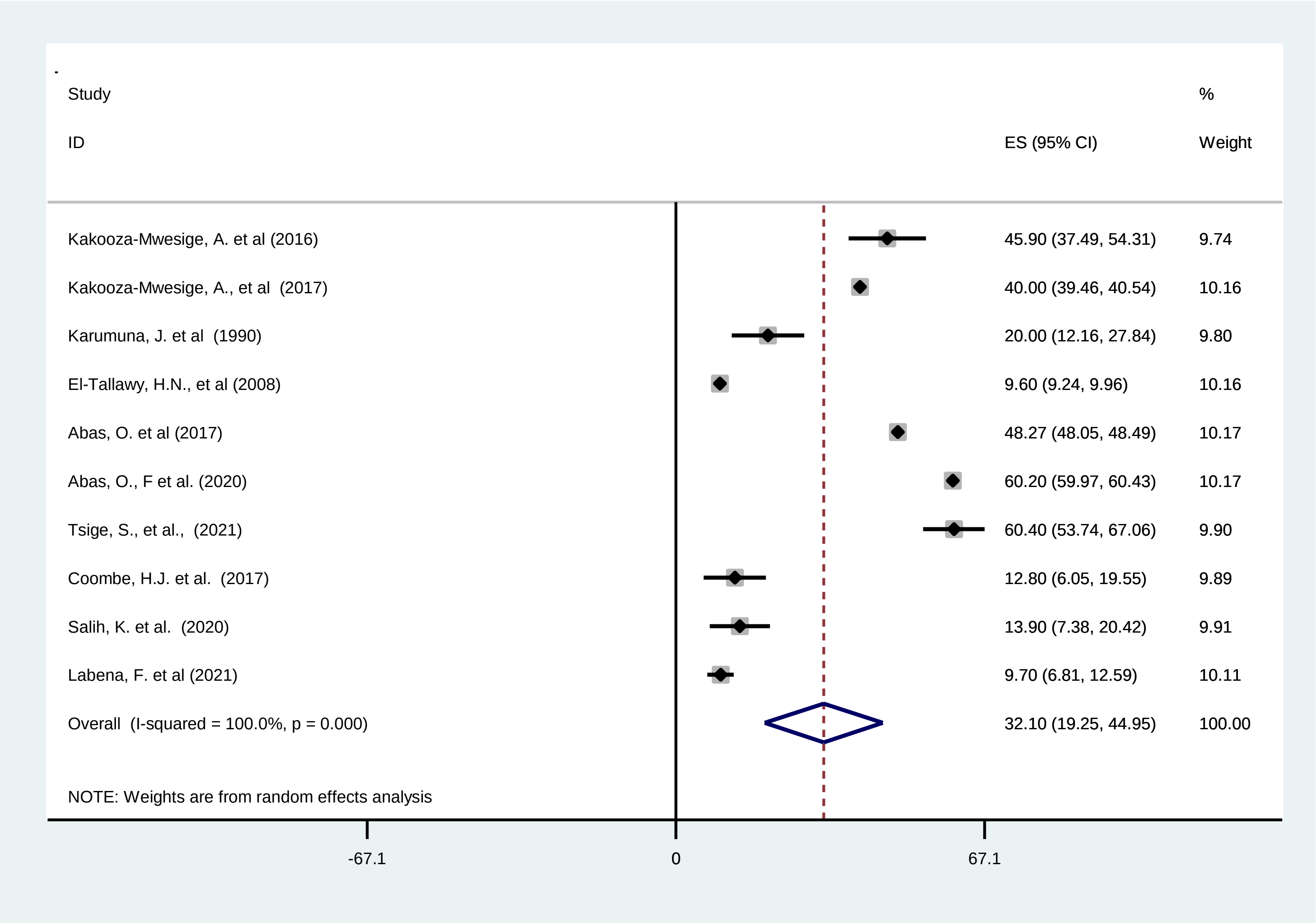

Figure 8: Forest plot showing the pooled prevalence of bilateral cerebral palsy among children in Africa

Unilateral cerebral palsy in Africa

Pooled prevalence: Unilateral Cerebral Palsy is characterized by hemiplegia and hemiparesis, is a condition that affects muscle control and function on one side of the body.[(29)] Most of the included studies (n = 10) have reported the magnitude of unilateral cerebral palsy in percentage from total cerebral palsy cases.[(12d,13,25a,25b,25d-h,25k)] The prevalence of unilateral cerebral palsy was ranged from 13·50%(95% CI: 13·08, 13·92) to 23·70 (95% CI: 16·53, 30·87). The random-effects model analysis from those studies revealed that, the pooled prevalence of unilateral cerebral palsy in Africa was found to be 25·17% (95% CI: 16·84, 33·50) (95% CI: $I^2 = 100\%$; $p < 0·001$) (Fig. 4).

Subgroup analysis: The subgroup analysis was done through stratified by country, and sample size. Based on this, the prevalence of unilateral cerebral palsy was found to be 35·15%(13·30-56·99) in Uganda, 15%(8·00-22·00) in Tanzania, 17·45% (9·71, 25·19) in Egypt, 39·8%(39·57, 40·03) in Nigeria and 10·00 (8·70,11·30), 24·76%(19·61, 29· 92) in Ethiopia, 16·00% (8·59,23·41) in South Africa, and 25·9% (17·64, 34·16) in Sudan (Supplementary Fig 20).

Publication bias: A funnel plot showed symmetrical distribution. The Egger's regression test-value was 0·903, which indicated that, the absence of publication bias. As a result, we didn't conduct trim and fill analysis (Supplementary Fig 20).

Sensitivity analysis: We also employed a leave-one-out sensitivity analysis to identify the potential source of heterogeneity in the analysis of the prevalence of cerebral palsy in Africa. The results of this sensitivity analysis showed that the findings were not dependent

on a single study. Our pooled estimated prevalence of cerebral palsy varied from 22·76% (14·25–31·26) to 26·52% (17·71–35·33) after the deletion of a single study.

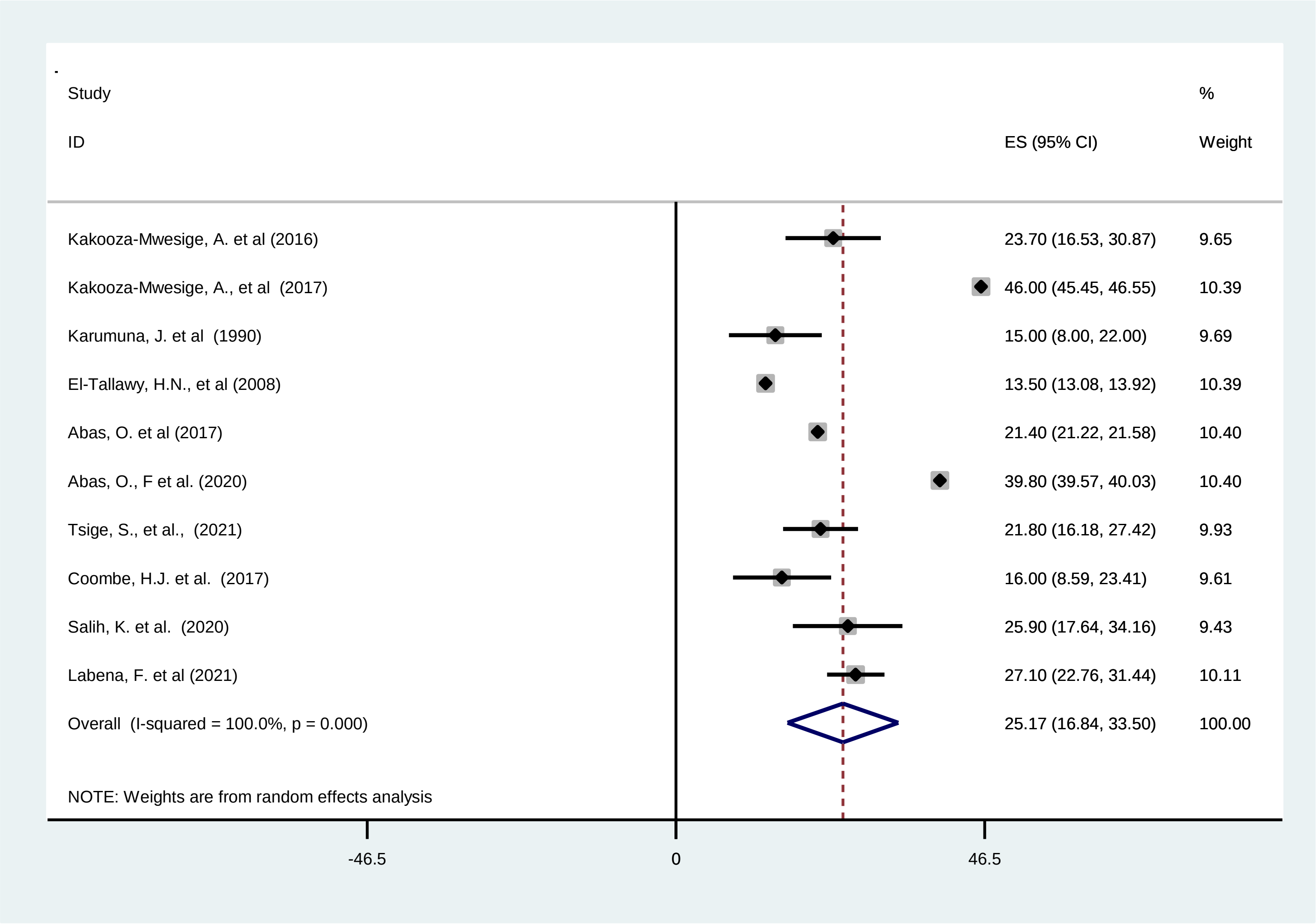

Figure 9: Forest plot showing the pooled prevalence of unilateral cerebral palsy among children in Africa

Mixed type cerebral palsy in Africa

Pooled prevalence: Mixed cerebral palsy occurs when a child exhibits symptoms of more than one type of cerebral palsy.[30] Most of the included studies (n = 10) have reported the magnitude of mixed cerebral palsy in percentage.[13,25a,25b,25d-h,25k] The prevalence of mixed cerebral palsy in Africa was ranged from to 2·00%(1·85, 2·15) to 26·9% (95% CI: 26·36, 27·44). The random-effects model analysis from those studies revealed that, the pooled prevalence of unilateral cerebral palsy in Africa was found to be 8·58% (95% CI: 4·06, 13·11) (95% CI: I^2 = 99·9%; p < 0·001) (Fig. 2).

Subgroup analysis: The subgroup analysis was done through stratified by country, and sample size. Based on this, the prevalence of unilateral cerebral palsy was found to be 4·60%(1·31-10·51) in Uganda, 5·0%(0·73-9·27) in Tanzania, 25·21% (21·88, 28·54) in Egypt, 8·3%(8·17, 8·43) in Nigeria and 2·70 (0·97, 4·42) in Ethiopia, 2·10% (0·80, 5·00) in South Africa, and 2·80% (0·31, 6·59) in Sudan (Supplementary Fig. 23).

Publication bias: A funnel plot showed symmetrical distribution. The Egger's regression test-value was 0·468, which indicated that, the absence of publication bias. As a result, we didn't conduct trim and fill analysis (Supplementary Fig. 24).

Sensitivity analysis: We also employed a leave-one-out sensitivity analysis to identify the potential source of heterogeneity in the analysis of the prevalence of mixed type cerebral palsy in Africa. The results of this sensitivity analysis showed that the findings were

not dependent on a single study. Our pooled estimated prevalence of mixed type cerebral palsy varied from 6·51% (2·57–10·16) to 9·32% (4·52–14·12) after the deletion of a single study (Supplementary Fig. 25).

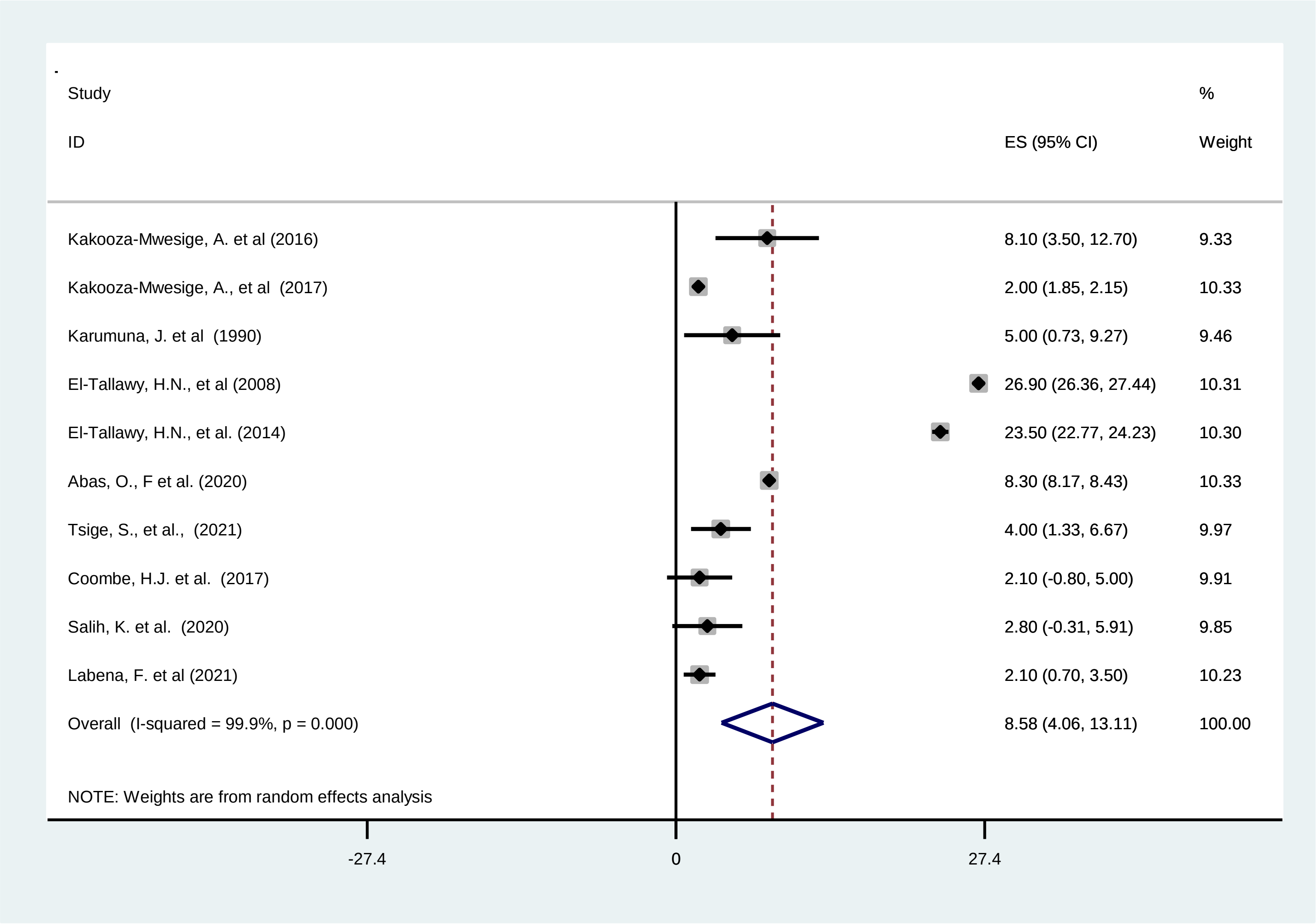

Figure 10: Forest plot showing the pooled prevalence of mixed type cerebral palsy among children in Africa

Discussion

The most prevalent form of motor disability that affects children is cerebral palsy (CP). The brain may malfunction during fetal or prenatal development due to insufficient oxygenation, or it may result from damage to the developing infant's brain during the postpartum period. In this systematic review and meta-analysis, we aimed to assess the magnitude and clinical characteristics of CP among children in Africa. Accordingly, the pooled prevalence of cerebral palsy was found to be 3·3 per 1000 live births (2·69,3·92). This finding is in line with the study done in USA.[(31)] This might be because African descendants were surveyed in that study. However, this finding is higher than CP prevalence in most other countries in United State and Europe which is estimated to be 2-2·5 of 1000 [(32)]. Compared to some studies conducted in Arabic-speaking countries, 1·8,[(33)] Europe, 2·25,[(34)] India, 2·27,[(35)] Canada, 2·57,[(36)] and Japan, 1·88[(37)] the pooled prevalence of cerebral palsy is greater in Africa. This discrepancy might be because of the high magnitude of perinatal complications such as birth asphyxia and neonatal infections in Africa.[(38)] In addition, this might be due to the fact that in Africa, there are challenges to manage cerebral palsy due to: inaccessibility of basic care, limited availability of diagnostic facilities, limited number of trained and expertise personal in managing cerebral palsy and exacerbated by lack of appropriate intervention, medication, surgical procedure and regular physical care.[(39)] In fact, these improper managements might have contributed to the prevalence of cerebral palsy. Furthermore, prematurity, low birthweight, kernicterus, asphyxia and neonatal infections are among the main causes of cerebral palsy, and they are more common in Africa than in western nations.[(39)] this implies there may be a greater proportion of children with more

severe disability in resource-poor countries because of delayed presentation of a range of disorders and absence of early intervention services. Moreover, the clinical spectrum of CP differs from that of affluent countries in resource-poor, developing nations. The discrepancy probably explained by the multifactorial causes of CP and difference in quality of health care delivery between the two regions, low- and middle-income countries and developed countries.[13,40] The burden difference might also be due to absence or inadequacy of inter-disciplinary team approach in developing countries.[16] On the other hand, most studies done in developing countries were institution based and at referral hospitals. Since referral hospitals receive CP patients from different district hospitals, health center and regional hospitals where there are few experts and facilities hence the number of CP patients' data might be aggregated to the referral hospitals.[25c,41] In addition, the high prevalence may be due to improvement in health seeking behavior of the community in developing countries where previously people in Africa believed that having CP patient at home is a curse and tried to hide them at their homes. Tanzania had a higher prevalence of cerebral palsy, at 13·68/1000 (6·08-21·28) compared to studies from other African countries. This might be due to the small sample size included in studies from Tanzania. It is also higher than that of other countries study, Norway 2·1,[42] Thiland 1·0.[43] This difference might be advance technology used, utilization of advance treatment modalities, regular follow up of antenatal care and sample size difference in other countries like Norway and Thiland.

The results of the current review revealed that the spastic type of cerebral palsy affected the majority of children in Africa which accounts 63·4% of all form cerebral palsy. This finding is in line with different studies done in other regions such as north east Italy, Bangladesh and Nepal.[31,44] During assessment of perinatal factors, studies identified that those children with spastic subtype of CP had higher rate of fetal distress and PROM during the perinatal period, higher rate of language and speech difficulty and worse functional impairment

while those with Dyskinetic and Ataxic CP were found to have higher rate of precipitated labor and deep jaundice during the neonatal period.[45] These findings may indicate that spastic subtype of CP might be related with the high rate of perinatal hypoxic insult in Africa as in cases of fetal distress, while dyskinetic and ataxic forms may be associated with bleeding and injuries to the deep grey matters of the brain that can happen in cases of precipitated labor. Children with severe forms of CP were shown to have lower levels of communication function, according to a study from Sweden that demonstrated the relationship between communication function and gross and fine motor and cognitive function.[46] Numerous severe (quadriplegic) bilateral spastic type of CP arises from injuries to the full-term brain due to complications during the birth process such as birth asphyxia or acquired central nervous system infections like meningitis.[47] Study done in Misurata Hospital -LIBYA around 50% of cases had malnutrition and anemia.[48] In Greece study, nearly one-third of patients suffered from iron deficiency anemia.[49] Children with disabilities and their families in African countries are frequently excluded from society because of stigmatization. Most of these children consequently confront many challenges socially, economically, and politically because they are often denied the basics of health care, education, socialization, and recognition.

This systematic review and meta-analysis have limitations. There is variation between African countries in terms of culture, the economic profile, political stability, research funding and infrastructure, and health care systems. This makes it very difficult to generalize the results of this review across all countries. Furthermore, differences in methodology, including hospital versus community sittings, in the included studies also contributed to high levels of heterogeneity. Despite these limitations, these meta-analyses provide a picture of CP and its types among children in Africa.

Conclusions and Recommendations

The overall prevalence of cerebral palsy in Africa found to be high (3·34 /1000) (95% CI: 2·70, 3·98). compared to findings from developed countries. spastic, and quadriplegic cerebral palsy were found to be the most common with pooled prevalence of 69·30% (95% CI: 66·76, 71·83) and 41·49% (95% CI: 33·16, 49·81) respectively. Governmental and non-governmental organizations should target their effort on the prevention, early detection and management of cerebral palsy in Africa. Large multicenter studies, qualitative or mixed-methods studies assessing patient and family understanding of CP, access to resources, and barriers to care are virtually absent in Africa to address recent evidence gap. In addition, there is a need for longitudinal studies that would assess outcomes over time for patients with CP as well as for randomized controlled trials of community-based treatment strategies that would be appropriate in an African setting.

Author Contributions

Conceptualization: Biruk Beletew Abate, Kindie Mekuria, Alemu Birara, Molla Azmeraw. **Data curation**: Biruk Beletew Abate, Kindie Mekuria, Addis Wendimagegn, Molla Azmeraw. **Formal analysis, validation, and visualization**: Biruk Beletew Abate, Alemu Birara, Addis Wendimagegn, Molla Azmeraw, Gebremeskel **Writing – original draft:** Biruk Beletew Abate, Kindie Mekuria, Alemu Birara, Molla Azmeraw, Gebremeskel. **Writing – review & editing:** Biruk Beletew Abate, Kindie Mekuria, Addis Wendimagegn, Gebremeskel

Conflicts of interest: The authors declare no conflict of interest

Funding: There was no funding source for this study.

Data availability statement

Data sharing is available on request to the authors, and if data is from a register it would require approval from the individual register

Acknowledgments

Not applicable

References

1. Rosenbaum P, Paneth N, Leviton A, Goldstein M, Bax M, Damiano D. & Jacobsson, B.(2007). A report: the definition and classification of cerebral palsy April 2006. Dev Med Child Neurol Suppl.109(1):8-14.

2. Florin T, Ludwig S, Aronson PL, Werner HC. Netter's pediatrics: Elsevier Health Sciences; 2011.

3. Rosenbaum P, Paneth N, Leviton A, Goldstein M, Bax M, Damiano D, et al. A report: the definition and classification of cerebral palsy April 2006. Dev Med Child Neurol Suppl. 2007;109(suppl 109):8-14.

4. Centers for Disease Control and Prevention. Data and statistics for prevalence and characteristics of cerebral palsy available on https://www.cdc.gov/cp 2021.

5. Fritz H, Sewell-Roberts C. Family stress associated with cerebral palsy. Cerebral palsy. 2020:515-45.

6. Shevell M, Dagenais L, Oskoui M, editors. The epidemiology of cerebral palsy: new perspectives from a Canadian registry. Seminars in pediatric neurology; 2013.

7. Engle PL, Black MM, Behrman JR, De Mello MC, Gertler PJ, Kapiriri L, et al. Strategies to avoid the loss of developmental potential in more than 200 million children in the developing world. The lancet. 2007;369(9557):229-42.

8. IALP J. Report on the WHO Conference on Disability and Rehabilitation, September 14, 2007. Folia Phoniatr Logop. 2008;60:54-6.

9. Hollung SJ, Vik T, Lydersen S, Bakken IJ, Andersen GL. Decreasing prevalence and severity of cerebral palsy in Norway among children born 1999 to 2010 concomitant with improvements in perinatal health. european journal of paediatric neurology. 2018;22(5):814-21.

10. Petersen TG, Forthun I, Lange T, Villadsen SF, Andersen A-MN, Uldall P, et al. Cerebral palsy among children of immigrants in Denmark and the role of socioeconomic status. european journal of paediatric neurology. 2019;23(3):507-16.

11. Khandaker G, Muhit M, Karim T, Smithers-Sheedy H, Novak I, Jones C, et al. Epidemiology of cerebral palsy in Bangladesh: a population-based surveillance study. Developmental Medicine & Child Neurology. 2019;61(5):601-9.

12. aEl Tallawy HN, Farghaly WM, Rageh TA, Shehata GA, Metwaly NA, Elftoh NA, et al. Epidemiology of major neurological disorders project in Al Kharga district, New Valley, Egypt. Neuroepidemiology. 2010;35(4):291-7; bCouper J. Prevalence of childhood disability in rural KwaZulu Natal. South African Medical Journal. 2002;92(7):549-52; cKakooza-Mwesige A, Forssberg H, Eliasson A-C, Tumwine JK. Cerebral palsy in children in Kampala, Uganda: clinical subtypes, motor function and co-morbidities. BMC research notes. 2015;8(1):1-10; dAbas O, Abdelaziem F, Kilany A. Clinical spectrum of cerebral palsy and associated disability in South Egypt: a local survey study. Open access Macedonian journal of medical sciences. 2017;5(1):37.

13. Kakooza-Mwesige A, Andrews C, Peterson S, Mangen FW, Eliasson AC, Forssberg H. Prevalence of cerebral palsy in Uganda: a population-based study. The Lancet Global Health. 2017;5(12):e1275-e82.

14. aOdding E, Roebroeck ME, Stam HJ. The epidemiology of cerebral palsy: incidence, impairments and risk factors. Disability and rehabilitation. 2006;28(4):183-91; bBearden DR, Monokwane B, Khurana E, Baier J, Baranov E, Westmoreland K, et al. Pediatric cerebral palsy in Botswana: etiology, outcomes, and comorbidities. Pediatric neurology. 2016;59:23-9; cPrusiner SB, Woerman AL, Mordes DA, Watts JC, Rampersaud R, Berry DB, et al. Evidence for α-synuclein prions causing multiple system atrophy in humans with parkinsonism. Proceedings of the National Academy of Sciences. 2015;112(38):E5308-E17.

15. Shevell M, Miller SP, Scherer SW, Yager JY, Fehlings MG, editors. The Cerebral Palsy Demonstration Project: A multidimensional research approach to cerebral palsy. Seminars in Pediatric Neurology; 2011: Elsevier.

16. Zhakupova M, Ospanova D, Issayeva R, Nurbakyt A, Seysebayeva R, Myrzabekova G, et al. Assessment of the main risk factors of development of children's cerebral palsy. Revista Latinoamericana de Hipertension. 2019;14(4):250-5.

17. Reddihough DS, Collins KJ. The epidemiology and causes of cerebral palsy. Australian Journal of physiotherapy. 2003;49(1):7-12.

18. Damiano D, Forssberg H. Poor data produce poor models: Children with developmental disabilities deserve better. The Lancet Global Health. 2019;7(2):e188.

19. Gincota Bufteac E, Andersen GL, Torstein V, Jahnsen R. Cerebral palsy in Moldova: subtypes, severity and associated impairments. BMC pediatrics. 2018;18(1):1-9.

20. Cans C. Surveillance of cerebral palsy in Europe: a collaboration of cerebral palsy surveys and registers. Developmental Medicine & Child Neurology. 2000;42(12):816-24.

21. Donald KA, Samia P, Kakooza-Mwesige A, Bearden D, editors. Pediatric cerebral palsy in Africa: a systematic review. Seminars in pediatric neurology; 2014: Elsevier.

22. Rohwerder B. Disability stigma in developing countries. 2018.

23. Institute JB. Meta-analysis of statistics: assessment and review instrument (JBI mastari). Adelaide. 2006;20032007.

24. Moher D, Liberati A, Tetzlaff J, Altman DG, Group P. Preferred reporting items for systematic reviews and meta-analyses: the PRISMA statement. PLoS medicine. 2009;6(7):e1000097.

25. aLabena F. Prevalence of Cerebral Palsy, Its Associated Factors And Mri Patho-Anatomic Findings Among Patients Attending Pediatric Neurology Clinic In Tikur Anbessa Specialized Hospital By: . 2021; bKakooza-Mwesige A. Cerebral palsy in Mulago hospital, Uganda: comorbidity, diagnosis and cultural adaptation of an assessment tool: Karolinska Institutet (Sweden); 2016; cNgassa EA. Assessment of prevalence and risk factors for cerebral palsy: A case of Mbeya zonal referral hospital: The Open University of Tanzania; 2018; dKarumuna J, Mgone C. Cerebral palsy in Dar Es Salaam. Central african journal of medicine. 1990;36(1):8-10; eEl-Tallawy HN, Farghaly WM, Shehata GA, Rageh TA, Metwally NA, Badry R, et al. Cerebral palsy in Al-Quseir City, Egypt: prevalence, subtypes, and risk factors. Neuropsychiatric Disease and Treatment. 2014:1267-72; fTsige S, Moges A, Mekasha A, Abebe W, Forssberg H. Cerebral palsy in children: subtypes, motor function and associated impairments in Addis Ababa, Ethiopia. BMC pediatrics. 2021;21:1-11; gCoombe HJ. The clinical presentation of Cerebral Palsy in children in rural Kwazulu-Natal, South Africa. 2017; hSalih K. Pattern of cerebral palsy among Sudanese children less than 15 years of age. Cureus. 2020;12(3); iEl-Tallawy HN, Farghaly

WM, Shehata GA, Metwally NA, Rageh TA, Abo-Elfetoh N. Epidemiology of cerebral palsy in El-Kharga District-New Valley (Egypt). Brain and Development. 2011;33(5):406-11; jMangamba DK, Enyama D, Foko LK, Tankou J, Njinkui DN, Essome H, et al. Epidemiological, clinical, and treatment-related features of children with cerebral palsy in Cameroon: A hospital-based study. Archives de Pédiatrie. 2022;29(3):219-24; kDuke R, Torty C, Nwachukwu K, Ameh S, Kim M, Eneli N, et al. Clinical features and aetiology of cerebral palsy in children from Cross River State, Nigeria. Archives of Disease in Childhood. 2020;105(7):625-30.

26. Elshafey MA, Abdrabo MS, Elnaggar RK. Effects of a core stability exercise program on balance and coordination in children with cerebellar ataxic cerebral palsy. Journal of Musculoskeletal & Neuronal Interactions. 2022;22(2):172.

27. Albright AL. Spasticity and movement disorders in cerebral palsy. SAGE Publications Sage CA: Thousand Oaks, CA; 1996. p. S1-S4.

28. Utley A, Steenbergen B. Discrete bimanual co-ordination in children and young adolescents with hemiparetic cerebral palsy: recent findings, implications and future research directions. Pediatric Rehabilitation. 2006;9(2):127-36.

29. Domagalska-Szopa M, Szopa A. Body posture asymmetry differences between children with mild scoliosis and children with unilateral cerebral palsy. BioMed research international. 2013;2013.

30. Ansel BM, Kent RD. Acoustic-phonetic contrasts and intelligibility in the dysarthria associated with mixed cerebral palsy. Journal of Speech, Language, and Hearing Research. 1992;35(2):296-308.

31. Arneson CL, Durkin MS, Benedict RE, Kirby RS, Yeargin-Allsopp M, Braun KVN, et al. Prevalence of cerebral palsy: autism and developmental disabilities monitoring network, three sites, United States, 2004. Disability and health journal. 2009;2(1):45-8.

32. aGottlieb CA, Maenner MJ, Cappa C, Durkin MS. Child disability screening, nutrition, and early learning in 18 countries with low and middle incomes: data from the third round of UNICEF's Multiple Indicator Cluster Survey (2005–06). The Lancet. 2009;374(9704):1831-9; bNovak I, Morgan C, Adde L, Blackman J, Boyd RN, Brunstrom-Hernandez J, et al. Early, accurate diagnosis and early intervention in cerebral palsy: advances in diagnosis and treatment. JAMA pediatrics. 2017;171(9):897-907; cOskoui M, Coutinho F, Dykeman J. Jett, é N., Pringsheim T.(2013). An update on the prevalence of cerebral palsy: a systematic review and meta-analysis Dev Med Child Neurol.55:509-19.

33. Mushta SM, King C, Goldsmith S, Smithers-Sheedy H, Badahdah A-M, Rashid H, et al. Epidemiology of cerebral palsy among children and adolescents in Arabic-speaking countries: a systematic review and meta-analysis. Brain Sciences. 2022;12(7):859.

34. Johnson A. Prevalence and characteristics of children with cerebral palsy in Europe. Developmental medicine and child neurology. 2002;44(9):633-40.

35. Raina SK, Razdan S, Nanda R. Prevalence of cerebral palsy in children< 10 years of age in RS Pura town of Jammu and Kashmir. Journal of tropical pediatrics. 2011;57(4):293-5.

36. Robertson CM, Svenson LW, Joffres MR. Prevalence of cerebral palsy in Alberta. Canadian journal of neurological sciences. 1998;25(2):117-22.

37. Touyama M, Touyama J, Toyokawa S, Kobayashi Y. Trends in the prevalence of cerebral palsy in children born between 1988 and 2007 in Okinawa, Japan. Brain and Development. 2016;38(9):792-9.

38. Schrag SJ, Cutland CL, Zell ER, Kuwanda L, Buchmann EJ, Velaphi SC, et al. Risk factors for neonatal sepsis and perinatal death among infants enrolled in the prevention of perinatal sepsis trial, Soweto, South Africa. The Pediatric infectious disease journal. 2012;31(8):821-6.

39. Donald KA, Kakooza AM, Wammanda RD, Mallewa M, Samia P, Babakir H, et al. Pediatric cerebral palsy in Africa: Where are we? Journal of child neurology. 2015;30(8):963-71.

40. Singhi P, Saini AG. Changes in the clinical spectrum of cerebral palsy over two decades in North India—an analysis of 1212 cases. Journal of tropical pediatrics. 2013;59(6):434-40.

41. Paget A, Mallewa M, Chinguo D, Mahebere-Chirambo C, Gladstone M. “It means you are grounded”–caregivers’ perspectives on the rehabilitation of children with neurodisability in Malawi. Disability and rehabilitation. 2016;38(3):223-34.

42. Andersen GL, Irgens LM, Haagaas I, Skranes JS, Meberg AE, Vik T. Cerebral palsy in Norway: prevalence, subtypes and severity. European journal of paediatric neurology. 2008;12(1):4-13.

43. Chueluecha C, Deeprasertdamrong W, Neekong R, Bamroongya N. Surveying a decade of cerebral palsy prevalence and characteristics at Thammasat university hospital, Thailand. Journal of The Medical Association of Thailand. 2020;103(4):379-86.

44. aCooley WC, Disabilities CoCw. Providing a primary care medical home for children and youth with cerebral palsy. Pediatrics. 2004;114(4):1106-13; bBottos M, Granato T, Allibrio G, Gioachin C, Puato ML. Prevalence of cerebral palsy in north-east Italy from 1965 to 1989. Developmental medicine and child neurology. 1999;41(1):26-39.

45. Monbaliu E, Himmelmann K, Lin J-P, Ortibus E, Bonouvrié L, Feys H, et al. Clinical presentation and management of dyskinetic cerebral palsy. The Lancet Neurology. 2017;16(9):741-9.

46. Himmelmann K, Lindh K, Hidecker MJC. Communication ability in cerebral palsy: a study from the CP register of western Sweden. European Journal of Paediatric Neurology. 2013;17(6):568-74.

47. Kakooza-Mwesige A, Forssberg H, Eliasson A-C, Tumwine JK. Cerebral palsy in children in Kampala, Uganda: clinical subtypes, motor function and co-morbidities. BMC research notes. 2015;8:1-10.

48. Ashour BM, Sewasi M. Risk Factors & Complications of Cerebral Palsy in Misurata Hospital–LIBYA. Scholars Journal of Applied Medical Sciences ISSN. 2013:2320-6691.

49. Papadopoulos A, Ntaios G, Kaiafa G, Girtovitis F, Saouli Z, Kontoninas Z, et al. Increased incidence of iron deficiency anemia secondary to inadequate iron intake in institutionalized, young patients with cerebral palsy. International journal of hematology. 2008;88:495-7.

Appendix

Supplementary Table S1: Search strategy used for one of the databases

Medline/PubMed	Search terms	MeSH (sub-terms in MeSH)
#1	Prevalence<br>Magnitude	Prevalence
#2	Children<br>Infant	Children

	Child<br><br>Childhood	
#3	Cerebral palsy<br><br>Developmental disabilities<br><br>Neurological impairment<br><br>Childhood disability<br><br>Ataxia<br><br>Dyskinesia<br><br>Spastic	Cerebral palsy
#4	Clinical characteristics<br><br>Type	Clinical characteristics
#5	Africa<br><br>Developing countries	..
#1 AND #2 AND #3 AND #4 AND #5		

Table S2: Quality appraisal result of included studies in Africa, using Joanna Briggs Institute (JBI) quality appraisal checklist [16]

Author	Quality assessment questions													
	Q1	Q2	Q3	Q4	Q5	Q6	Q7	Q8	Q9	Q10	Q11	Yes Total	Quality status	Overall appraisal
**Cross-sectional studies**														
Labena, F. et al[25a]	N	Y	N	Y	Y	N	Y	Y	..	..	..	5/8	Low risk	Included
Kakooza-Mwesige, A. et al[25b]	Y	Y	Y	Y	Y	Y	Y	Y	..	..	..	8/8	Low risk	Included
Ngassa, E.A., et al [25c]	Y	Y	UC	Y	Y	N	Y	Y	..	..	..	6/8	Low risk	Included
Kakooza-Mwesige, A., et al [13]	Y	Y	Y	Y	Y	UC	Y	Y	..	..	..	7/8	Low risk	Included
El-Tallawy, HN., et al [25e]	Y	UC	Y	Y	Y	N	Y	Y	..	..	..	6/8	Low risk	Included
El-Tallawy, H.N., et al [25i]	Y	N	Y	Y	Y	UC	Y	Y	..	..	..	6/8	Low risk	Included
Abas, O. et al[12d]	Y	UC	Y	Y	Y	N	Y	Y	..	..	..	6/8	Low risk	Included
El-Tallawy, H.N., et al[25e]	Y	UC	Y	Y	Y	N	Y	Y	..	..	..	6/8	Low risk	Included
Mangamba, D.K., et al [25j]	UC	Y	Y	Y	Y	N	Y	Y	..	..	..	5/8	Low risk	Included
R Duke, et al.[25k]	Y	Y	Y	Y	Y	N	Y	Y	..	..	..	7/8	Low risk	Included
Tsige, S., et al.[25f]	Y	UC	Y	Y	Y	Y	Y	Y	..	..	..	7/8	Low risk	Included

Coombe, H.J. et al. [25g]	Y	UC	Y	Y	Y	N	Y	Y	..	..	..	6/8	Low risk	Included
Couper, J.[12b]	Y	Y	Y	Y	Y	UC	Y	Y	..	..	..	7/8	Low risk	Included
**Cohort studies**														
Karumuna, J. et al[25d]	Y	Y	Y	UC	UC	Y	Y	Y	Y	Y	N	9/11	Low risk	Included
Salih, K. et al. [25h]	Y	UC	Y	Y	UC	Y	Y	Y	Y	Y	Y	10/11	Low risk	Included

Key: Y=yes, N=no, UC=unclear, Q=Question

Supplementary figures

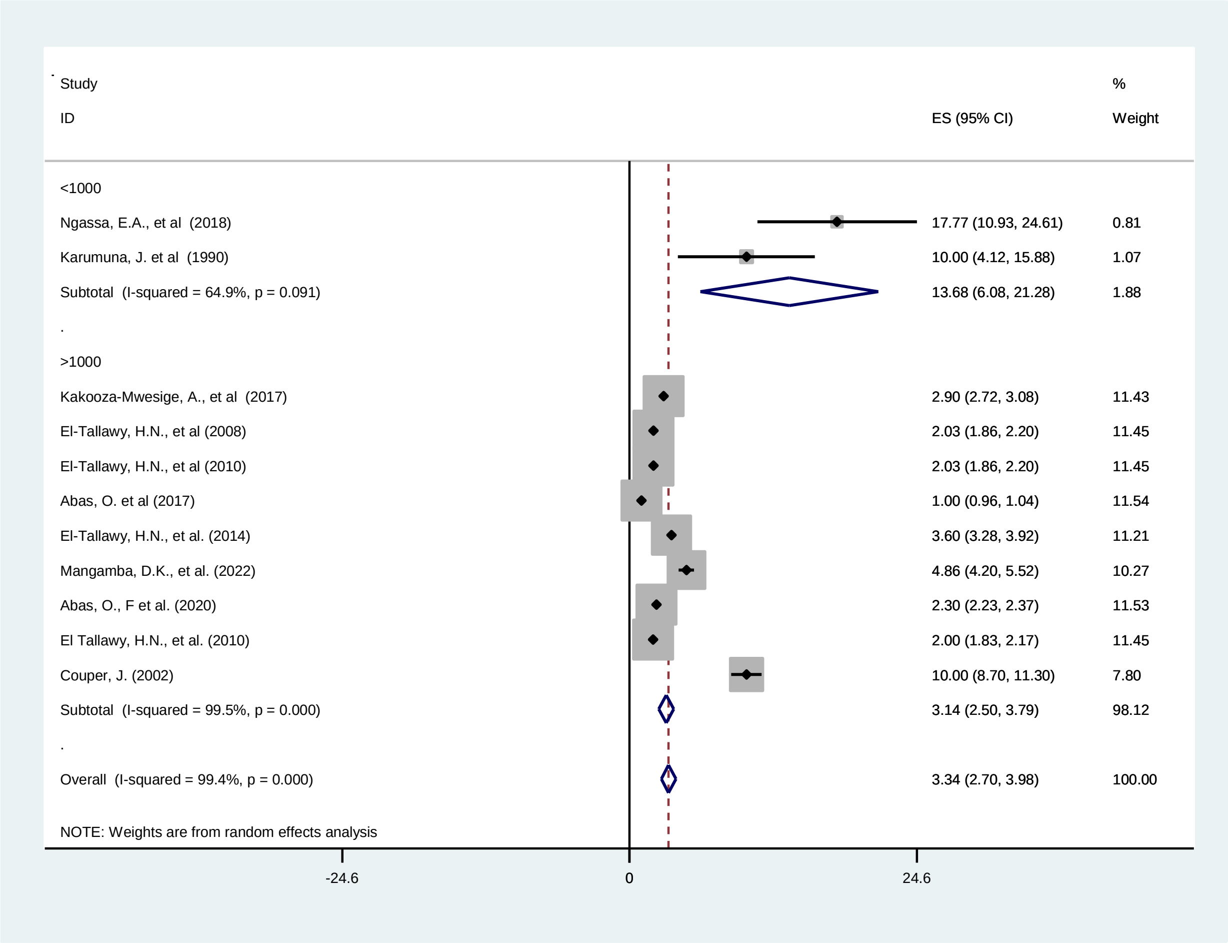

Supplementary Figure 11: Forest plot showing subgroup analysis for prevalence of cerebral palsy among children in by sample size in Africa

```
Tests for Publication Bias

Begg's Test

  adj. Kendall's Score (P-Q) =      -2
          Std. Dev. of Score =   14.58 (corrected for ties)
           Number of Studies =      12
                          z  =   -0.14
                  Pr > |z|  =   0.891
                          z  =    0.07 (continuity corrected)
                  Pr > |z|  =   0.945 (continuity corrected)

Egger's test
```

Std_Eff	Coef.	Std. Err.	t	P>|t|	[95% Conf.	Interval]
slope	1.06554	.2307165	4.62	0.001	.5514717	1.579609
bias	12.53854	3.900915	3.21	0.009	3.846756	21.23032

Supplementary Figure 12 : Shows test of publication bias for prevalence of cerebral palsy among children in by sample size in Africa

```
Meta-analysis

       |  Pooled      95% CI         Asymptotic      No. of
Method |     Est   Lower   Upper  z_value  p_value   studies
-------+----------------------------------------------------
Fixed  |   1.528   1.494   1.562   88.017    0.000     11
Random |   3.340   2.701   3.979   10.243    0.000

Test for heterogeneity: Q= 1767.643 on 10 degrees of freedom (p= 0.000)
Moment-based estimate of between studies variance =  0.921

Trimming estimator: Linear
Meta-analysis type: Random-effects model

iteration |  estimate    Tn    # to trim     diff
----------+--------------------------------------
    1     |     3.340    38         1          66
    2     |     3.219    39         1           2
    3     |     3.219    39         1           0

Filled
Meta-analysis (exponential form)

       |  Pooled      95% CI         Asymptotic      No. of
Method |     Est   Lower   Upper  z_value  p_value   studies
-------+----------------------------------------------------
Fixed  |   4.608   4.454   4.767   88.000    0.000     12
Random |  25.115  13.262  47.563    9.894    0.000

Test for heterogeneity: Q= 1781.226 on 11 degrees of freedom (p= 0.000)
Moment-based estimate of between studies variance =  0.928
```

Supplementary Figure 13: trim and fill analysis for prevalence of cerebral palsy among children in by sample size in Africa

Study omitted	Estimate	[95% Conf.	Interval]
Kakooza-Mwesige, A. et al (2016)	3.339839	2.7007904	3.9788876
Ngassa, E.A., et al (2018)	3.2187543	2.5806565	3.8568523
Kakooza-Mwesige, A., et al (2017)	3.3782389	2.71367	4.0428081
Karumuna, J. et al (1990)	3.2665396	2.6252239	3.9078555
El-Tallawy, H.N., et al (2008)	3.5406842	2.8356779	4.2456903
El-Tallawy, H.N., et al (2010)	3.5406842	2.8356779	4.2456903
Abas, O. et al (2017)	3.3516836	2.8806479	3.8227191
El-Tallawy, H.N., et al. (2014)	3.2801447	2.6228504	3.937439
Mangamba, D.K., et al. (2022)	3.1464853	2.4876113	3.8053594
Abas, O., F et al. (2020)	3.5894372	2.8129199	4.3659544
Tsige, S., et al., (2021)	3.339839	2.7007904	3.9788876
Coombe, H.J. et al. (2017)	3.339839	2.7007904	3.9788876
Salih, K. et al. (2020)	3.339839	2.7007904	3.9788876
El Tallawy, H.N., et al. (2010)	3.5459454	2.8397815	4.2521091
Couper, J. (2002)	2.7557387	2.1198039	3.3916733
Combined	3.3398389	2.7007903	3.9788875

Supplementary Figure 14: sensitivity analysis for prevalence of cerebral palsy among children in by sample size in Africa

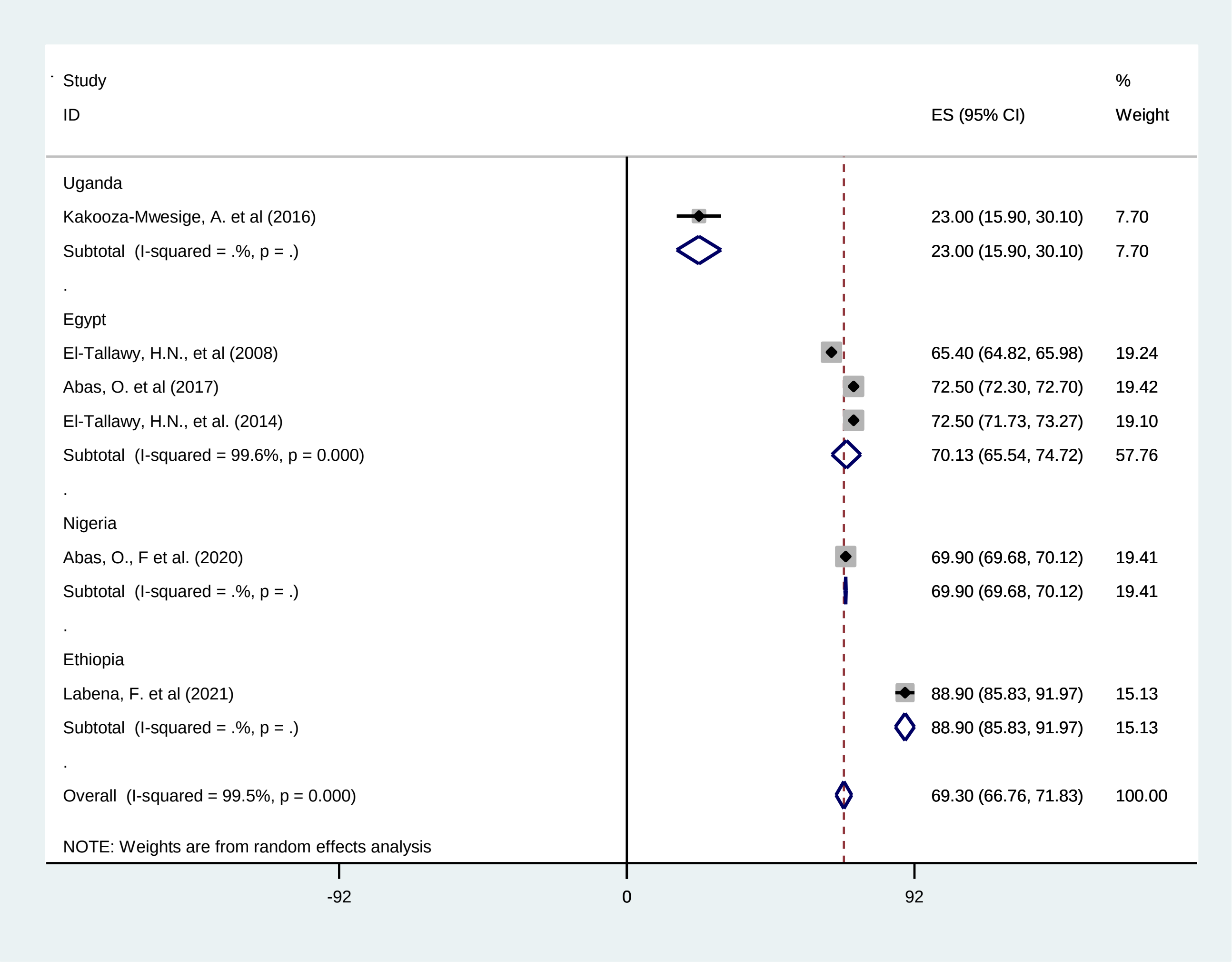

Supplementary Figure 15: subgroup analysis for prevalence of spastic cerebral palsy among children by countries in Africa

Tests for Publication Bias

Begg's Test

adj. Kendall's Score (P-Q) =	1
Std. Dev. of Score =	11.18
Number of Studies =	10
z =	0.09
Pr > |z| =	0.929
z =	0.00 (continuity corrected)
Pr > |z| =	1.000 (continuity corrected)

Egger's test

Std_Eff	Coef.	Std. Err.	t	P>|t|	[95% Conf.	Interval]
slope	5.589804	2.255455	2.48	0.038	.3887155	10.79089
bias	11.01558	14.45599	0.76	0.468	-22.31999	44.35115

Supplementary Figure 16: Publication bias for prevalence of spastic cerebral palsy among children in Africa

Study omitted	Estimate	[95% Conf.	Interval]
Kakooza-Mwesige, A. et al (2016)	73.04713470.64123575.453041		
Ngassa, E.A., et al (2018)	69.29805866.763298		71.832809
Kakooza-Mwesige, A., et al (2017)	69.29805866.76329871.832809		
Karumuna, J. et al (1990)	69.298058	66.763298	71.832809
El-Tallawy, H.N., et al (2008)	70.83965368.414589		73.264717
El-Tallawy, H.N., et al (2010)	69.29805866.763298		71.832809
Abas, O. et al (2017)	67.099449	63.125309	71.073586
El-Tallawy, H.N., et al. (2014)	68.41970865.51406171.325356		
Mangamba, D.K., et al. (2022)	69.29805866.763298		71.832809
Abas, O., F et al. (2020)	67.083244	62.472569	71.693924
Tsige, S., et al., (2021)	69.298058	66.763298	71.832809
Coombe, H.J. et al. (2017)	69.29805866.763298		71.832809
Salih, K. et al. (2020)	69.298058	66.763298	71.832809
El Tallawy, H.N., et al. (2010)	69.29805866.76329871.832809		
Couper, J. (2002)	69.298058	66.763298	71.832809
Labena, F. et al (2021)	66.11338	63.530121	68.696632
Combined	69.298055	66.763299	71.832811

Supplementary Figure 17: Sensitivity analysis for prevalence of spastic cerebral palsy among children in Africa

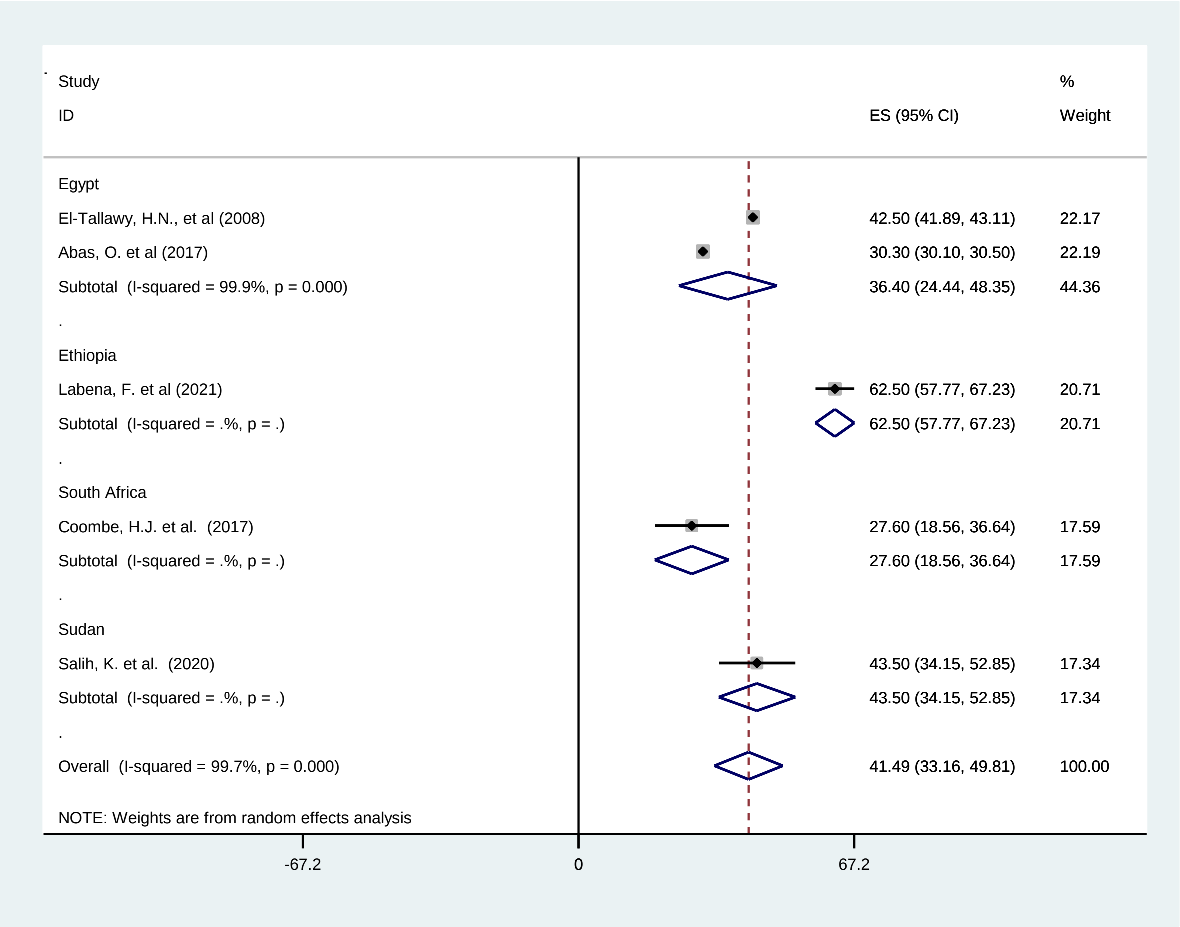

Supplementary Figure 18: Subgroup analysis for prevalence of quadriplegic cerebral palsy among children in Africa

```
Tests for Publication Bias

Begg's Test

  adj. Kendall's Score (P-Q) =        0
          Std. Dev. of Score =     4.08
           Number of Studies =        5
                          z  =     0.00
                   Pr > |z|  =    1.000
                          z  =    -0.24 (continuity corrected)
                   Pr > |z|  =    1.000 (continuity corrected)

Egger's test
```

Std_Eff	Coef.	Std. Err.	t	P>|t|	[95% Conf.	Interval]
slope	30.03194	2.375741	12.64	0.001	22.47127	37.59261
bias	11.73002	10.87846	1.08	0.360	-22.8901	46.3501[illegible]

Supplementary Figure 19: Publication bias for prevalence of quadriplegic cerebral palsy among children in Africa

Study omitted	Estimate	[95% Conf.	Interval]
Kakooza-Mwesige, A. et al	41.486504	33.163143	49.809868
Ngassa, E.A., et al	41.486504	33.163143	49.809868
Kakooza-Mwesige, A., et al	41.486504	33.163143	49.809868
Karumuna, J. et al	41.486504	33.163143	49.809868
El-Tallawy, H.N., et al	41.0424	22.436728	59.648071
Kakooza-Mwesige, A., et al	41.486504	33.163143	49.809868
El-Tallawy, H.N., et al	41.486504	33.163143	49.809868
Abas, O. et al	44.416401	32.09354	56.739262
El-Tallawy, H.N., et al.	41.486504	33.163143	49.809868
Mangamba, D.K., et al.	41.486504	33.163143	49.809868
Abas, O., F et al.	41.486504	33.163143	49.809868
Tsige, S., et al.,	41.486504	33.163143	49.809868
Coombe, H.J. et al.	44.452152	35.263519	53.640789
Salih, K. et al.	41.063976	31.905817	50.222137
El Tallawy, H.N., et al.	41.486504	33.163143	49.809868
Couper, J.	41.486504	33.163143	49.809868
Labena, F. et al	36.000854	27.040903	44.960808
Combined	41.486504	33.163142	49.809866

Supplementary Figure 20: Sensitivity analysis for prevalence of quadriplegic cerebral palsy among children in Africa

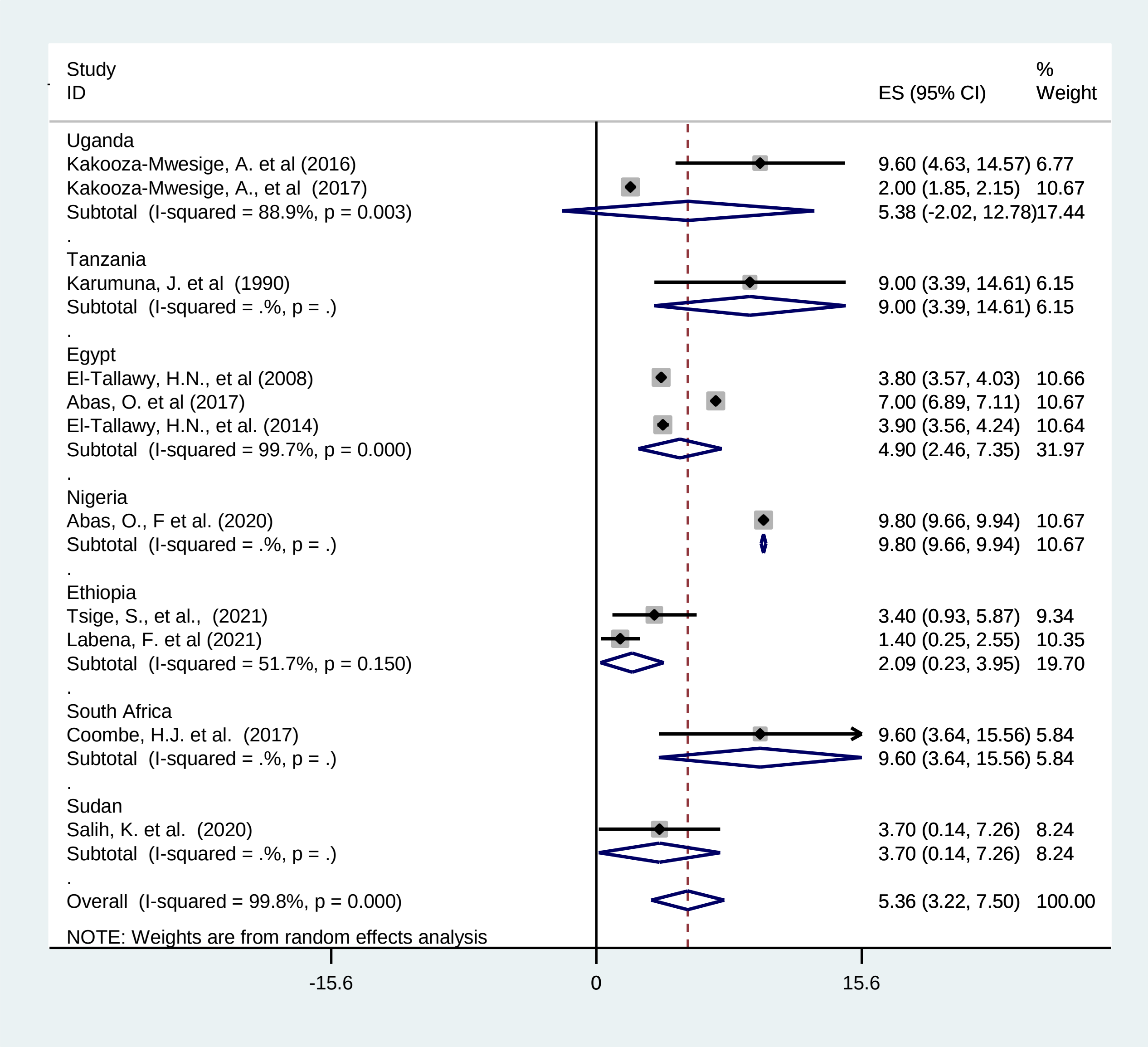
Study
ID
ES (95% CI)
%
Weight
Uganda
Kakooza-Mwesige, A. et al (2016)
9.60 (4.63, 14.57) 6.77
Kakooza-Mwesige, A., et al (2017)
2.00 (1.85, 2.15) 10.67
Subtotal (I-squared = 88.9%, p = 0.003)
5.38 (-2.02, 12.78)17.44
Tanzania
Karumuna, J. et al (1990)
9.00 (3.39, 14.61) 6.15
Subtotal (I-squared = .%, p = .)
9.00 (3.39, 14.61) 6.15
Egypt
El-Tallawy, H.N., et al (2008)
3.80 (3.57, 4.03) 10.66
Abas, O. et al (2017)
7.00 (6.89, 7.11) 10.67
El-Tallawy, H.N., et al. (2014)
3.90 (3.56, 4.24) 10.64
Subtotal (I-squared = 99.7%, p = 0.000)
4.90 (2.46, 7.35) 31.97
Nigeria
Abas, O., F et al. (2020)
9.80 (9.66, 9.94) 10.67
Subtotal (I-squared = .%, p = .)
9.80 (9.66, 9.94) 10.67
Ethiopia
Tsige, S., et al., (2021)
3.40 (0.93, 5.87) 9.34
Labena, F. et al (2021)
1.40 (0.25, 2.55) 10.35
Subtotal (I-squared = 51.7%, p = 0.150)
2.09 (0.23, 3.95) 19.70
South Africa
Coombe, H.J. et al. (2017)
9.60 (3.64, 15.56) 5.84
Subtotal (I-squared = .%, p = .)
9.60 (3.64, 15.56) 5.84
Sudan
Salih, K. et al. (2020)
3.70 (0.14, 7.26) 8.24
Subtotal (I-squared = .%, p = .)
3.70 (0.14, 7.26) 8.24
Overall (I-squared = 99.8%, p = 0.000)
5.36 (3.22, 7.50) 100.00
NOTE: Weights are from random effects analysis
-15.6
0
15.6

Supplementary Figure 21: Subgroup analysis for prevalence of ataxic cerebral palsy among children in Africa

```
Tests for Publication Bias

Begg's Test

  adj. Kendall's Score (P-Q) =      15
          Std. Dev. of Score =   12.85
           Number of Studies =      11
                          z  =    1.17
                    Pr > |z| =   0.243
                          z  =    1.09 (continuity corrected)
                    Pr > |z| =   0.276 (continuity corrected)

Egger's test
```

Std_Eff	Coef.	Std. Err.	t	P>|t|	[95% Conf.	Interval]
slope	6.62642	1.274128	5.20	0.001	3.744141	9.508698
bias	-5.259638	10.65494	-0.49	0.633	-29.36278	18.8435

Supplementary Figure 22: Publication bias for prevalence of ataxic cerebral palsy among children in Africa

```
--------------------------------------------------------------
Study omitted       |   Estimate       [95%  Conf.  Interval]
-------------------+------------------------------------------
Kakooza-Mwesige, A.  et al (2016)|5.04921822.83459457.2638421
Ngassa, E.A., et al  (2018)|5.35725453.2191267    7.4953823
Kakooza-Mwesige, A., et al  (2017)|5.64347273.81632117.470624
Karumuna, J. et al  (1990)|5.1184683 2.911072     7.3258643
El-Tallawy, H.N., et al (2008)|5.56538723.2123315 7.9184422
El-Tallawy, H.N., et al (2010)|5.35725453.2191267 7.4953823
Abas, O. et al (2017)|5.3027892       2.4487898    8.1567879
El-Tallawy, H.N., et al. (2014)|5.54304123.23262647.853456
Mangamba, D.K., et al. (2022)|5.35725453.2191267  7.4953823
Abas, O., F et al. (2020)|4.7081213  2.7490876    6.6671553
Tsige, S., et al.,  (2021)|5.5591874 3.3127768    7.8055983
Coombe, H.J. et al.  (2017)|5.09421832.8905923    7.2978439
Salih, K. et al.  (2020)|5.5061483    3.2735829    7.7387142
El Tallawy, H.N., et al. (2010)|5.35725453.21912677.4953823
Couper, J. (2002) |   5.3572545       3.2191267    7.4953823
Labena, F. et al (2021)|5.8133488     3.5591493    8.0675478
-------------------+------------------------------------------
Combined            |   5.3572546       3.2191267    7.4953824
--------------------------------------------------------------
```

Supplementary Figure 23: Sensitivity analysis for prevalence of ataxic cerebral palsy among children in Africa

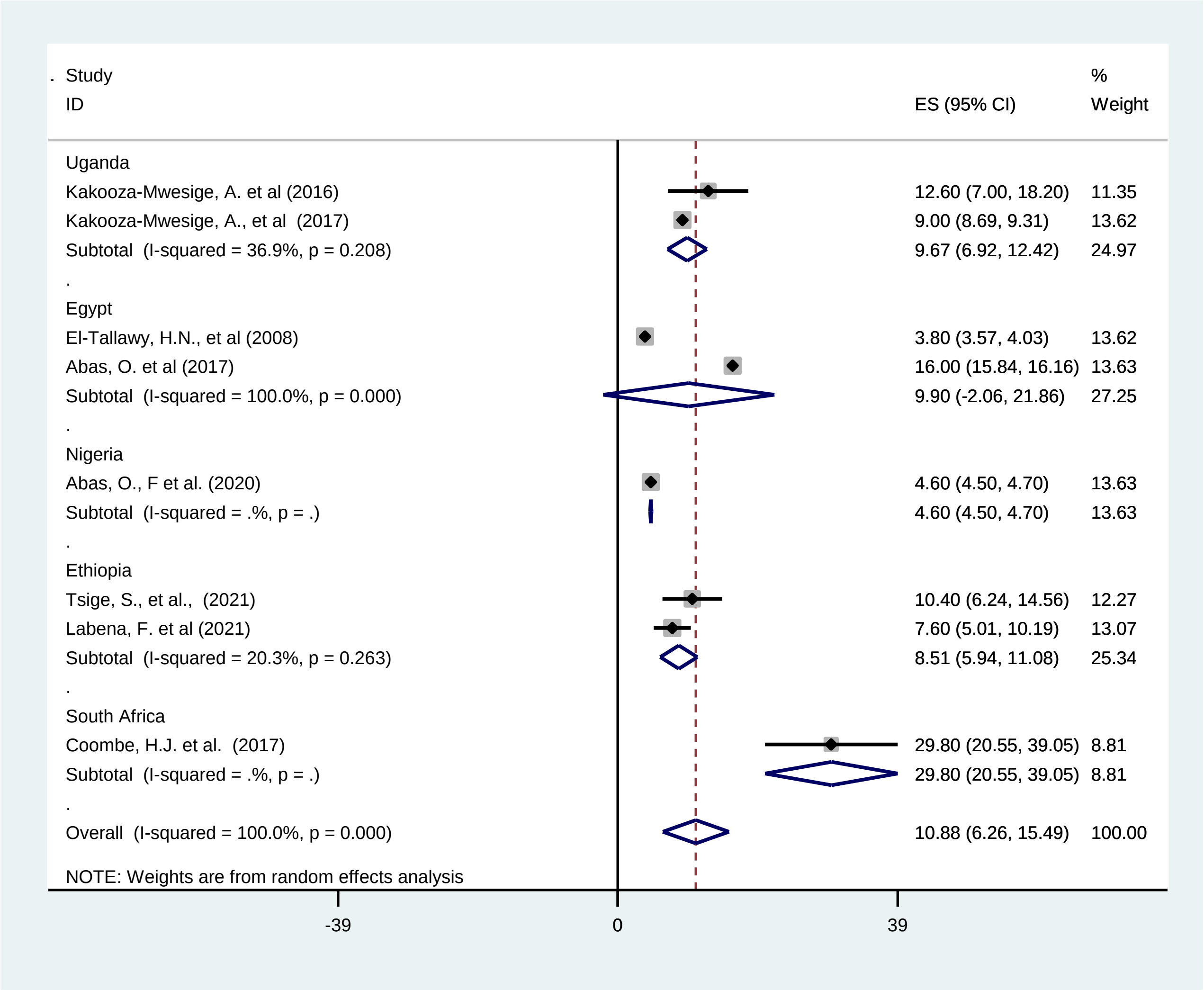

Supplementary Figure 24: Subgroup analysis for prevalence of dyskinetic cerebral palsy among children in Africa

```
Tests for Publication Bias

Begg's Test

  adj. Kendall's Score (P-Q) =        8
          Std. Dev. of Score =     8.08
           Number of Studies =        8
                          z  =     0.99
                  Pr > |z|  =    0.322
                          z  =     0.87 (continuity corrected)
                  Pr > |z|  =    0.386 (continuity corrected)

Egger's test
```

Std_Eff	Coef.	Std. Err.	t	P>|t|	[95% Conf.	Interval]
slope	6.74707	2.623172	2.57	0.042	.3283997	13.16574
bias	8.602378	23.60162	0.36	0.728	-49.14871	66.35346

Supplementary Figure 25: Publication bias for prevalence of dyskinetic cerebral palsy among children in Africa

Study omitted	Estimate	[95% Conf.	Interval]
Kakooza-Mwesige, A. et al	10.360925	6.0611768	14.660672
Ngassa, E.A., et al	10.581328	6.4993229	14.663334
Kakooza-Mwesige, A., et al	10.876186	6.2594166	15.492955
Karumuna, J. et al	10.581328	6.4993229	14.663334
El-Tallawy, H.N., et al	11.61076	6.88973	16.331789
Kakooza-Mwesige, A., et al	10.876186	6.2594166	15.492955
El-Tallawy, H.N., et al	10.581328	6.4993229	14.663334
Abas, O. et al	8.3329496	6.3774772	10.288422
El-Tallawy, H.N., et al.	10.581328	6.4993229	14.663334
Mangamba, D.K., et al.	10.581328	6.4993229	14.663334
Abas, O., F et al.	11.450811	6.929069	15.972553
Tsige, S., et al.,	10.603578	6.2815666	14.925589
Coombe, H.J. et al.	9.0316639	4.7908716	13.272456
Salih, K. et al.	10.581328	6.4993229	14.663334
El Tallawy, H.N., et al.	10.581328	6.4993229	14.663334
Couper, J.	10.581328	6.4993229	14.663334
Labena, F. et al	10.971025	6.6272697	15.314781
Combined	10.581328	6.4993229	14.663334

Supplementary Figure 26: Sensitivity analysis for prevalence of dyskinetic cerebral palsy among children in Africa

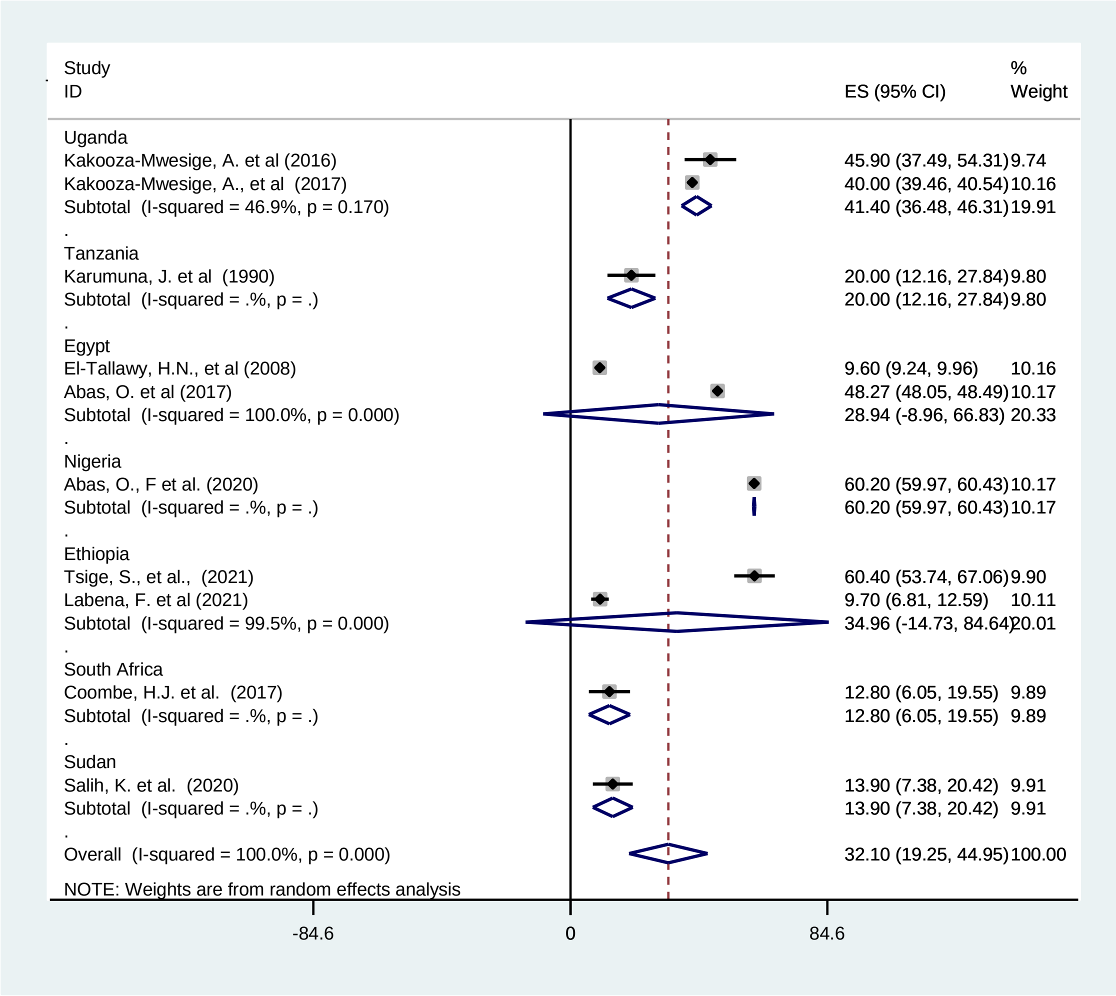

Supplementary Figure 27: Subgroup analysis for prevalence of dyskinetic cerebral palsy among children in Africa

```
Tests for Publication Bias

Begg's Test

  adj. Kendall's Score (P-Q) =         5
          Std. Dev. of Score =     11.18
           Number of Studies =        10
                          z  =      0.45
                    Pr > |z| =     0.655
                          z  =      0.36 (continuity corrected)
                    Pr > |z| =     0.721 (continuity corrected)

Egger's test
```

Std_Eff	Coef.	Std. Err.	t	P>|t|	[95% Conf.	Interval]
slope	49.33678	7.592411	6.50	0.000	31.82865	66.84491
bias	-22.0203	33.48909	-0.66	0.529	-99.24628	55.20568

Supplementary Figure 28: Publication bias for prevalence of bilateral cerebral palsy among children in Africa

```
----------------------------------------------------------------------
 Study omitted        |   Estimate        [95%  Conf.  Interval]
----------------------+-----------------------------------------------
 Kakooza-Mwesige, A.  et al (2016)|30.60922117.08054944.137894
 Ngassa, E.A., et al  (2018)|32.09887319.249691    44.948055
 Kakooza-Mwesige, A., et al  (2017)|31.20385616.75551445.652199
 Karumuna, J. et al  (1990)|33.412651 19.884605    46.940701
 El-Tallawy, H.N., et al (2008)|34.88831728.311319 41.465317
 El-Tallawy, H.N., et al (2010)|32.09887319.249691 44.948055
 Abas, O. et al (2017)|30.273321       10.734913    49.811729
 El-Tallawy, H.N., et al. (2014)|32.09887319.24969144.948055
 Mangamba, D.K., et al. (2022)|32.09887319.249691  44.948055
 Abas, O., F et al. (2020)|28.924917  13.944308    43.905525
 Tsige, S., et al.,  (2021)|28.990797 15.450568    42.531025
 Coombe, H.J. et al.  (2017)|34.21655320.686209    47.746899
 Salih, K. et al.  (2020)|34.099903   20.567814    47.631992
 El Tallawy, H.N., et al. (2010)|32.09887319.24969144.948055
 Couper, J. (2002) |   32.098873      19.249691    44.948055
 Labena, F. et al (2021)|34.619461    21.109371    48.129555
----------------------+-----------------------------------------------
 Combined             |   32.098874      19.24969     44.948057
----------------------------------------------------------------------
```

Supplementary Figure 29: Sensitivity analysis for prevalence of bilateral cerebral palsy among children in Africa

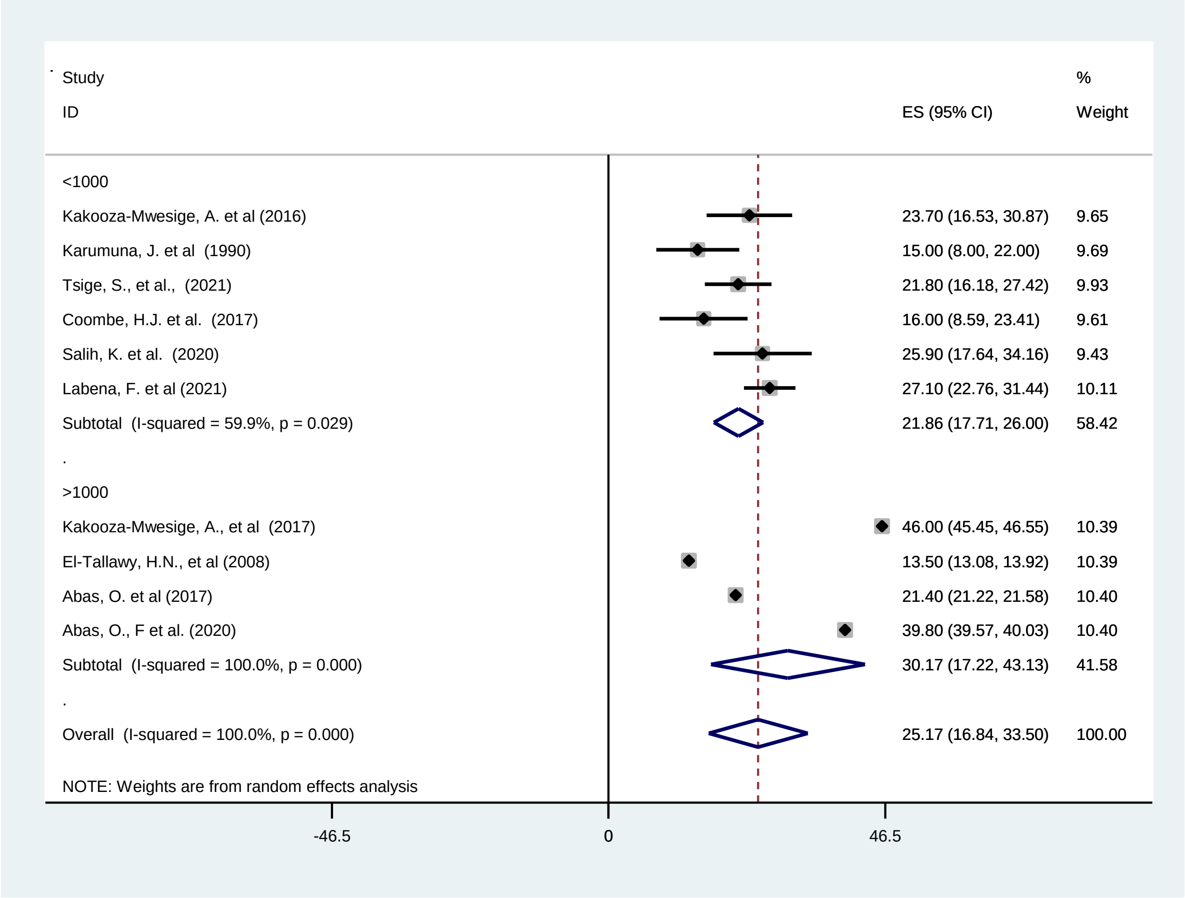

Supplementary Figure 30: Subgroup analysis for prevalence of unilateral cerebral palsy among children in Africa

```
Tests for Publication Bias

Begg's Test

  adj. Kendall's Score (P-Q) =         1
          Std. Dev. of Score =     11.18
           Number of Studies =        10
                          z  =      0.09
                    Pr > |z| =     0.929
                          z  =      0.00 (continuity corrected)
                    Pr > |z| =     1.000 (continuity corrected)

Egger's test
```

Std_Eff	Coef.	Std. Err.	t	P>|t|	[95% Conf.	Interval]
slope	27.51717	4.640105	5.93	0.000	16.81707	38.21727
bias	2.770173	22.00451	0.13	0.903	-47.97231	53.51266

Supplementary Figure 31: Publication bias assessment for prevalence of unilateral cerebral palsy among children in Africa

```
------------------------------------------------------------------
 Study omitted      |   Estimate        [95%  Conf.   Interval]
--------------------+---------------------------------------------
 Kakooza-Mwesige, A. et al (2016)|25.32942216.55998434.098858
 Ngassa, E.A., et al  (2018)|25.17223 16.839808    33.504654
 Kakooza-Mwesige, A., et al  (2017)|22.76149714.2562431.266756
 Karumuna, J. et al  (1990)|26.263254 17.494118    35.032391
 El-Tallawy, H.N., et al (2008)|26.52597217.717869 35.334076
 El-Tallawy, H.N., et al (2010)|25.1722316.839808  33.504654
 Abas, O. et al (2017)|25.552122        14.991363   36.112881
 El-Tallawy, H.N., et al. (2014)|25.1722316.839808 33.504654
 Mangamba, D.K., et al. (2022)|25.1722316.839808   33.504654
 Abas, O., F et al. (2020)|23.487022  15.176888    31.797157
 Tsige, S., et al.,  (2021)|25.543716 16.759541    34.327892
 Coombe, H.J. et al.  (2017)|26.14703817.381573    34.912502
 Salih, K. et al.  (2020)|25.096329   16.338161    33.854496
 El Tallawy, H.N., et al. (2010)|25.1722316.839808 33.504654
 Couper, J. (2002) |    25.17223        16.839808    33.504654
 Labena, F. et al (2021)|24.955036    16.15757     33.752506
--------------------+---------------------------------------------
 Combined           |    25.17223        16.839808    33.504653
------------------------------------------------------------------
```

Supplementary Figure 32: Sensitivity analysis for prevalence of unilateral cerebral palsy among children in Africa

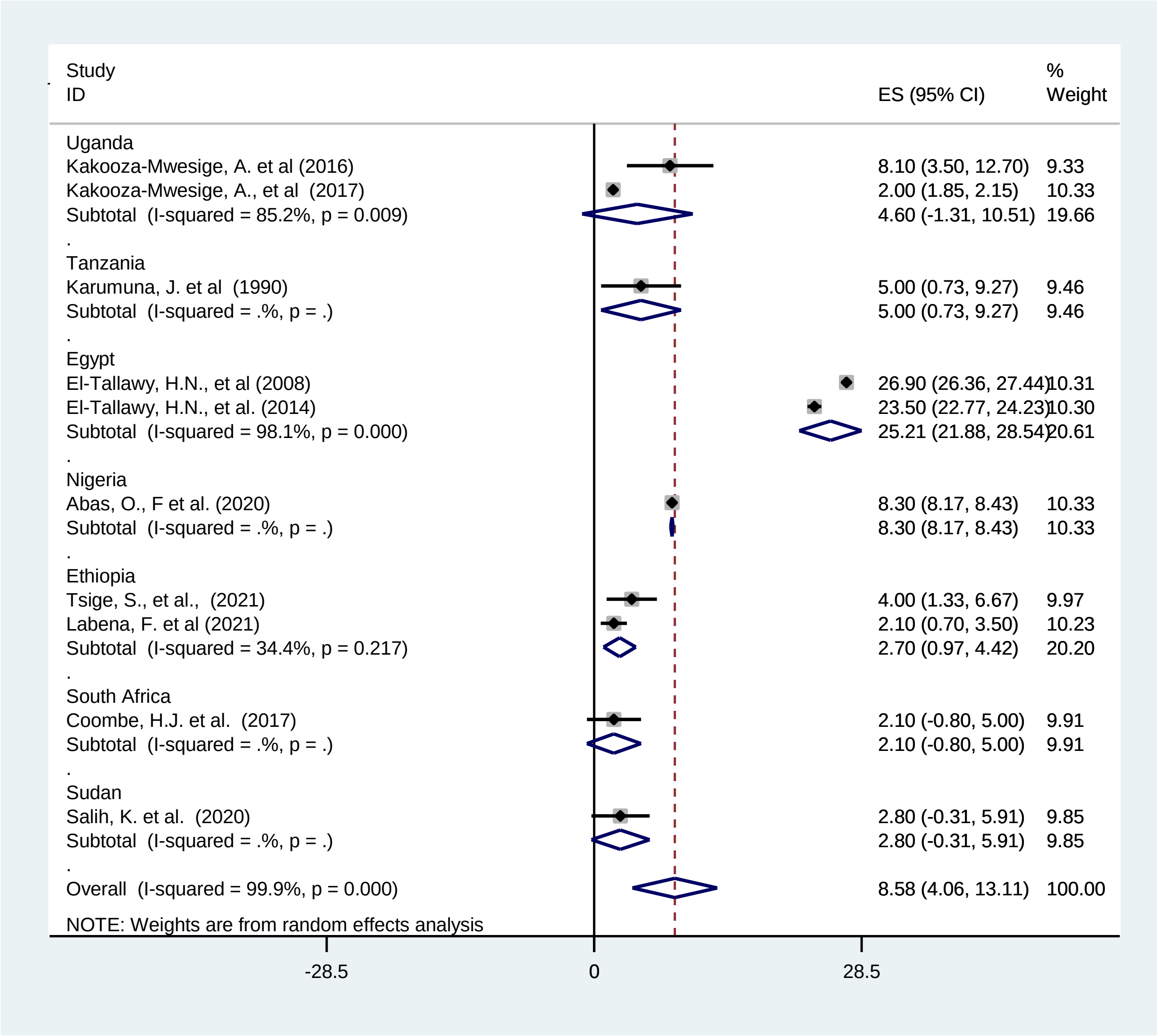

Supplementary Figure 33: Subgroup analysis for prevalence of mixed type cerebral palsy among children in Africa

```
Tests for Publication Bias

Begg's Test

  adj. Kendall's Score (P-Q) =        1
          Std. Dev. of Score =    11.18
           Number of Studies =       10
                          z  =     0.09
                    Pr > |z| =    0.929
                          z  =     0.00 (continuity corrected)
                    Pr > |z| =    1.000 (continuity corrected)

Egger's test
```

Std_Eff	Coef.	Std. Err.	t	P>|t|	[95% Conf.	Interval]
slope	5.589804	2.255455	2.48	0.038	.3887155	10.79089
bias	11.01558	14.45599	0.76	0.468	-22.31999	44.35115

Supplementary Figure 34: Publication bias for prevalence of mixed type cerebral palsy among children in Africa

```
-----------------------------------------------------------------------
 Study omitted        |   Estimate       [95%  Conf.  Interval]
----------------------+------------------------------------------------
 Kakooza-Mwesige, A. et al (2016)|8.63342763.879789813.387066
 Ngassa, E.A., et al  (2018)|8.58377934.060061      13.107497
 Kakooza-Mwesige, A., et al  (2017)|9.25693611.737354316.776518
 Karumuna, J. et al  (1990)|8.9579554 4.2005887     13.715322
 El-Tallawy, H.N., et al (2008)|6.51155382.8653743 10.157733
 El-Tallawy, H.N., et al (2010)|8.58377934.060061   13.107497
 Abas, O. et al (2017)|8.5837793       4.060061     13.107497
 El-Tallawy, H.N., et al. (2014)|6.88074112.437846411.323636
 Mangamba, D.K., et al. (2022)|8.58377934.060061    13.107497
 Abas, O., F et al. (2020)|8.5262775  -1.4820638    18.534618
 Tsige, S., et al.,  (2021)|9.0909719 4.3157659     13.866178
 Coombe, H.J. et al.  (2017)|9.29655934.5253563     14.067761
 Salih, K. et al.  (2020)|9.2152557    4.4458628     13.98465
 El Tallawy, H.N., et al. (2010)|8.58377934.060061 13.107497
 Couper, J. (2002) |   8.5837793       4.060061     13.107497
 Labena, F. et al (2021)|9.3211269    4.5249085     14.117346
----------------------+------------------------------------------------
 Combined             |   8.5837791      4.0600609    13.107497
-----------------------------------------------------------------------
```

Supplementary Figure 35: Sensitivity analysis for prevalence of mixed type cerebral palsy among children in Africa

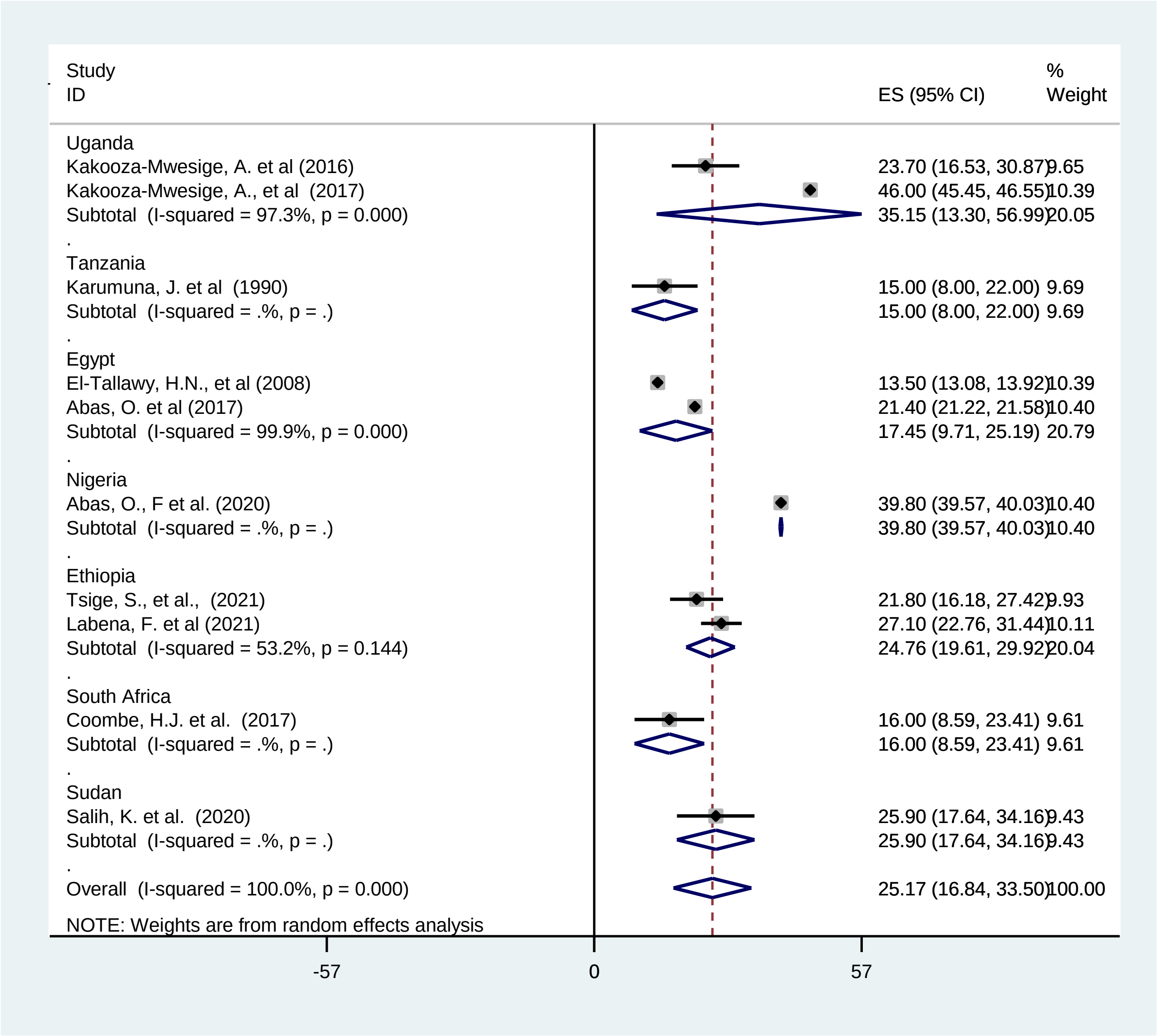

Supplementary Figure 26: subgroup analysis for prevalence of unilateral cerebral palsy among children in by country in Africa

Click here to access/download
Supporting Information
Supplementary Materials.docx

Checklist

Click here to access/download
Supporting Information
PRISMA_2020_checklist.pdf
