# Supplementary material for: Magnitude and clinical characteristics of cerebral palsy among children in Africa: A systematic review and meta-analysis
Source: PLOS Glob Public Health. 2024 Jun 21;4(6):e0003003. doi: 10.1371/journal.pgph.0003003 (PMC11192420; doi:10.1371/journal.pgph.0003003)
Supplement: Attachment — Submitted filename: Point by point response.docx [file pgph.0003003.s022.docx]

**Point by point response**

PGPH-D-23-02244

**Magnitude and clinical characteristics of cerebral palsy among children in Africa: A systematic review and Meta-analysis**

**Dear Shobhana Nagraj (Academic Editor),**

Many thanks for letting us know such invaluable comments on our submission-" Magnitude and clinical characteristics of cerebral palsy among children in Africa: A systematic review and Meta-analysis". Each point was very helpful for making our paper better and suitable for publication in your prestigious journal. As such, we have tried to amend the manuscript in light of all your and reviewers’ comments and the journal guideline. We included the point-to-point response to each comment as below. We have highlighted the track changes in the manuscript and attached as “Manuscript with track changes”. We have also attached a clean version manuscript.

**Journal Requirements**
**Editor Comment 1.** We noticed you have some minor occurrence of overlapping text with the following previous publication(s), which needs to be addressed:

-<http://dx.doi.org/10.1136/bmjopen-2020-039200>
-<http://dx.doi.org/10.1016/j.spen.2014.01.001>
-<https://doi.org/10.3390/brainsci12070859>

In your revision ensure you cite all your sources (including your own works), and quote or rephrase any duplicated text outside the methods section. Further consideration is dependent on these concerns being addressed.

**Authors Response:** Many thanks for raising such an important point. We have now rewritten the sections which have similarities with previous works, and we also cited all sources (including our own works).

**Editor Comment** 2. Please provide separate figure files in .tif or .eps format only and remove any figures embedded in your manuscript file. Please also ensure all files are under our size limit of 10MB.

For more information about figure files please see our guidelines:

<https://journals.plos.org/globalpublichealth/s/figures> 
<https://journals.plos.org/globalpublichealth/s/figures#loc-file-requirement>

**Authors Response:** Thanks! We have removed supplementary figures/tables from the manuscript and we uploaded separately as 'Supporting Information': **Page 39-40: Line 508-529**

**Editor Comment** 3. We have noticed that you have uploaded Supporting Information files, but you have not included a list of legends. Please add a full list of legends for your Supporting Information files after the references list.

**Authors Response:** Thanks so much! We have added a legend for supplementary tables and figures just after the reference. **Page 39-40: Line 508-529**

**Editor Comment** 4. We notice that your supplementary [figures/tables] are included in the manuscript file. Please remove them and upload them with the file type 'Supporting Information'. Please ensure that each Supporting Information file has a legend listed in the manuscript after the references list.

**Authors Response:** Thanks! We have removed supplementary figures/tables from the manuscript and we uploaded separately as 'Supporting Information'.

Additional Editor Comments (if provided):
Dear Authors,
Thank you for submitting your manuscript to our journal. Please note Reviewer 1's feedback, which recommends a major revision, with detailed feedback and outlining the necessary changes required for the manuscript. We look forward to receiving the revised draft.
Yours sincerely,
[Note: HTML markup is below. Please do not edit.]

**Reviewers' comments:**

Reviewer's Responses to Questions

**Comments to the Author**
1. Does this manuscript meet PLOS Global Public Health’s [publication criteria](https://journals.plos.org/globalpublichealth/s/criteria-for-publication)? Is the manuscript technically sound, and do the data support the conclusions? The manuscript must describe methodologically and ethically rigorous research with conclusions that are appropriately drawn based on the data presented.

Reviewer #1: Yes

Reviewer #2: Yes

2. Has the statistical analysis been performed appropriately and rigorously?

 Reviewer #1: Yes

Reviewer #2: Yes

3. Have the authors made all data underlying the findings in their manuscript fully available (please refer to the Data Availability Statement at the start of the manuscript PDF file)?
The [PLOS Data policy](https://journals.plos.org/globalpublichealth/s/data-availability) requires authors to make all data underlying the findings described in their manuscript fully available without restriction, with rare exception. The data should be provided as part of the manuscript or its supporting information, or deposited to a public repository. For example, in addition to summary statistics, the data points behind means, medians and variance measures should be available. If there are restrictions on publicly sharing data—e.g. participant privacy or use of data from a third party—those must be specified.

 Reviewer #1: Yes

Reviewer #2: Yes

4. Is the manuscript presented in an intelligible fashion and written in standard English?
PLOS Global Public Health does not copyedit accepted manuscripts, so the language in submitted articles must be clear, correct, and unambiguous. Any typographical or grammatical errors should be corrected at revision, so please note any specific errors here.

 Reviewer #1: No

Reviewer #2: Yes

5. Review Comments to the Author
Please use the space provided to explain your answers to the questions above. You may also include additional comments for the author, including concerns about dual publication, research ethics, or publication ethics. (Please upload your review as an attachment if it exceeds 20,000 characters)

**For Reviewer #1:**

**Dear Reviewer 1,**

Thank you so much for taking time to take a through look at our work and giving us such in-depth and invaluable feedback. All authors appreciated the way you commented the manuscript and we agreed with almost all of your comments and believe that we have amended the manuscript in light of all your comments. Many thanks again for your insightful feedback. We have indicated the point-by-point response for each of your comments below.

**Review Report**
Title: Magnitude and clinical characteristics of cerebral palsy among children in Africa: A systematic review and Meta-analysis
Date: December 29,2023
**General comment**
**Reviewer comment:** Dear editor, thank you for considering me to review this manuscript. This paper requires a lot of editorial work to improve the logical flow of information and coherence in the description of methods, results of the study, discussion, and recommendation. The draft also requires the correction of grammatical errors. Furthermore, too many results were reported, which makes it difficult to follow and understand. Authors **Response:** Thank you so much for such fantastic comment. All authors re-edited the manuscript to remove the grammatical errors and improve the manuscript. Besides, we have send the manuscript to English language experts in our university and they have proof edited it. Thus, we believe now the manuscript has been greatly improved considering your and reviewers comments.

**Reviewer comment:** Reporting the characteristics of cerebral palsy at once seems ideal than separately writing them. There are also too many figures and supplementary materials. This is again the result of the wide result section. Generalizing the findings is also the main concern here.

**Authors Response:** Thank you so much for your comment. The current study has two objectives. The first is to assess the prevalence of Cerebral Palsy, and the second is to assess the clinical characteristics- types of cerebral palsy when we say clinical characteristics. Right you are, there are too many figures-Now we have tried to reduce some figures by omitting some unnecessary statistical details as per your comment.

**Reviewer comment:**  With only 15 studies included from a few countries, how can we draw inferences for all African countries?
**Authors Response:** Many thanks for this invaluable insight. Although we have tried to extensively search articles from all countries in Africa, we able to found 15 articles with (n = 498406 patients) from few countries. We agree that this may affect the generalizability of the pooled findings. We have indicated this in the limitation section. **Page 32-33: Line 331-337**

**Specific comment**
**Abstract**
**Reviewer comment 1.** Background and objective are not well articulated
**Authors Response:** Many thanks for your insight. We have revised the background and objective section of the abstract as per your comment. **Page 2: Line 16-18**

**Reviewer comment** 2. What are the criteria to consider prevalence and clinical characteristics of cerebral palsy as primary and secondary outcome?

**Authors Response:** Prevalence of cerebral palsy was considered as the proportion of children with cerebral palsy among 1000 risk population. Clinical characteristics of cerebral palsy indicates types of cerebral palsy spastic, athetoid (dyskinetic), ataxic, hypotonic, and mixed type. **Page 8 Line 118-123**

**Reviewer comment** 3. Clearly present the information about the types of cerebral palsy. It lacks logical flow of the information to be presented.
**Authors Response:** There are **five major types** of cerebral palsy: spastic, athetoid (dyskinetic), ataxic, hypotonic, and mixed type. **Page 3: Line 18-19**

**Reviewer comment** 4. Consider the keywords
**Authors Response:** Thanks so much! we have revised this section. **Page 3 Line 40**

**Reviewer comment** 5. I’m not sure if the PLOS Global Public Health requires the Strengths and limitations of this study to written before background. I think this is a manuscript prepared for journals like BMJ Open. Follow the guideline of the journal.

**Authors Response:** Many thanks for your invaluable insight. We have now revised the manuscript as per the journal guideline. As such, we have removed the strengths and limitations of this study to written before background.
**Background**
**Reviewer comment** 1. Abbreviation/acrimony issues
**Authors Response:** Thanks! We have checked all abbreviation/acrimony throughout the manuscript and revised accordingly.

**Reviewer comment** 2. Add the significance of the reviews finding for concerned stakeholders

**Authors Response:** Thank you so much for this invaluable comment. We have included the gap of available evidence and significance of the current review at the end of the introduction as a closing paragraph. Introduction Page 6: Line 88-93
**Method**
**Reviewer comment** 1. Why you’re interested to present the search strategy report of PubMed database only. It is written as…. Thus, the PubMed search combines #1 AND #2 AND #3 AND #4 AND #5. This should be removed.

**Authors Response:** Thank you so much for your comment. We have removed the indicated statement and revised this section.
**Reviewer comment** 2. Inclusion and exclusion criteria; specify PICO
**Authors Response:** We have revised the inclusion and exclusion criteria specifying PICO when applicable in light of your comments. ***Page 8 Line 118-123***

**Reviewer comment** 3. Why is it important to do subgroup analysis using study design? What value does it add for concerned stakeholders?
**Authors Response:** Thank you so much for your insightful comment. We have now reduced unnecessary figures from the supplementary figures and revised the figures order in the manuscript as per your comment.

**Results**
**Reviewer comment** 1. Improve the quality of PRISMA –flow diagram

**Authors Response:** Thank so much for your invaluable comment. We have adjusted the diagram to improve the quality of the diagram. **Page 10: Line 150-160.**
**Reviewer comment** 2. Under Characteristics of included studies; remove this statement…. Table 1: summarizes the characteristics of the 15 included studies in this systematic review and meta-analysis… and further information that can summarize the information written in table 1

**Authors Response:** We have removed the indicated statement in light of your comment.
**Reviewer comment** 3. Consider clearing out extraneous lines from tables.
**Authors Response:** Thanks! We now removed the extraneous lines from tables: **Page 11-12 : Table 1**

**Reviewer comment** 4. Think about removing or clipping unnecessary shade/borders from figures, as this affects quality after publication.
**Authors Response:** Thanks! We now removed unnecessary shade/borders from figures.

**Reviewer comment** 5. The country-by-country subgroup study yielded very detailed results. Just list the highest and lowest prevalence; figure 3 presents the results in great detail.

**Authors Response:** Thank you so much for your insightful comment. We have now reduced unnecessary figures from the supplementary figures and revised the figures order in the manuscript as per your comment.
**Reviewer comment** 6. The caption for the figure displaying subgroup analysis for the prevalence of cerebral palsy among children in African nations was written figure 3, however it is cited in the paper as Supplementary Fig. 1. This may affect the whole sup. Files order

**Authors Response:** Thanks for your insight. We have revised the figure orders in the supplementary files and in the main text in light of your comment.
**Reviewer comment** 7. How important is it to perform subgroup analysis for every cerebral palsy characteristic? Just a small number of studies were used in this particular study. This may have an impact on the subgroup analysis's outcome. You included too many figures in the manuscript by writing a detailed report on the findings and providing extra information. For each kind of cerebral palsy, reports included pooled prevalence, subgroup analysis, publication bias, and sensitivity analysis. There is too much information here, making it difficult to interpret the outcome.

**Authors Response:** Thank you so much for your insightful comment. We have now reduced unnecessary figures from the supplementary figures and revised the figures order in the manuscript as per your comment.

**Reviewer #2**

**Dear Oluwayemi Joshua Bamikole (Reviewer 2),**

Thank you so much for taking time to take a through look at our work and giving us such in-depth and invaluable feedback. All authors appreciated the way you commented the manuscript and we agreed with all of your comments and believe that we have amended the manuscript in light of all your comments. Many thanks again for your insightful feedback. We have indicated the point-by-point response for each of your comments below.

**Reviewer comment:** The author successfully presented the article in a scientific, clear, and concise approach, demonstrating a profound understanding of complex concepts while effectively communicating them to the target audience

**Reviewer comment 1**: **Objective**: The study aims to

**Authors Response**: Thank you so much. We have adjusted the objective in light of your comment. **Page 2 Line 19**

**Reviewer comment 2**: **Method**: what of the PROSPERO registration number for this review

**Authors Response**: We have submitted the protocol for PROSPERO and it is under consideration with ssubmission ID=516993

**Reviewer comment 3**: **Conclusion**: The prevailing incidence of cerebral palsy in Africa surpasses the reported rates in developed nations. Predominantly, spastic and quadriplegic forms of cerebral palsy emerge as the most frequently observed subtypes. In light of these observations, it is recommended that both governmental and non-governmental entities channel their initiatives toward the strategic focus on preventive measures, early detection strategies, and comprehensive management protocols for cerebral palsy across the African continent.

**Authors Response**: Thank you so much for this invaluable insight. We have revised the conclusion section of the manuscript as per your comment. **Page 3 Line 36-39**

**Reviewer comment 4**: Respectively\

**Authors Response**: Thanks. We have now amended it accordingly. **Page 4 Line 61**

**Reviewer comment 5**: What of Prospero registration number for this review

**Authors Response**: We have submitted the protocol for PROSPERO and it is under consideration with ssubmission ID=516993
